# *In-silico* identification of host-key-genes associated with dengue-virus-infections highlighting their pathogenetic mechanisms and therapeutic agents

**DOI:** 10.1371/journal.pone.0333509

**Published:** 2025-10-07

**Authors:** Md. Abdul Latif, Md. Al Noman, Reaz Ahmmed, Md. Sanoar Hossain, Md. Foysal Ahmed, Md. Al Amin Pappu, Md. Shariful Islam, Tasfia Noor, Md. Hadiul Kabir, Md. Nurul Haque Mollah

**Affiliations:** 1 Department of Statistics, Bioinformatics Lab (Dry), University of Rajshahi, Rajshahi, Bangladesh; 2 Department of Biochemistry and Molecular Biology, University of Rajshahi, Rajshahi, Bangladesh; 3 Institute of Bangladesh Studies (IBS), University of Rajshahi, Rajshahi, Bangladesh; 4 Department of Computer Science and Engineering (CSE), Rajshahi University of Engineering and Technology (RUET), Rajshahi, Bangladesh; AMET University, INDIA

## Abstract

Dengue fever (DF), a potentially fatal mosquito-transmitted viral disease caused by dengue virus (DENV) infections (DENVI), stands as the predominant arthropod-borne viral illness worldwide, presenting a significant global health challenge. DENV-mediated proteins/proteases interact with host proteins to develop the infection. Despite the severity of DENVI, the infection-causing host key-genes (hKGs), their pathogenetic processes, and inhibitors/activators are not yet rigorously investigated. This study aimed to disclose DENVI-causing hKGs, highlighting their pathogenetic mechanisms and therapeutic agents. At first, 115 host differentially expressed genes (hDEGs) between DENVI and control samples were identified by employing the LIMMA statistical approach. Through protein-protein interaction (PPI) network analysis, the top nine hDEGs (CDK1, BIRC5, TYMS, KIF20A, CCNB2, CDC20, AURKB, TK1, and PTEN) were detected as the infection-causing hGBs or host key-genes (hKGs). Among these hKGs, six genes (CDK1, BIRC5, TYMS, KIF20A, CCNB2, and TK1) have been emphasized as the DENVI-causing genes by the literature review. Functional enrichment analysis showed how hKGs orchestrate viral infection processes by disrupting cell cycles and immune responses. CDK1 and AURKB divert mitotic machinery to support viral replication, while PTEN and BIRC5 inhibit MAVS-MDA5 pathways to suppress interferon responses. In the nucleus, CDK1 and TYMS manipulate host transcription to favor viral processes. Key pathways identified through KEGG analysis include cell cycle and p53 signaling, explaining DENV-induced thrombocytopenia and dysregulated apoptosis. The regulatory network analysis identified five transcription factors (FOXC1, GATA2, RELA, TP53, PPARG) as the transcriptomic regulators of hKGs. The regulators FOXC1 and RELA influence EMT and inflammatory responses, and PPARG’s involvement in lipid metabolism correlates with Dengue Shock Syndrome severity, while miR-103a-3p enhances viral replication by targeting the OTUD4/p38 MAPK pathway. Finally, hKGs-guided three drug candidates (ENTRECTINIB, IMATINIB, and QL47) were selected by molecular docking analysis. These findings provide valuable insights that could significantly impact dengue fever diagnosis and treatment strategies.

## 1. Introduction

**Fig 1:**
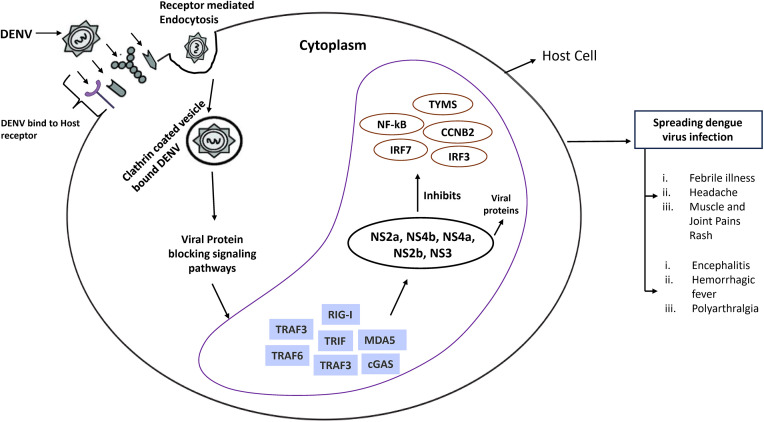
A schematic diagram of the link of DENV infection with host genes/proteins.

Dengue (DEN) is a rapidly emerging infectious disease transmitted by arthropods and caused by the dengue virus (DENV) [1]. It is a significant global health threat affecting approximately two-thirds of the world’s population. Dengue virus (DENV) is a single-stranded positive RNA virus belonging to the Flavivirus family [2,3]. This RNA virus can trigger various disease severities, ranging from mild symptoms to life-threatening shock syndrome. Despite its widespread impact, current treatment options remain limited, with no specific antiviral drugs and limited vaccine availability. DENVs are categorized in to four distinct serotypes, each with its unique genetic structure recorded in the literature. These variants are officially classified as DENV-1 through DENV-4, with respective reference sequences MW288036.1, KM204118.1, MW288040.1, and KJ596664.1, respectively [4]. DENV orchestrates complex manipulation of host cells through multiple biological pathways. Flaviviruses (e.g., DENV) use host cell functions to complete their life cycle, manipulating RNA metabolism and immune responses. After infection of the host, DENV mainly attacks and replicates in dendritic cells and also infects macrophages, lymphocytes, and monocytes. The virus enters cells through receptor-mediated endocytosis, using surface molecules like Fc receptors, glycosaminoglycans (GAGs), CD14-associated molecules, heparan sulfate, and lectin-like receptors such as DC-SIGN [2,5]. DENV enters the cell through clathrin-coated vesicles [6,7]. After entering through cell receptors, DENV uses host cellular machinery to produce its components. The viral nonstructural proteins strategically disrupt immune responses to facilitate infection spread. NS3 and NS4a block RIG-I translocation to mitochondria, while NS2a and NS4b inhibit TBK1 activation, preventing MAVS pathway signaling and IFN-β production. NS2b suppresses immunity by promoting GAS degradation through autophagy-lysosome mechanisms, blocking mitochondrial DNA sensing, and proteolytically cleaving STING to prevent interferon production [8]. Moreover, DENV and host-gene interactions involve the direct modulation of cellular pathways, such as epigenetic reprogramming (NS1–DIDO1), transcriptional control (NS5-mediated STAT2 degradation), and the hijacking of RNA splicing and translation. In contrast, downstream host responses, including interferon signaling, cytokine storms, metabolic reprogramming, and disruption of stress granules, are activated in response to infection. While intended as defense mechanisms, these responses are frequently subverted by DENV to facilitate immune evasion, enhance replication, and drive disease severity [2,9]. A schematic diagram about how DENV genes interact with host genes to develop infection and related symptoms was displayed in **Fig 1**.

Despite the severity of DENVI, the infection-causing host key-genes (hKGs), their pathogenetic mechanisms, and inhibitors/activators are not yet rigorously investigated. Therefore, it is essential to investigate DENVI-causing host key genes (hKGs) more accurately to disclose pathogenetic mechanisms and therapeutic agents. However, it may be difficult to detect most potential hKGs and therapeutic drugs from a huge number of alternatives, by the wet-lab experiments only, since it is laborious, time-consuming, and costly. In this case, bioinformatics approaches are playing a significant role [10–12]. There are few bioinformatics-based studies that explored DENVI-causing hKGs, where one study considered only one dataset [13] and two studies considered three datasets [14,15]. However, their hKGs sets were inconsistent, and they did not investigate pathogenetic mechanisms and inhibitors/activators of hKGs rigorously. In order to explore more consistent hKGs, a study considered six datasets [16] from the NCBI database. However, in this study, they did not correctly classify the samples in case and control groups. For example, the dataset with NCBI accession ID GSE51808 consisted of 28 cases, 19 convalescent and 9 control samples, but the authors incorrectly classified all the convalescent samples to the case group, though it should have been classified to the control group (see UMAP plot in S1 Fig). Another dataset with accession ID GSE28991 was not exactly of dengue versus control samples, but they considered early infection samples (Less than 72 hours after symptoms) as the samples of the control group. These classification errors may produce a misleading hDEGs set. Therefore, in this study, we considered four transcriptomics datasets that consisted of clearly defined dengue versus control samples to explore DENVI-causing hKGs, highlighting their molecular mechanisms and therapeutic candidates through bioinformatics analysis.

## 2. Methodology

### 2.1. Data source and description

To explore DENVI-causing host key-genes (hKGs), four publicly accessible gene expression profiles datasets were downloaded from the National Center of Biotechnology Information (NCBI) Gene Expression Omnibus (GEO) database [17] with accession numbers GSE84331 [15], GSE51808 [16], GSE28405 [16] and GSE43777 (GPL570) [14]. The detailed descriptions of these datasets are given in **Table 1**.

**Table 1:** The detailed descriptions of the gene expression profile datasets.

GEO Dataset	Country	Sample	Control	Case	Platform
GSE84331	USA	12	5	7	[HG-U133_Plus_2] Affymetrix Human Genome U133 Plus 2.0 Array
GSE51808	Brazil	56	28	28	[HT_HG-U133_Plus_PM] Affymetrix HT HG-U133 + PM Array Plate
GSE28405	Singapore	119	26	93	GPL2700: Sentrix HumanRef-8 Expression BeadChip
GSE43777	USA	101	24	77	GPL570: [HG-U133_Plus_2] Affymetrix Human Genome U133 Plus 2.0 Array

### 2.2. Identification of host differentially expressed genes (hDEGs)

In order to explore host differentially expressed genes (hDEGs) between normal and DENV groups, we considered a statistical LIMMA (Linear Models for Microarray) approach [18]. We employed the GEO2R web tool [19], which utilizes the LIMMA (Linear Models for Microarray) package, to identify hDEGs for each of the four datasets mentioned in section 2.1. The LIMMA method uses an empirical Bayesian approach to improve variance estimation, making it well-suited for small sample sizes [18,20]. GEO2R relies on the series matrix files provided by original submitters, which are typically pre-processed using platform-specific normalization methods, including robust multi-array average (RMA) [21]. After identifying hDEGs within each dataset separately, our analysis used established threshold criteria (|log_2_FC| > 1, adjusted *p*-value<0.05) to select the hDEGs for the individual dataset.

### 2.3. PPI network analysis of hDEGs for identification of hKGs

Cellular proteins function through interactions with other proteins, and understanding these interaction networks provides deeper insights into protein roles [22,23]. We utilized STRING v11, an online database and analysis tool [24], to construct protein-protein interaction (PPI) networks from differentially expressed genes [25]. This platform combines experimental data with predicted interactions, providing confidence scores and structural information. For enhanced network visualization, we analyzed these interactions using Cytoscape software. To identify key genes, we employed Cytoscape’s CytoHubba plugin [26], which offers multiple topological analysis methods. Our study incorporated eight different measures, including Maximal Clique Centrality (MCC), MNC, Degree, Closeness, Betweenness, Edge Percolated Component (EPC), Stress, and Bottleneck, as each capture different aspects of network importance. This integrative approach improves robustness by identifying genes with high connectivity, regulatory influence, and structural significance within the gene regulatory network. We selected the top-ranked DEGs as the host key-genes (hKGs) based on the higher frequency in the lists of top-ranked 10 DEGs selected for each of the topological measures.

### 2.4. Disclosing pathogenetic processes of hKGs

To disclose pathogenetic processes of hKGs, we performed hKGs regulatory network analysis with transcription factors (TFs) proteins and microRNAs (miRNAs), and enrichment analysis with gene ontology (GO) terms and KEGG pathways as introduced in sub-sections 2.4.1 and 2.4.2.

#### 2.4.1. Regulatory network analysis of hKGs to detect key regulators.

We conducted a comprehensive analysis of regulatory networks to understand how host key genes (hKGs) are controlled at both transcriptional and post-transcriptional levels. Our investigation involved studying interactions with transcription factors (TFs) and microRNAs (miRNAs) [27]. For transcriptional regulation, we used the JASPAR database [28] to examine TF-KG interactions, identifying the most significant TFs controlling our key genes. To understand post-transcriptional regulation, we analyzed miRNA-KG relationships using the TarBase database [29], with NetworkAnalyst [30] helping to reproduce these connections. This analysis allowed us to identify the most influential miRNAs regulating our key genes. We utilized Cytoscape for creating visual representations of these complex interaction networks [31], providing clear insights into the regulatory mechanisms controlling our identified key genes at multiple levels.

#### 2.4.2. GO and KEGG pathway enrichment analysis of hKGs.

We used Gene Ontology (GO) classification to annotate differentially expressed genes (hDEGs), examining three key aspects: Biological Process (BP), Molecular Function (MF), and Cellular Component (CC) [32,33]. This classification helps illuminate how genes function at the molecular level, their cellular roles, and their location within cells. To understand metabolic pathways involving these genes, we employed the Kyoto Encyclopedia of Genes and Genomes (KEGG) pathway analysis. Our investigation of the biological significance of common hDEGs and key genes utilized the Database for Annotation, Visualization, and Integrated Discovery (DAVID) web platform. This comprehensive analysis combined both GO terms and KEGG pathway information [34,35], with statistical significance determined using Fisher’s exact test and an adjusted *p*-value threshold of 0.05. This approach provided deeper insights into the functional roles and pathways associated with our identified genes.

### 2.5. Exploring hKGs-guided candidate drugs by molecular docking

We performed molecular docking analysis to evaluate potential therapeutic compounds for dengue fever, using both key gene proteins and their associated transcription factors as drug targets. We obtained three-dimensional receptor structures from multiple databases: Protein Data Bank [36], AlphaFold [37], and SWISS-MODEL [38], while potential drug molecules were sourced from PubChem [39]. Initially, we collected candidate ligands/agents from other literature for the DENVI inhibitor and the DGIdb database [40], which are significant and of approved status corresponding with hKGs and TFs (see S5 Table). Our analysis protocol involved several steps. Using Discovery Studio Visualizer [41] for structural examination, we prepared receptor proteins by eliminating water molecules and introducing charges using AutoDock tools [42,43]. We optimized therapeutic compounds’ energy using Avogadro software [44], followed by preprocessing with AutoDock tools. The final docking analysis was conducted using AutoDock Vina [45] to determine binding affinity scores (kcal/mol) between receptors and potential drug compounds. This comprehensive approach allowed us to evaluate the potential effectiveness of drug candidates through their molecular interactions with target proteins. Let the binding affinity score to be established between the *i*^th^ target protein (receptor), where (*i* = 1, 2, …, *m*), and the *j*^th^ drug agent be denoted by S_*ij,*_ where (*j* = 1, 2,..., *n*)_._ Subsequently, the target receptors were arranged by row sums $\sum_{j=\text{1}}^{n}{\text{S}}_{ij}=\text{1},\text{2},\;\ldots ,\;m$, and drug agents (ligands) by column sums to select the top-ranked compounds we apply $\sum_{i=\text{1}}^{m}{\text{S}}_{ij}$, *j* = 1, 2, …, n. Based on the binding affinity scores, we sorted and selected the top 30 drugs, which we illustrated. Subsequently, we applied drug-likeness criteria using Lipinski’s Rule of Five. Those compounds that did not meet the conditions were delisted, and we then selected the top 10 drugs for the ADME/T test. Finally, we recommend the top-ranked drug molecules.

#### 2.5.1. Screening of candidate drugs by Drug-likeness profile, ADME, and toxicity analysis.

The drug-likeness characteristics of the gathered molecular structure of the drug were assessed using Lipinski’s Rule of Five (Ro5) [46], which is a recognized method for evaluating the potential for oral absorption and the resemblance to lead compounds. To identify the drug-likeness attributes of specific ligands, we utilized online resources, including SwissADME [47]. We evaluated potential drug candidates through ADME/T analysis, examining their absorption, distribution, metabolism, excretion, and toxicity profiles. This crucial assessment helps predict both the safety and effectiveness of pharmaceutical compounds, particularly important in drug repurposing efforts where molecules may show promising target binding but require favorable pharmacokinetic properties. Our comprehensive analysis of top-ranked compounds focused on their drug-like properties and structural characteristics. We used pkCSM [48] online platforms that provided detailed ADME/T parameter predictions. Using SMILES format for molecular structures, we conducted thorough computational analyses to identify compounds with optimal pharmaceutical properties. This systematic approach ensures that only drug candidates with suitable pharmacokinetic profiles and safety characteristics advance in our therapeutic development process.

### 2.6. Density Functional Theory (DFT)

In order to explore the physicochemical properties of top-ranked selected drug molecules, we considered density functional theory (DFT) analysis. Using the Becke–3-parameter Lee–Yang–Parr (B3LYP) method with 6-311G [49–51] basis set in Gaussian 09 [45,46], we evaluated frontier molecular orbitals (FMO), electronic and structural characteristics, and thermodynamic properties in the gas phase for the proposed drug molecules. In DFT analysis, B3LYP is a widely used method that helps to model molecules accurately by combining key parts of electron behavior, and choosing the right basis set is essential to ensure that the results are reliable [52]. The study calculated various quantum chemical properties for the four top-scoring drug molecules using Gaussian 09 and GaussView-6. These included highest occupied molecular orbital (HOMO) and lowest unoccupied molecular orbital (LUMO) energies, energy gap (∆E), electron affinity, electrophilicity index, hardness, and softness parameters, providing detailed insights into their molecular characteristics [53]. A small HOMO-LUMO gap typically suggests that a molecule can easily undergo electronic transitions, enhancing its reactivity. Conversely, a large HOMO-LUMO gap indicates greater stability and less reactivity, which might be desirable in developing drugs that need to perform specific functions without unwanted side effects [54]. The mathematical definitions of these parameters are provided below [51]: ∆E = E_LUMO_ *–* E_HOMO,_ I = *-*E_HOMO,_ A = *-*E_LUMO,_ $\eta \;=\frac{\text{I}\;-\;\text{A}}{\text{2}}$, $\sigma \;=\;\frac{\text{1}}{\eta }$, $\chi \;=\;\frac{\text{I}\;+\;\text{A}}{\text{2}}$, µ = *-* χ, $\omega \;=\frac{x^{\text{2}}}{2\eta }$.

### 2.7. Molecular Dynamics (MD) Simulation Analysis

Molecular dynamics (MD) simulations utilizing YASARA software [55] (version 16.9) were conducted to examine the stability of the highest-ranked protein–ligand complexes. Each of these complexes underwent a 100 ns MD simulation under controlled conditions, which included a pH of 7.4, a temperature of 298 K, and a solvent density of 0.997 [56]. The AMBER14 force field [57] was employed with the standard simulation parameters. Throughout the simulation, the ligand within the complex experienced an acceleration control force of 1000 pm/ps2 (picometer/picosecond squared) [58]. The assessment was carried out by analyzing the backbone alpha carbon or side chain atom. We evaluated the root mean square deviation (RMSD), root mean square fluctuation (RMSF), and dynamic cross-correlation matrix (DCCM) of the protein–ligand complexes to gain a deeper understanding of structural deviations and fluctuations in dynamical conditions. This research highlights how the binding of a ligand to a protein’s active site affects its ability to achieve equilibrium [59]. The RMSD and RMSF values for each complex were plotted based on the following equations [60]: $\text{RMSD}\;=\;\sqrt{\frac{\sum_{i=\text{1}}^{N}R_{i}\;\times \;R_{i}}{n}}$, $\text{RMSF}\;=\;\sqrt{\sum_{j=\text{1}}^{\text{3}}\left(\frac{\text{1}}{N}\sum_{k=\text{1}}^{N}P_{ijk}^{\text{2}}-{\overset{―}{P}}_{ijk}^{\text{2}}\right)}$. where RMSD represents the root mean square deviation; *i* denotes the variables; and *R*_*i*_ signifies the vector that connects the positions of atom *i* (out of *N* atoms) in both the reference snapshot and the current snapshot following optimal superposition, and RMSF stands for root mean square fluctuation; the RMSF of atom *i* is calculated with *j* ranging from 1 to 3 for the *x*, *y*, and *z* coordinates of the atom position vector *P*, while *k* iterates over the set of *N* evaluated snapshots.

The molecular mechanics Poisson–Boltzmann surface area (MM-PBSA) method was employed to determine the binding free energy (BFE) of the chosen protein–ligand complexes. This was achieved by utilizing trajectory snapshots derived from the MD simulation results through YASARA [55]. The specified equation was applied to assess the BFE:


$$\text{BFE}=\;{\text{E}}_{\text{potRecept}}+{\text{E}}_{\text{solvRecept}}+{\text{E}}_{\text{potLigand}}+{\text{E}}_{\text{solvLigand}}-{\text{E}}_{\text{potComplex}}-{\text{E}}_{\text{solvCom}}$$

In the field of drug design, this method is employed to evaluate the strength of the interaction between a drug candidate and its target protein. Negative binding energies suggest a favorable binding or a stable interaction, whereas positive binding energies imply an unstable interaction or unfavorable binding.

## 3. Results

### 3.1. Identification of host differentially expressed genes (hDEGs)

**Fig 2:**
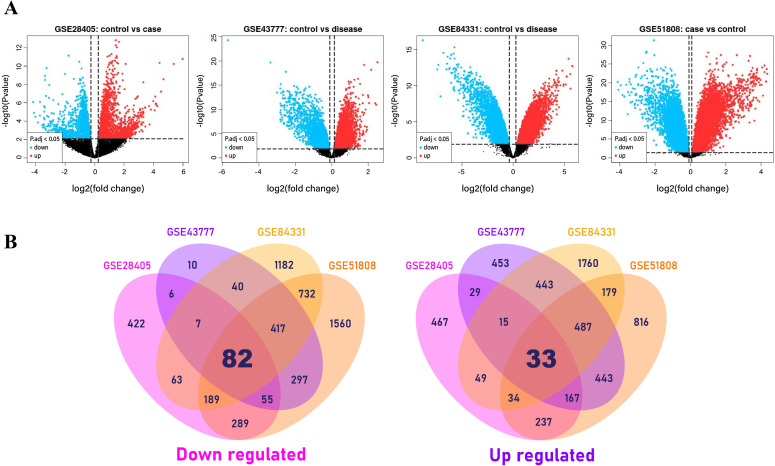
Volcano plots and venn diagrams for hDEGs.

From four datasets of microarray gene expression data, we detected a total of 115 (82 + 33) common hDEGs between DENVI and normal tissues (Fig 2B and S1 Table). From the datasets GSE81331, GSE51808, GSE28405, and GSE43777 (GPL570), the LIMMA approach by GEO2R has retrieved the top table for every dataset (**Fig 2A**). Then, based on the threshold values |log_2_FC| > 1 and adjusted *p*-value 0.05, we identified hDEGs respectively, for individual datasets (**Fig 2B**).

### 3.2. PPI network analysis of hDEGs for identification of hKGs

**Fig 3:**
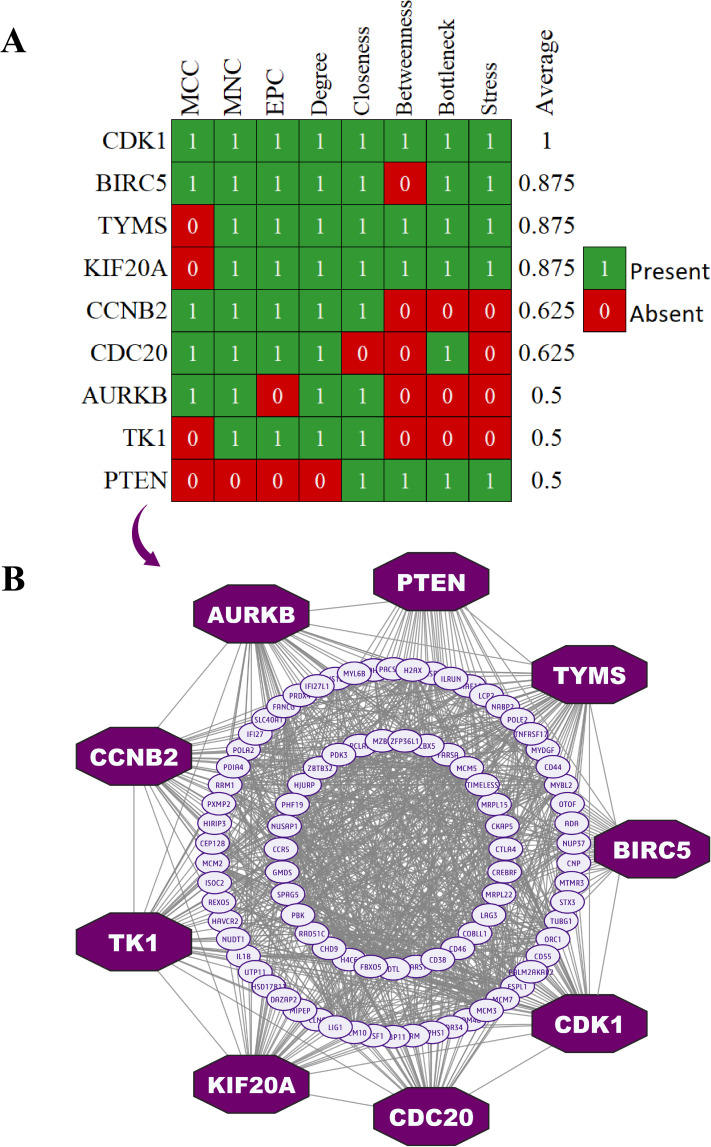
Selection of hKGs using eight topological measures in the PPI-network. (A) The top 9 hKGs were selected from the PPI network.(B) Protein-protein interactions (PPIs) network analysis of commonly host differentially expressed genes (hDEGs). Nodes depicted in an octagonal shape and colored purple represent the host key genes (hKGs).

Analysis of the DEG-based PPI network revealed a structure comprising 177 nodes connected by 2153 edges (**Fig 3B**). By evaluating multiple network parameters, including Degree, Maximal Clique Centrality (MCC), Betweenness, Closeness, MNC, Bottleneck, Stress, and Edge Percolated Component (EPC), we selected the top-ranked 9 hDEGs (CDK1, BIRC5, TYMS, KIF20A, CCNB2, CDC20, AURKB, TK1, and PTEN) as the host key-genes (hKGs) based on the higher frequency in the lists of top-ranked 10 DEGs selected for each of the topological measures for further analysis (Fig 3A and S2 Table).

#### 3.3.1. Regulatory network analysis of hKGs to detect key regulators.

**Fig 4:**
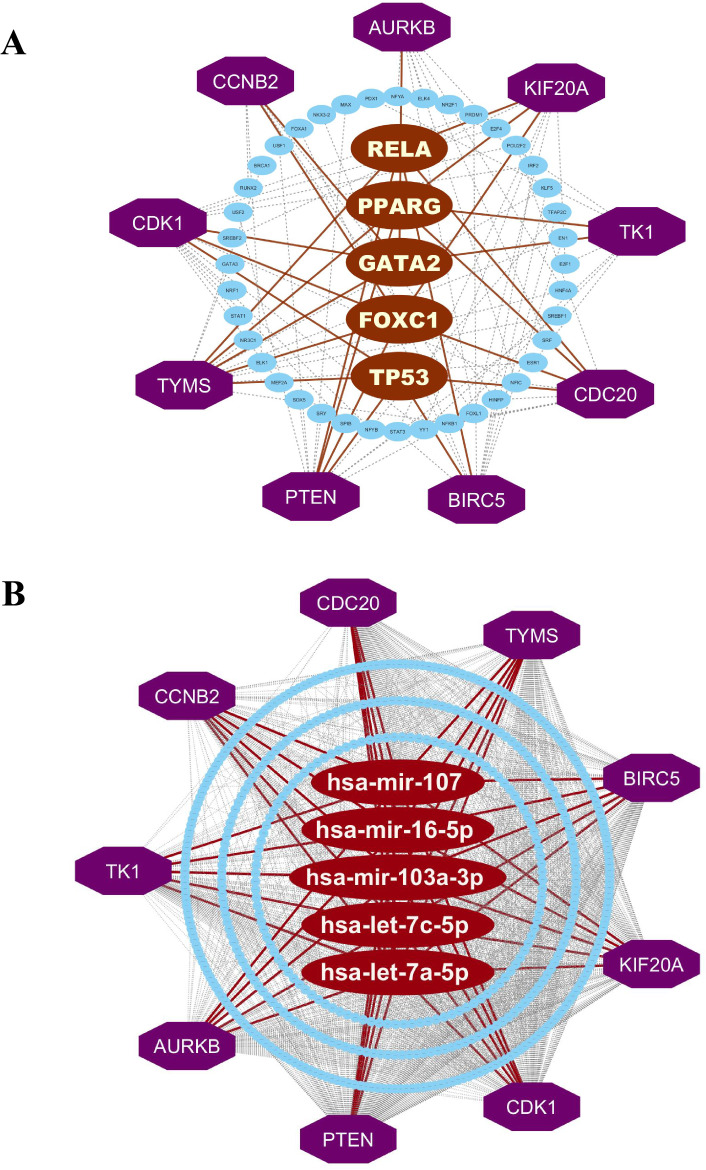
hKGs regulatory networks. (A) TFs-hKGs interaction network, (B) miRNAs-hKGs interaction network. Here, hKGs were marked as purple with octagon shapes. The TFs and miRNAs were marked in blue and brown colors with an ellipse shape.

We investigated TF and miRNA networks to identify regulatory elements controlling hKGs (S3 Table). Through analysis using degree (cutoff: 5) and betweenness (cutoff: 75) metrics, we identified five primary transcriptional factors: GATA2, RELA, TP53, PPARG, and FOXC1 (**Fig 4A**). Similar analysis with adjusted parameters (degree: 9, betweenness: 690.62) revealed five key post-transcriptional miRNA regulators: hsa-let-7a-5p, hsa-let-7c-5p, hsa-mir-16-5p, hsa-mir-103a-3p, and hsa-mir-107, which modulate KG expression (**Fig 4B**).

#### 3.3.2. GO and KEGG pathway enrichment analysis of hKGs.

We conducted GO and KEGG pathway enrichment analyses on nine key host genes to understand common mechanisms of dengue virus infection. **Table 2** shows the top five molecular functions, biological processes, cellular components, and KEGG pathways. These enriched pathways and GO terms involving host key genes significantly correlate with dengue infection pathogenesis.

**Table 2:** GO terms and KEGG pathways that are significantly enriched with common hDEGs by incorporating KGs related to the pathogenetic processes of DENVI.

Molecular Functions (MF)				
GO Id	GO Term	No. of hDEGs	Associated KGs	Adj. *P*-Value
GO:0005515	protein binding	147	CDK1, BIRC5, KIF20A, CCNB2, CDC20, AURKB, TK1, PTEN	3.07E-06
GO:0005524	ATP binding	33	CDK1, KIF20A, AURKB, TK1	5.72E-04
GO:0042802	identical protein binding	29	BIRC5, TK1, PTEN	0.072084885
GO:0010997	anaphase-promoting complex binding	3	CDC20, PTEN	0.096785479
GO:0004674	protein serine/threonine kinase activity	10	CDK1, AURKB	0.232664689
**Cellular Component (CC)**				
**GO Id**	**GO Term**	**No. of hDEGs**	**Associated KGs**	**Adj. *P*-Value**
GO:0005829	cytosol	84	CDK1, BIRC5, TYMS, CCNB2, CDC20, AURKB, TK1, PTEN	1.22E-06
GO:0005634	nucleus	72	CDK1, BIRC5, TYMS, KIF20A, CCNB2, AURKB, TK1, PTEN	0.025093646
GO:0005737	cytoplasm	74	CDK1, BIRC5, TYMS, KIF20A, CCNB2, TK1, PTEN	0.00113738
GO:0005654	nucleoplasm	63	CDK1, BIRC5, KIF20A, CDC20, AURKB, PTEN	2.75E-05
GO:0005876	spindle microtubule	5	CDK1, BIRC5, KIF20A, AURKB	0.014011111
**Biological Process (BP)**				
**GO Id**	**GO Term**	**No. of hDEGs**	**Associated KGs**	**Adj. *P*-Value**
GO:0051301	cell division	22	CDK1, BIRC5, CCNB2, CDC20, AURKB	1.96E-08
GO:0090307	mitotic spindle assembly	6	BIRC5, KIF20A, CDC20, AURKB	0.006226361
GO:0006915	apoptotic process	21	CDK1, BIRC5, PTEN	3.86E-05
GO:0000281	mitotic cytokinesis	6	BIRC5, KIF20A, AURKB	0.030502939
GO:0016310	phosphorylation	16	CDK1, AURKB, TK1	0.030502939
**KEGG Pathways**				
**KEGG Id**	**KEGG Term**	**No. of hDEGs**	**Associated KGs**	**Adj. *P*-Value**
hsa04110	Cell cycle	17	CDK1, CCNB2, CDC20, AURKB	7.43E-10
hsa04114	Oocyte meiosis	8	CDK1, CCNB2, CDC20	0.036730188
hsa04218	Cellular senescence	7	CDK1, CCNB2, PTEN	0.278592269
hsa05166	Human T-cell leukemia virus 1 infection	8	CCNB2, CDC20, PTEN	0.321554236
hsa04115	p53 signaling pathway	4	CDK1, CCNB2, PTEN	0.969059139

### 3.4. Exploring hKGs-guided candidate drugs by molecular docking

**Fig 5:**
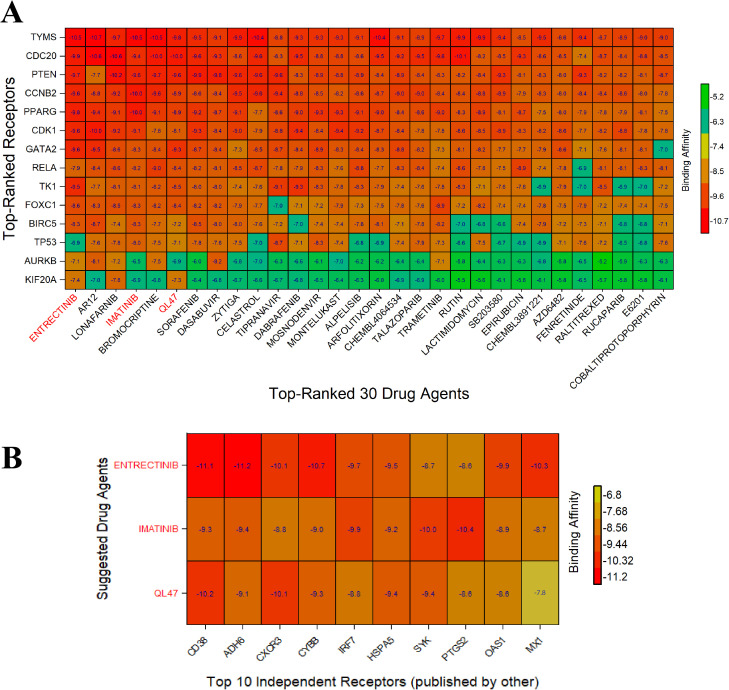
Image of drug-target binding affinity top 30 matrices (A) X-axis indicates top-ordered 30 drug agents (out of 183) and Y-axis indicates ordered proposed receptor proteins. The target receptor proteins are arranged in rows, and the drug agents are listed in columns, with red colors indicating strong binding affinities. The red text in the rows signifies the proposed drug agents (selected from ADME/T analysis). (B) Proposed drugs are represented on the Y-axis, while the X-axis displays top 10 (out of 35) target proteins identified in various publications as highlighted genes for DENVI.

**Fig 6:**
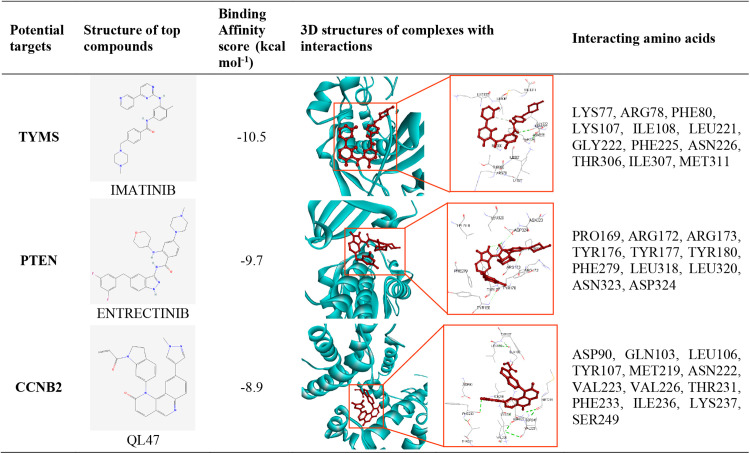
This figure presents the post-docking analysis and ligand-protein interactions: The first, second, and third columns illustrate potential targets, the 2D structures of lead compounds, and the top-ranked binding affinities (kcal mol^−^^1^), respectively. The fourth column presents a 3D view of the highest-ranking drug-target complexes. Last column emphasizes key aspects of the interacting amino acids.

In order to perform molecular docking analysis, we evaluate potential therapeutic compounds (see S5 Table) for dengue fever, using both key gene proteins and their associated transcription factors (see S4 Table). The matrix presents the leading 30 results from a total of 183 in the molecular docking analysis (Fig 5A and S6 Table). We suggested the three candidate drugs (red colored) molecules by ADMET chrematistics from HGBs-guided top-ranked drugs (ENTRECTINIB, IMATINIB, and QL47). Then we assessed the effectiveness of these three chemical molecules against 35 distinct independent receptors (see S8 Table), which validated our findings (Fig 5B and S9 Table). Also, we presented a post-docking analysis and some of the most binding ligand-protein interactions with the 3D view, and interacting amino acids (**Fig 6**).

#### 3.4.1. Screening of candidate drugs by Drug-likeness profile, ADME, and toxicity analysis.

Following molecular docking, the highest-ranked 30 drug molecules were evaluated for their drug-likeness using the online tools SwissADME. These molecules exhibited robust pharmacokinetic properties with few infringements of Lipinski’s Rule of Five. The compounds that fulfilled essential criteria were chosen (refer to S7 Table). The top 10 drugs were selected out of 11 (LONAFARNIB doesn’t meet Lipinski’s Rule of Five), and we conducted each compound’s ADME/T test. Proposed pharmaceuticals demonstrated the potential to traverse the neurological barrier, with Blood-Brain Barrier (BBB) metrics consistently under 0.3 (**Table 3**). The compounds are anticipated to partially penetrate neurological systems based on LogPS (CNS) measurements. Cytochrome P 450 (CYP) enzymes represent essential membrane-bound hemoproteins critical for metabolic regulation, pharmaceutical detoxification, and cellular processes. Approximately 80% of oxidative metabolism and nearly 50% of standard clinical pharmaceutical elimination involve multiple CYP enzymes from classes 1–3. The investigated medications exhibited characteristics enabling inhibition of the human CYP3A4 membrane mechanism. Toxicological evaluations utilizing AMES tests, lethal dose LD50, and aquatic organism toxicity LC50 demonstrated the compounds’ inertness across assessment criteria. Consequently, the substances are projected as non-toxic, displaying favorable pharmaceutical characteristics and potential for oral administration.

**Table 3:** ADME/T characteristics of the top-ranked ten drugs.

Compounds	Absorption			Distribution		Metabolism	Excretion	Toxicity		
	Caco2 Permeability	HIA (%)	P-gpI	BBB	CNS	CYP3A4 (Inhibitor)	TC	AMES	Minnow toxicity (log mM)	LD_50_ (mole/kg)
				(Permeability)						
**ENTRECTINIB**	0.607	87.259	Yes	−1.295	−2.293	Yes	0.829	No	2.884	2.376
AR-12	0.542	92.218	Yes	−0.785	−1.565	Yes	0.478	Yes	−0.389	2.474
**IMATINIB**	1.092	93.847	Yes	−1.376	−2.514	Yes	0.716	No	2.089	2.9
BROMOCRIPTINE	0.449	71.357	Yes	−0.711	−2.601	Yes	0.327	No	2.448	3.739
**QL47**	1.134	100	Yes	−0.892	−2.017	Yes	0.548	No	−0.253	2.405
SORAFENIB	0.762	85.494	Yes	−1.473	−2.025	Yes	−0.213	No	−0.515	2.14
DASABUVIR	−0.111	85.507	Yes	−1.03	−2.411	Yes	0.735	No	0.87	3.081
ABIRATERONE ACETATE	1.27	97.082	Yes	0.394	−2.742	Yes	0.423	No	−1.831	2.446
CELASTROL	0.464	100	No	0.078	−1.278	No	−0.094	No	−0.642	2.362
TIPRANAVIR	0.664	98.275	Yes	−1.368	−3.063	Yes	0.131	No	−2.023	2.367

### 3.5. Density Functional Theory (DFT)

**Fig 7:**
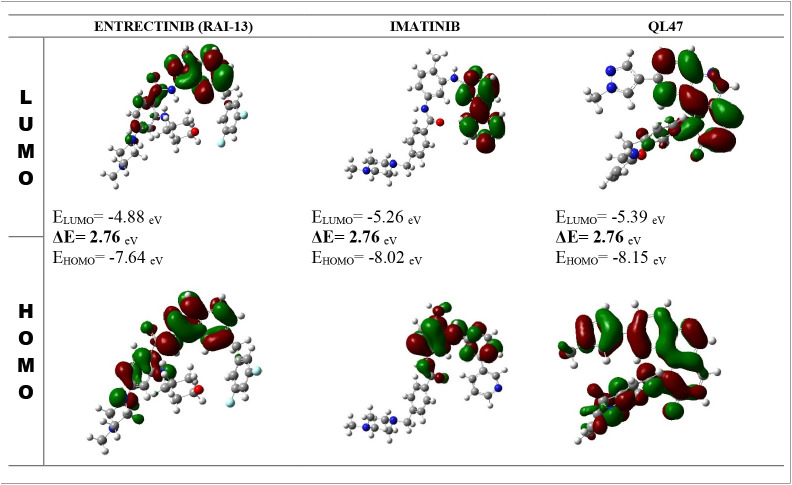
The molecular orbitals of the chosen candidate drug molecules, particularly the HOMO and LUMO.

Frontier molecular orbital analysis evaluates a compound’s reactivity through HOMO-LUMO relationships (**Fig 7**). The energy gap between HOMO and LUMO levels indicates chemical stability and reactivity – larger gaps suggest lower reactivity due to slower electronic transitions. This analysis helps understand ligand-receptor binding interactions, with HOMO-LUMO parameters providing crucial insights into molecular behavior in binding pockets [61,62].

Analysis of HOMO-LUMO energy gaps reveals that ENTRECTINIB (RAI-13), IMATINIB, and QL47 share around the same and lower energy gap (2.76 eV). The compounds show (**Fig 7**) HOMO energy levels between −7.64 eV and −8.15 eV, with LUMO levels ranging from −4.71 eV to −5.39 eV, where lower energy gaps indicate greater molecular stability [63,64].

The study evaluated (**Table 4**) quantum chemical properties of top-ranked phytocompounds using DFT analysis in Gaussian 09, examining HOMO-LUMO energies and various molecular parameters. In electron affinity analysis, QL47 showed the highest value (5.39 eV), followed by IMATINIB (5.26 eV), and ENTRECTINIB (4.88 eV). Higher electron affinity indicates greater ability to accept electrons, potentially affecting molecular interactions in biological systems. The compounds demonstrated notable hardness (η) and softness (σ) values, which determine molecular behavior in chemical processes. The high hardness and low softness values aligned with previous research findings. Electronegativity (χ) measurements revealed the compounds’ electron-attracting capabilities [65], while their electrophilicity index (ω) values exceeded those of conventional medicines, indicating stronger electrophilic properties [66,67].

**Table 4:** Identified FMO energies together with their corresponding physicochemical descriptors, which encompass chemical hardness, electronegativity, softness, chemical potential, and the global electrophilicity index.

Parameter	ENTRECTINIB	IMATINIB	QL47
LUMO energy (E_LUMO_)	−4.88	−5.26	−5.39
HOMO energy (E_HOMO_)	−7.64	−8.02	−8.15
Energy gap (∆E = E_LUMO_ *–* E_HOMO_)	2.76	2.76	2.76
Ionization potential (I)	7.64	8.02	8.15
Electron affinity (A)	4.88	5.26	5.39
Chemical hardness (η)	1.38	1.38	1.38
Softness (σ)	0.724	0.724	0.724
Electro-negativity (χ)	6.26	6.64	6.77
Chemical potential (µ)	−6.26	−6.64	−6.77
electrophilicity (ω)	14.198	15.974	16.606

Based on the molecular docking study, the three highest-ranked protein–ligand complexes were selected for additional molecular dynamics (MD) simulation analysis. MD simulation serves as a robust technique for investigating the dynamic behavior of molecular systems and is frequently employed in drug design to predict drug–target interactions. To effectively analyze the results of an MD simulation, several critical parameters (including RMSF, RMSD, and MM-PBSA) that offer insights into the stability and behavior of the molecular system need to be considered.

### 3.6. Molecular Dynamics (MD) Simulation Analysis

**Fig 8:**
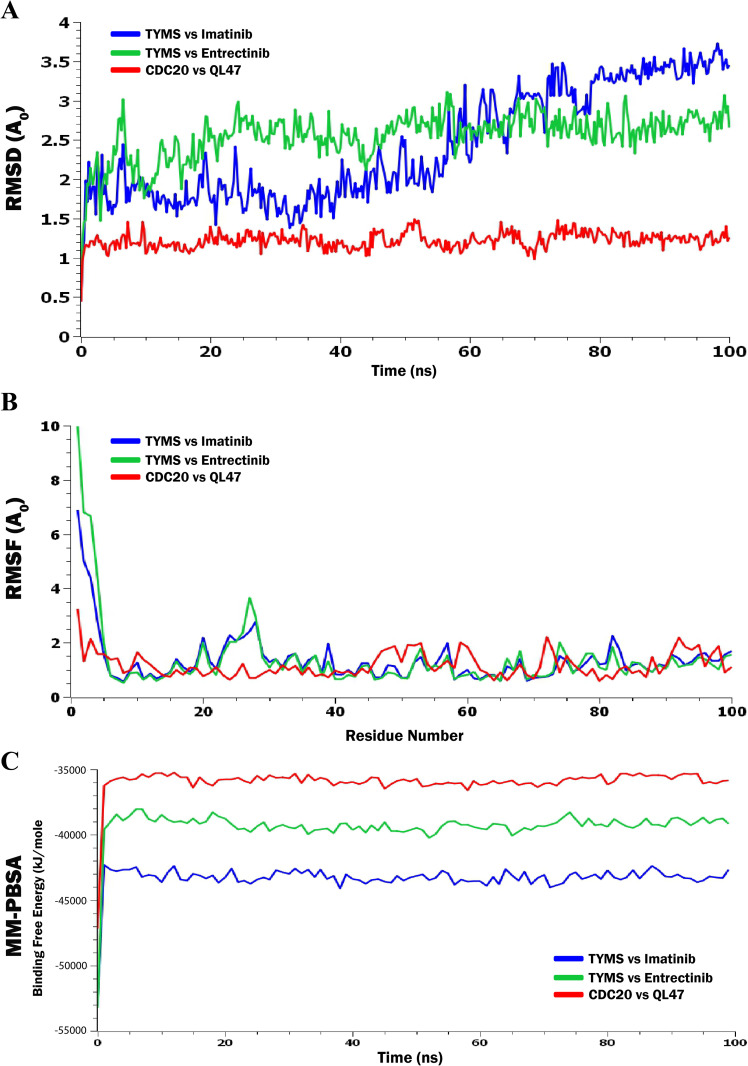
MD simulation results for top three complexes. (A) The results of the RMSD analysis for a 100-nanosecond simulation involving each of the proposed three drug-target complexes, (B) The RMSF is assessed based on the average RMSF of the atoms that make up the residue, and (C) calculations of binding free energy (MM-PBSA) for the proposed three drug-target complexes.

Throughout the 100-ns MD simulation, the configurational alterations of all protein–ligand complexes were assessed using root mean square deviation (RMSD) as a metric. The RMSD graph for all protein–ligand complexes is presented in **Fig 8A**. RMSD values are inherently nonnegative, with a value of zero indicating a perfect fit to the data, a condition that is frequently realized in practice. The RMSD values of the protein–ligand complexes were recorded to comprehend the structural deviations under dynamic conditions. It also clarifies how the ability of a protein to reach equilibrium is affected by its binding to the active site [68]. Based on **Fig 8A**, it was noted that the complexes of TYMS with Entrectinib and CDC20 with QL47 appear to be stable within the range of 1–3 A0, while the standard deviation from the backbone is approximately between 2.0 and 2.5 A0 [69] (S10 Table). However, in the case of TYMS vs Imatinib, the deviation exceeds 3.0 A0 after ~75 ns, which is viewed as somewhat stable in its location. Therefore, it can be inferred that the targeted ligands appear to maintain stability with the structure of the target protein.

In MD simulations, the RMSF serves as a crucial indicator of the flexibility and movement of protein-ligand complexes. **Fig 8B** depicts the individual RMSF calculations for the protein-ligand complexes involving the proposed three phytocompounds. A residue or atom that displays a lower RMSF value indicates greater rigidity, whereas a higher RMSF value signifies increased flexibility or mobility. The secondary conformations observed in the results remain consistent throughout the entire duration of the MD simulation. The average RMSF values recorded for the TYMS vs Imatinib, TYMS vs Entrectinib, and CDC20 vs QL47 complexes were 1.3519, 1.3882, and 1.1823 angstroms (A0), respectively (S10 Table).

To verify the binding affinity of the molecular docking energy associated with the ligand–protein complex, we conducted a BFE analysis utilizing MM-PBSA. The energy calculations for the complexes were derived from the 100 ns MD simulation trajectories through MM-PBSA methods, as illustrated in **Fig 8C**. The average BFE values for the complexes, namely TYMS vs Imatinib, TYMS vs Entrectinib, and CDC20 vs QL47, were −43302.4791, −39343.02385, and −35920.34507 kJ/mol, respectively (S10 Table). A greater negative value of MM-PBSA binding energy indicates a stronger affinity for the protein binding pocket [56,70]. Consequently, the experimental results indicated that the complexes exhibited negative MM-PBSA binding energies, signifying that these complexes established stable interactions with the main protease of DENV. Therefore, these compounds could serve as potential inhibitors of DENV infection.

## 4. Discussion

**Fig 9:**
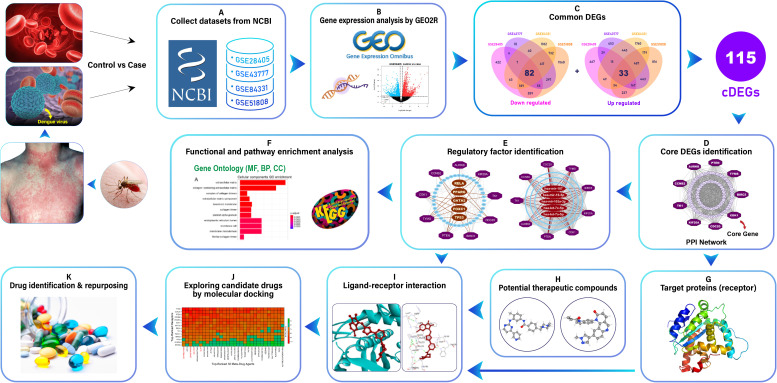
The pipeline of this study.

Dengue fever (DF) is a severe threat globally for human health. Despite its severity, research identifying host key genes driving dengue virus infection and potential treatments remains insufficient. Researchers have been using bioinformatics methods to discover potential biomarkers and understand dengue disease mechanisms, seeking to enhance diagnostic accuracy and develop effective treatment approaches [10–12]. That’s why we try to underscore the integrative in-silico analyses, and we demonstrated our full workflow in **Fig 9**. We investigated essential genomic markers in DENVI by analyzing four NCBI-GEO datasets (GSE81331, GSE51808, GSE28405, and GSE43777-GPL570), which revealed 115 common hDEGs between DENVI and control samples. While this number of hDEGs offers comprehensive insights, practical constraints of wet-lab experimentation, including time, cost, and labor intensity, necessitated identifying a more focused gene set. To address these limitations while maintaining research significance, we prioritized nine high-ranking hDEGs as core genes: CDK1, BIRC5, TYMS, KIF20A, CCNB2, CDC20, AURKB, TK1, and PTEN. This strategic reduction enables efficient laboratory investigation while preserving essential biological insights.

The gene CDK1, a key regulator of mitosis, shows reduced expression during dengue virus infection. This connects to other mosquito-transmitted ortho-flavivirus like Zika (ZIKV) and Japanese encephalitis virus (JEV), which can cause brain inflammation in humans [4,16,71]. Some studies found that the gene BIRC5 shows significant expression differences when comparing dengue shock syndrome (DSS) cases to healthy controls [13,72–74]. TYMS downregulation acts as a suppressor of host defense against viruses by diminishing DNA replication, cell division, and mitosis-related functions. This gene’s down-expression significantly impacts cellular metabolism, proliferation, and cell cycle progression [13,75]. Studies have shown that KIF20A, a critical gene, exhibits distinct expression patterns when comparing dengue hemorrhagic fever cases to healthy individuals [74,76]. CCNB2 stands out as one of the most statistically significant differentially expressed genes in the analysis. Various studies have discovered that patients developing severe dengue hemorrhagic fever (DHF) show reduced activity in pathways controlling cell growth and metabolism, compared to those with milder dengue symptoms. This distinct pattern involves decreased expression of key cell cycle regulators, including the cyclin CCNB2 and the cell division controller CDC20. Interestingly, patients with less severe dengue maintain higher activity levels of these essential growth-related genes [72,74,77,78]. We utilize from various literature, the selective inhibitor ZM 447439 has revealed that Aurora kinase B (AURKB) plays a significant role in dengue virus (DENV) biology. This enzyme appears to be particularly important during viral assembly and release phases of DENV’s life cycle, suggesting AURKB could be a potential therapeutic target for controlling viral replication and spread [13,72,74,76,79]. Recent experimental findings highlight thymidine kinase 1’s (TK1) pivotal role in cellular thymidine toxicity mechanisms. Through comprehensive genome-wide screening approaches, researchers observed that multiple sgRNAs (single-guide RNAs) targeting TK1 successfully provided resistance against thymidine’s toxic effects. The high frequency of effective sgRNAs in this screen strongly supports TK1’s critical function in mediating thymidine-induced cellular damage, demonstrating the enzyme’s fundamental role in this process [72,74,80]. We uncovered an unexpected function of the PTEN protein, traditionally known for suppressing tumors, in facilitating dengue virus (DENV) infection through its interaction with lipid droplets (LDs). This groundbreaking discovery demonstrates that when cells produce higher levels of PTEN, it promotes more efficient viral multiplication. This finding adds a new dimension to our understanding of PTEN’s cellular roles beyond its well-known tumor-suppressing functions, particularly in viral infections [74,81,82].

The six hKGs (CDK1, BIRC5, KIF20A, CCNB2, TK1, PTEN) were found to participate in protein binding and are mainly located in the nucleus and cytoplasm, indicating their involvement during dengue virus infection. Protein binding is fundamental in flavivirus infection mechanisms, mediating crucial viral-host protein interactions. This process is particularly important during infections with viruses like Dengue and Zika, where specific protein-binding events facilitate viral replication and spread. Similar patterns are observed in BTV (Bluetongue virus) infections, where enhanced protein binding activity [83]. During viral infection, flavivirus proteins interact with nuclear components to manipulate host gene expression and cellular responses. These nucleus-associated interactions influence viral replication success and immune evasion strategies, making the nuclear compartment a crucial target for understanding viral pathogenesis and developing antiviral treatments [84]. During infection, cytoplasmic interactions protect viral DNA from host detection, as seen with CypA binding to HIV-1 capsid. In USUV infections, cytoplasmic viral components disrupt MAVS-MDA5 interactions, suppressing interferon responses and reducing IFN-β production, thereby enabling the virus to evade the host’s immune response [85]. TYMS, CDC20, AURKB, and other hKGs follow the Cytosolic function, within the cytoplasm, host proteins detect viral genetic material and normally trigger protective interferon responses through pathways involving MAVS and STING. However, viruses actively interfere with these defense mechanisms by deploying proteins that block cellular sensors or conceal viral material. This strategic disruption of immune surveillance creates an environment where viruses can multiply more effectively while avoiding host detection [86–88]. Flaviviruses exploit cell division pathways to inhibit immune responses while promoting viral proliferation. Our five hKGs are associated with this function. Through interference with mitotic mechanisms, these viruses optimize their replication by hijacking cellular machinery and suppressing host defenses. Their manipulation of cell division regulators effectively converts host cells into viral factories [89,90]. We hypothesize that these functions may play a key role in causing the disease.

We utilize several research studies on transcriptional regulators that lead to the expression of our findings in hKGs. FOXC1, a FOX family transcription factor, regulates key processes like EMT, angiogenesis, metabolism, and cell survival. Its expression is finely tuned by transcriptional, post-transcriptional, post-translational, and epigenetic mechanisms [87]. FOXC1 is downregulated in ZIKV-infected neural cells, and it is involved in virus-induced neurodevelopmental defects. Given the similarity between ZIKV and DENV, FOXC1 suppression during DENV infection may contribute to vascular or neurological abnormalities, potentially linking its dysregulation to dengue pathogenesis through shared flaviviral mechanisms [91,92]. During DENVI, GATA-2 expression is significantly downregulated, impairing megakaryopoiesis and reducing platelet production. This downregulation correlates with the PI3K/AKT/mTOR pathway, which DENV may modulate to suppress GATA-2 transcription. As a result, reduced GATA-2 activity contributes to hematological abnormalities seen in dengue, such as thrombocytopenia and hemorrhagic manifestations [93]. During DENV infection, RELA (part of the NF-κB complex [94,95]) translocates to the nucleus following IκB degradation and collaborates with IRF3 to activate IFN-β transcription. This initiates an antiviral response via interferon-stimulated genes. RELA deficiency impairs IFN-β production, weakening the host defense against DENV replication [96]. PPARG regulates lipid metabolism and inflammatory responses in monocytes/macrophages. During DENV infection, altered PPARG expression affects cholesterol homeostasis, influencing viral replication and immune modulation. Its strong association with dengue severity suggests that PPARG dysregulation contributes to pathogenesis by shaping lipid-driven inflammatory responses in dengue-infected immune cells [97,98].

hsa-let-7a-5p is downregulated during dengue infection and is linked to cell death and innate immunity regulation, suggesting it may influence viral pathogenesis by modulating host inflammatory and antiviral responses [99]. hsa-let-7c-5p enhances dengue infection by targeting and downregulating BACH1, a transcription factor involved in antiviral defense, thereby facilitating increased viral replication in infected human hepatoma cells [100]. hsa-miR-16-5p is used as a reference control in gene expression analysis. It plays an indirect biological role in enhancing DENV infection [101]. Based on the article, miR-103a-3p promotes Zika virus replication by targeting OTUD4, a negative regulator of p38 MAPK signaling, thereby enhancing viral replication. Most probably similarly, it enhances DENV replication by downregulating OTUD4 and activating the p38 MAPK pathway, a mechanism known to be exploited by several flaviviruses to support viral propagation [102].

Finally, hKGs-guided top-ranked three drug agents (ENTRECTINIB, IMATINIB, and QL47) were suggested against DENVI by molecular docking (**Fig 5A**), ADME/T (**Table 3**), and DFT (**Table 4**) analysis. Then the effectiveness of these molecules was assessed against the top-ranked 10 (out of 35) distinct independent receptors identified as DENVI-causing hKGs in the previous independent studies, by molecular docking analysis (**Fig 5B**). Binding affinity scores showed Entrectinib (RAI-13) has emerged as a powerful, broad-spectrum antiviral agent, particularly effective against DENV. Research shows it directly targets and inhibits the viral RNA-dependent RNA polymerase (RdRp) in both DENV and human norovirus (hNV), with demonstrated IC50 values of 2.43 μM for DENV2 and 2.01 μM for murine norovirus [103]. Research has now revealed that this small molecule compound also effectively inhibits multiple flaviviruses, comprising the dengue virus (DENV), Zika virus (ZIKV), and the Japanese encephalitis virus (JEV), suggesting its potential broader therapeutic applications [104–106]. Imatinib shows promise against DENV by inhibiting c-ABL kinase, which the virus exploits during infection. It reverses DENV-2-induced endothelial cell changes, restores junctional proteins, suppresses mesenchymal markers, and blocks viral entry and infection stages. This suggests potential for mitigating severe dengue pathogenesis [107,108]. QL47 is a promising small-molecule antiviral agent targeting host mechanisms. It inhibits viral translation and BTK kinase, effectively disrupting the infectious cycle of positive-strand RNA viruses like DENV. By blocking viral RNA translation at non-toxic concentrations, QL47 shows broad-spectrum antiviral potential with a favorable therapeutic index [109–111].

## 5. Conclusion

This study identified dengue virus infection (DENVI) causing nine hKGs (CDK1, BIRC5, TYMS, KIF20A, CCNB2, CDC20, AURKB, TK1, and PTEN), highlighting their pathogenetic processes and therapeutic drugs through the comprehensive bioinformatics analysis. The hKGs-set enrichment analysis with gene ontology (GO) terms and KEGG-pathways revealed some crucial biological processes (i.g, cell division, apoptotic process), molecular functions (protein binding, ATP binding), cellular components (cytosol, nucleus, cytoplasm, nucleoplasm), and signaling pathways (Cell cycle, Oocyte meiosis), that might be associated with the development of DENVI. The hKGs-regulatory networks analysis disclosed some crucial transcriptional and post-transcriptional regulators of hKGs that are also associated with the development of DENVI. Finally, hKGs-guided three drug molecules (ENTRECTINIB, IMATINIB, and QL47) were suggested against DENVI by molecular docking analysis. We also observed that these molecules significantly bind to the top-ranked independent receptors suggested by previous studies. The ADME/T and DFT analyses showed good pharmacokinetics and physicochemical properties of these drug molecules, respectively. Thus, the findings of this study might be valuable resources for the diagnosis and treatment of DENVI.

## Supportivng information

S1 Fig UMAP plot for GSE51808. (PNG)

S1 Table List of upregulated and downregulated common hDEGs of DENVI. (DOCX)

S2 Table List of key genes (KGs) from the PPI network based on different topological measures. (DOCX)

S3 Table Gene Ontology and KEGG pathway analysis in KGs. (DOCX)

S4 Table Collect protein structures from the database for molecular docking. (DOCX)

S5 Table Collection of DENVI-related candidate drugs from published articles and online web tools (DGIdb). (DOCX)

S6 Table Binding affinity score within drugs and corresponding proteins and TFs. (DOCX)

S7 Table Drug-likeness profile of top 30 drugs. (DOCX)

S8 Table Collection of DENVI-related highlighted genes from publications by others. (DOCX)

S9 Table Binding affinity score within suggested drugs and suggested protein (published by others, causing DENVI). (DOCX)

S10 Table Scores of Molecular Dynamics (MD) Simulations. (DOCX)

## References

[1] Yap SSL, Nguyen-Khuong T, Rudd PM, Alonso S. Dengue virus glycosylation: what do we know? Front Microbiol. 2017;8. doi: 10.3389/fmicb.2017.01415 PMC5524768 28791003

[2] Sinha S, Singh K, Ravi Kumar YS, Roy R, Phadnis S, Meena V, et al. Dengue virus pathogenesis and host molecular machineries. J Biomed Sci. 2024;31(1):43. doi: 10.1186/s12929-024-01030-9 38649998 PMC11036733

[3] Villordo SM, Filomatori CV, Sánchez-Vargas I, Blair CD, Gamarnik AV. Dengue virus RNA structure specialization facilitates host adaptation. PLoS Pathog. 2015;11(1):e1004604. doi: 10.1371/journal.ppat.1004604 25635835 PMC4311971

[4] Xie S, Yang X, Yang X, Cao Z, Wei N, Lin X, et al. Japanese Encephalitis Virus NS1 and NS1’ Proteins Induce Vimentin Rearrangement via the CDK1-PLK1 Axis to Promote Viral Replication. 2024. 10.1128/jvi.00195-24 PMC11092344 38656209

[5] Roy SK, Bhattacharjee S. Dengue virus: epidemiology, biology, and disease aetiology. Can J Microbiol. 2021;67(10):687–702. doi: 10.1139/cjm-2020-0572 34171205

[6] Salazar MI, del Angel RM, Lanz-Mendoza H, Ludert JE, Pando-Robles V. The role of cell proteins in dengue virus infection. J Proteomics. 2014;111:6–15. doi: 10.1016/j.jprot.2014.06.002 24930603

[7] Seema, Jain SK. Molecular mechanism of pathogenesis of dengue virus: Entry and fusion with target cell. Indian J Clin Biochem. 2005;20(2):92–103. doi: 10.1007/BF02867407 23105540 PMC3453834

[8] Uno N, Ross TM. Dengue virus and the host innate immune response. Emerg Microbes Infect. 2018;7(1):167. doi: 10.1038/s41426-018-0168-0 30301880 PMC6177401

[9] Chauhan N, Gaur KK, Asuru TR, Guchhait P. Dengue virus: pathogenesis and potential for small molecule inhibitors. Biosci Rep. 2024;44(8):BSR20240134. doi: 10.1042/BSR20240134 39051974 PMC11327219

[10] Khetarpal N, Khanna I. Dengue fever: causes, complications, and vaccine strategies. J Immunol Res. 2016;2016:6803098. doi: 10.1155/2016/6803098 27525287 PMC4971387

[11] Screaton G, Mongkolsapaya J, Yacoub S, Roberts C. New insights into the immunopathology and control of dengue virus infection. Nat Rev Immunol. 2015;15(12):745–59. doi: 10.1038/nri3916 26603900

[12] Brady OJ, Gething PW, Bhatt S, Messina JP, Brownstein JS, Hoen AG, et al. Refining the global spatial limits of dengue virus transmission by evidence-based consensus. PLoS Negl Trop Dis. 2012;6(8):e1760. doi: 10.1371/journal.pntd.0001760 PMC3413714 22880140

[13] Josyula JVN, Talari P, Pillai AKB, Mutheneni SR. Analysis of gene expression profile for identification of novel gene signatures during dengue infection. Infect Med (Beijing). 2023;2(1):19–30. doi: 10.1016/j.imj.2023.02.002 38076406 PMC10699721

[14] Xiong N, Sun Q. Identification of stage-related and severity-related biomarkers and exploration of immune landscape for Dengue by comprehensive analyses. Virol J. 2022;19(1):130. doi: 10.1186/s12985-022-01853-8 35918744 PMC9344228

[15] Xie L-M, Yin X, Bi J, Luo H-M, Cao X-J, Ma Y-W, et al. Identification of potential biomarkers in dengue via integrated bioinformatic analysis. PLoS Negl Trop Dis. 2021;15(8):e0009633. doi: 10.1371/journal.pntd.0009633 34347790 PMC8336846

[16] Paul JK, Azmal M, Alam T, Talukder OF, Ghosh A. Comprehensive analysis of intervention and control studies for the computational identification of dengue biomarker genes. PLoS Negl Trop Dis. 2025;19(3):e0012914. doi: 10.1371/journal.pntd.0012914 PMC11918421 40100920

[17] Barrett T, Suzek TO, Troup DB, Wilhite SE, Ngau W-C, Ledoux P, et al. NCBI GEO: mining millions of expression profiles--database and tools. Nucleic Acids Res. 2005;33:D562–6. doi: 10.1093/nar/gki022 15608262 PMC539976

[18] Smyth GK. Linear models and empirical bayes methods for assessing differential expression in microarray experiments. Stat Appl Genet Mol Biol. 2004;3:Article3. doi: 10.2202/1544-6115.1027 16646809

[19] Kalyan GU, Pranathi Reddy D, Chandrakanth G, Pooja B, Anitha V, Vivek D. Gene Association Disease Prediction by GEO2R Tool. In: 2023 International Conference on Evolutionary Algorithms and Soft Computing Techniques (EASCT). 2023. pp. 1–6. doi: 10.1109/easct59475.2023.10392802

[20] Ritchie ME, Phipson B, Wu D, Hu Y, Law CW, Shi W, et al. limma powers differential expression analyses for RNA-sequencing and microarray studies. Nucleic Acids Res. 2015;43(7):e47. doi: 10.1093/nar/gkv007 25605792 PMC4402510

[21] Chandele A, Sewatanon J, Gunisetty S, Singla M, Onlamoon N, Akondy RS, et al. Characterization of human CD8 T cell responses in dengue virus-infected patients from India. J Virol. 2016;90(24):11259–78. doi: 10.1128/JVI.01424-16 27707928 PMC5126381

[22] Jones S, Thornton JM. Principles of protein-protein interactions. Proc Natl Acad Sci U S A. 1996;93(1):13–20. doi: 10.1073/pnas.93.1.13 8552589 PMC40170

[23] Ben-Hur A, Noble WS. Kernel methods for predicting protein-protein interactions. Bioinformatics. 2005;21 Suppl 1:i38–46. doi: 10.1093/bioinformatics/bti1016 15961482

[24] Szklarczyk D, Gable AL, Lyon D, Junge A, Wyder S, Huerta-Cepas J, et al. STRING v11: protein-protein association networks with increased coverage, supporting functional discovery in genome-wide experimental datasets. Nucleic Acids Res. 2019;47(D1):D607–13. doi: 10.1093/nar/gky1131 30476243 PMC6323986

[25] Szklarczyk D, Franceschini A, Kuhn M, Simonovic M, Roth A, Minguez P, et al. The STRING database in 2011: functional interaction networks of proteins, globally integrated and scored. Nucleic Acids Res. 2011;39:D561–8. doi: 10.1093/nar/gkq973 21045058 PMC3013807

[26] Chin C-H, Chen S-H, Wu H-H, Ho C-W, Ko M-T, Lin C-Y. cytoHubba: identifying hub objects and sub-networks from complex interactome. BMC Syst Biol. 2014;8 Suppl 4(Suppl 4):S11. doi: 10.1186/1752-0509-8-S4-S11 25521941 PMC4290687

[27] Ciriello G, Gallina C, Guerra C. Analysis of interactions between ribosomal proteins and RNA structural motifs. BMC Bioinformatics. 2010;11 Suppl 1(Suppl 1):S41. doi: 10.1186/1471-2105-11-S1-S41 20122215 PMC3009514

[28] Khan A, Fornes O, Stigliani A, Gheorghe M, Castro-Mondragon JA, van der Lee R, et al. JASPAR 2018: update of the open-access database of transcription factor binding profiles and its web framework. Nucleic Acids Res. 2018;46(D1):D1284. doi: 10.1093/nar/gkx1188 29161433 PMC5753202

[29] Sethupathy P, Corda B, Hatzigeorgiou AG. TarBase: A comprehensive database of experimentally supported animal microRNA targets. RNA. 2006;12(2):192–7. doi: 10.1261/rna.2239606 16373484 PMC1370898

[30] Xia J, Benner MJ, Hancock REW. NetworkAnalyst--integrative approaches for protein-protein interaction network analysis and visual exploration. Nucleic Acids Res. 2014;42:W167–74. doi: 10.1093/nar/gku443 24861621 PMC4086107

[31] Shannon P, Markiel A, Ozier O, Baliga NS, Wang JT, Ramage D, et al. Cytoscape: A Software Environment for Integrated Models. Genome Res. 1971;13:426. doi: 10.1101/gr.1239303.metabolite PMC403769 14597658

[32] Harris MA, Clark J, Ireland A, Lomax J, Ashburner M, Foulger R, et al. The Gene Ontology (GO) database and informatics resource. Nucleic Acids Res. 2004;32:D258–61. doi: 10.1093/nar/gkh036 14681407 PMC308770

[33] Doms A, Schroeder M. GoPubMed: exploring PubMed with the gene ontology. Nucleic Acids Res. 2005;33:W783–6. doi: 10.1093/nar/gki470 15980585 PMC1160231

[34] Kanehisa M, Araki M, Goto S, Hattori M, Hirakawa M, Itoh M, et al. KEGG for linking genomes to life and the environment. Nucleic Acids Res. 2008;36:D480–4. doi: 10.1093/nar/gkm882 18077471 PMC2238879

[35] Kanehisa M, Goto S. KEGG: kyoto encyclopedia of genes and genomes. Nucleic Acids Res. 2000;28(1):27–30. doi: 10.1093/nar/28.1.27 10592173 PMC102409

[36] Berman HM, Westbrook J, Feng Z, Gilliland G, Bhat TN, Weissig H, et al. The Protein Data Bank. Nucleic Acids Res. 2000;28(1):235–42. doi: 10.1093/nar/28.1.235 10592235 PMC102472

[37] Varadi M, Anyango S, Deshpande M, Nair S, Natassia C, Yordanova G, et al. AlphaFold Protein Structure Database: massively expanding the structural coverage of protein-sequence space with high-accuracy models. Nucleic Acids Res. 2022;50(D1):D439–44. doi: 10.1093/nar/gkab1061 34791371 PMC8728224

[38] Schwede T, Kopp J, Guex N, Peitsch MC. SWISS-MODEL: An automated protein homology-modeling server. Nucleic Acids Res. 2003;31(13):3381–5. doi: 10.1093/nar/gkg520 12824332 PMC168927

[39] Kim S, Chen J, Cheng T, Gindulyte A, He J, He S, et al. PubChem 2019 update: improved access to chemical data. Nucleic Acids Res. 2019;47(D1):D1102–9. doi: 10.1093/nar/gky1033 30371825 PMC6324075

[40] Cannon M, Stevenson J, Stahl K, Basu R, Coffman A, Kiwala S, et al. DGIdb 5.0: rebuilding the drug-gene interaction database for precision medicine and drug discovery platforms. Nucleic Acids Res. 2024;52(D1):D1227–35. doi: 10.1093/nar/gkad1040 37953380 PMC10767982

[41] Shaweta S, Akhil S, Utsav G. Molecular Docking studies on the Anti-fungal activity of Allium sativum (Garlic) against Mucormycosis (black fungus) by BIOVIA discovery studio visualizer 21.1.0.0. Ann Antivir Antiretrovir. 2021:028–32. doi: 10.17352/aaa.000013

[42] Ahmmed R, Hossen MB, Ajadee A, Mahmud S, Ali MA, Mollah MMH, et al. Bioinformatics analysis to disclose shared molecular mechanisms between type-2 diabetes and clear-cell renal-cell carcinoma, and therapeutic indications. Sci Rep. 2024;14(1):19133. doi: 10.1038/s41598-024-69302-w 39160196 PMC11333728

[43] Morris GM, Huey R, Lindstrom W, Sanner MF, Belew RK, Goodsell DS, et al. AutoDock4 and AutoDockTools4: automated docking with selective receptor flexibility. J Comput Chem. 2009;30(16):2785–91. doi: 10.1002/jcc.21256 19399780 PMC2760638

[44] Hanwell MD, Curtis DE, Lonie DC, Vandermeersch T, Zurek E, Hutchison GR. Avogadro: an advanced semantic chemical editor, visualization, and analysis platform. J Cheminform. 2012;4(1). doi: 10.1186/1758-2946-4-17 PMC3542060 22889332

[45] Trott O, Olson AJ. AutoDock Vina: improving the speed and accuracy of docking with a new scoring function, efficient optimization, and multithreading. J Comput Chem. 2010;31(2):455–61. doi: 10.1002/jcc.21334 19499576 PMC3041641

[46] Lipinski CA. Lead- and drug-like compounds: the rule-of-five revolution. 2004. 10.1016/j.ddtec.2004.11.007 24981612

[47] Daina A, Michielin O, Zoete V. SwissADME: a free web tool to evaluate pharmacokinetics, drug-likeness and medicinal chemistry friendliness of small molecules. Sci Rep. 2017;7:42717. doi: 10.1038/srep42717 28256516 PMC5335600

[48] Pires DEV, Blundell TL, Ascher DB. pkCSM: Predicting Small-Molecule Pharmacokinetic and Toxicity Properties Using Graph-Based Signatures. J Med Chem. 2015;58(9):4066–72. doi: 10.1021/acs.jmedchem.5b00104 25860834 PMC4434528

[49] Hertwig RH, Koch W. On the parameterization of the local correlation functional. What is Becke-3-LYP? Chem Phys Lett. 1997;268(5–6):345–51. doi: 10.1016/s0009-2614(97)00207-8

[50] Tariq A, Nazir S, Arshad AW, Nawaz F, Ayub K, Iqbal J. DFT study of the therapeutic potential of phosphorene as a new drug-delivery system to treat cancer. RSC Adv. 2019;9(42):24325–32. doi: 10.1039/c9ra02778e 35527876 PMC9069575

[51] El-Shamy NT, Alkaoud AM, Hussein RK, Ibrahim MA, Alhamzani AG, Abou-Krisha MM. DFT, ADMET and Molecular Docking Investigations for the Antimicrobial Activity of 6,6’-Diamino-1,1’,3,3’-tetramethyl-5,5’-(4-chlorobenzylidene)bis[pyrimidine-2,4(1H,3H)-dione]. Molecules. 2022;27(3):620. doi: 10.3390/molecules27030620 35163880 PMC8839838

[52] Praveen PA, Saravanapriya D, Bhat SV, Arulkannan K, Kanagasekaran T. Comprehensive analysis of DFT-3C methods with B3LYP and experimental data to model optoelectronic properties of tetracene. Mater Sci Semicond Process. 2024;173:108159. doi: 10.1016/j.mssp.2024.108159

[53] Dennington R, Keith T, Millam J. Gauss View, Version 5. Shawnee Mission: Semichem Inc.; 2009.

[54] Hadigheh Rezvan V. Molecular structure, HOMO–LUMO, and NLO studies of some quinoxaline 1,4-dioxide derivatives: Computational (HF and DFT) analysis. Result Chem. 2024;7:101437. doi: 10.1016/j.rechem.2024.101437

[55] Krieger E, Koraimann G, Vriend G. Increasing the precision of comparative models with YASARA NOVA—a self‐parameterizing force field. Proteins. 2002;47(3):393–402. doi: 10.1002/prot.10104 11948792

[56] Hosen SMZ, Rubayed M, Dash R, Junaid M, Mitra S, Alam MS, et al. Prospecting and Structural Insight into the Binding of Novel Plant-Derived Molecules of Leea indica as Inhibitors of BACE1. Curr Pharm Des. 2018;24(33):3972–9. doi: 10.2174/1381612824666181106111020 30398111

[57] Case DA, Cheatham TE 3rd, Darden T, Gohlke H, Luo R, Merz KM Jr, et al. The Amber biomolecular simulation programs. J Comput Chem. 2005;26(16):1668–88. doi: 10.1002/jcc.20290 16200636 PMC1989667

[58] Gupta S, Biswas A, Akhter MS, Krettler C, Reinhart C, Dodt J, et al. Revisiting the mechanism of coagulation factor XIII activation and regulation from a structure/functional perspective. Sci Rep. 2016;6:30105. doi: 10.1038/srep30105 27453290 PMC4958977

[59] Ahmad S, Abbasi HW, Shahid S, Gul S, Abbasi SW. Molecular docking, simulation and MM-PBSA studies of nigella sativa compounds: a computational quest to identify potential natural antiviral for COVID-19 treatment. J Biomol Struct Dyn. 2021;39(12):4225–33. doi: 10.1080/07391102.2020.1775129 32462996 PMC7298883

[60] Land H, Humble MS. YASARA: A Tool to Obtain Structural Guidance in Biocatalytic Investigations. In: Bornscheuer UT, Höhne M, editors. Protein Engineering: Methods and Protocols. New York, NY: Springer New York; 2018. pp. 43–67. 10.1007/978-1-4939-7366-8_4 29086303

[61] Tsuneda T, Song J-W, Suzuki S, Hirao K. On Koopmans’ theorem in density functional theory. J Chem Phys. 2010;133(17):174101. doi: 10.1063/1.3491272 21054000

[62] Rahman MA, Chakma U, Kumer A, Rahman MR, Matin MM. Uridine-Derived 4-Aminophenyl 1-Thioglucosides: DFT Optimized FMO, ADME, and Antiviral Activities Study. Biointerface Res Appl Chem. 2022;13(1):52. doi: 10.33263/briac131.052

[63] Becke AD. Thermochemistry. III. The Role of Exact Exchange. J Chem Phys. 1993;98:5648–52.

[64] Mahmud S, Afrose S, Biswas S, Nagata A, Paul GK, Mita MA, et al. Plant-derived compounds effectively inhibit the main protease of SARS-CoV-2: An in silico approach. PLoS One. 2022;17(8):e0273341. doi: 10.1371/journal.pone.0273341 35998194 PMC9398018

[65] Karton A, Spackman PR. Evaluation of density functional theory for a large and diverse set of organic and inorganic equilibrium structures. J Comput Chem. 2021;42(22):1590–601. doi: 10.1002/jcc.26698 34121198

[66] Szymański S, Majerz I. Theoretical Studies on the Structure and Intramolecular Interactions of Fagopyrins-Natural Photosensitizers of Fagopyrum. Molecules. 2022;27(12):3689. doi: 10.3390/molecules27123689 35744813 PMC9230917

[67] Srivastava R. Theoretical studies on the molecular properties, toxicity, and biological efficacy of 21 new chemical entities. ACS Omega. 2021;6(38):24891–901. doi: 10.1021/acsomega.1c03736 34604670 PMC8482469

[68] Kufareva I, Abagyan R. Methods of protein structure comparison. In: Orry AJW, Abagyan R, editors. Homology modeling: methods and protocols. Totowa, NJ: Humana Press; 2012. pp. 231–57. 10.1007/978-1-61779-588-6_10 PMC4321859 22323224

[69] Tran Q-H, Nguyen Q-T, Vo N-Q-H, Mai TT, Tran T-T-N, Tran T-D, et al. Structure-based 3D-Pharmacophore modeling to discover novel interleukin 6 inhibitors: An in silico screening, molecular dynamics simulations and binding free energy calculations. PLoS One. 2022;17(4):e0266632. doi: 10.1371/journal.pone.0266632 35385549 PMC8986010

[70] Qureshi R, Ghosh A, Yan H. Correlated Motions and Dynamics in Different Domains of Epidermal Growth Factor Receptor With L858R and T790M Mutations. IEEE/ACM Trans Comput Biol Bioinform. 2022;19(1):383–94. doi: 10.1109/TCBB.2020.2995569 32750848

[71] Chatel-Chaix L, Cortese M, Romero-Brey I, Bender S, Neufeldt CJ, Fischl W, et al. Dengue Virus Perturbs Mitochondrial Morphodynamics to Dampen Innate Immune Responses. Cell Host Microbe. 2016;20(3):342–56. doi: 10.1016/j.chom.2016.07.008 27545046 PMC7105029

[72] Long HT, Hibberd ML, Hien TT, Dung NM, Van Ngoc T, Farrar J, et al. Patterns of gene transcript abundance in the blood of children with severe or uncomplicated dengue highlight differences in disease evolution and host response to dengue virus infection. J Infect Dis. 2009;199(4):537–46. doi: 10.1086/596507 19138155 PMC4333209

[73] Winter C, Camarão AAR, Steffen I, Jung K. Network meta-analysis of transcriptome expression changes in different manifestations of dengue virus infection. BMC Genomics. 2022;23(1):165. doi: 10.1186/s12864-022-08390-2 35220956 PMC8882220

[74] Murarik MR. Identification of biomarkers for the prediction of dengue disease severity using high-throughput proteomics. 2023.

[75] Waickman AT, Victor K, Li T, Hatch K, Rutvisuttinunt W, Medin C, et al. Dissecting the heterogeneity of DENV vaccine-elicited cellular immunity using single-cell RNA sequencing and metabolic profiling. Nat Communi. pp. 1–16. 10.1038/s41467-019-11634-7 PMC6694189 31413301

[76] Chiu H-C, Hannemann H, Heesom KJ, Matthews DA, Davidson AD. High-throughput quantitative proteomic analysis of dengue virus type 2 infected A549 cells. PLoS One. 2014;9(3):e93305. doi: 10.1371/journal.pone.0093305 24671231 PMC3966871

[77] Fung TK, Poon RYC. A roller coaster ride with the mitotic cyclins. Semin Cell Dev Biol. 2005;16(3):335–42. doi: 10.1016/j.semcdb.2005.02.014 15840442

[78] Ioannidis LJ, Studniberg SI, Eriksson EM, Suwarto S, Denis D, Liao Y, et al. Integrated systems immunology approach identifies impaired effector T cell memory responses as a feature of progression to severe dengue fever. J Biomed Sci. 2023;30(1):24. doi: 10.1186/s12929-023-00916-4 37055751 PMC10103532

[79] Olais JHP, Jimenez FR, García EJC, Rivas RH, Angel RMD. AurKB activity is necessary for Dengue virus release. Access Microbiol. 2019;1(10). doi: 10.1099/acmi.imav2019.po0046

[80] Puschnik AS, Majzoub K, Ooi YS, Carette JE. A CRISPR toolbox to study virus-host interactions. Nat Rev Microbiol. 2017;15(6):351–64. doi: 10.1038/nrmicro.2017.29 28420884 PMC5800792

[81] Liu B, Gao T-T, Fu X-Y, Xu Z-H, Ren H, Zhao P, et al. PTEN Lipid Phosphatase Activity Enhances Dengue Virus Production through Akt/FoxO1/Maf1 Signaling. Virol Sin. 2021;36(3):412–23. doi: 10.1007/s12250-020-00291-6 33044659 PMC8257821

[82] Williams GH, Stoeber K. The cell cycle and cancer. J Pathol. 2012;226(2):352–64. doi: 10.1002/path.3022 21990031

[83] Gebhart NN, Hardy RW, Sokoloski KJ. Comparative analyses of alphaviral RNA:Protein complexes reveals conserved host-pathogen interactions. PLoS One. 2020;15(8):e0238254. doi: 10.1371/journal.pone.0238254 32841293 PMC7446964

[84] Tripathi A, Chauhan S, Khasa R. A Comprehensive review of the development and therapeutic use of antivirals in flavivirus infection. Viruses. 2025;17(1):74. doi: 10.3390/v17010074 39861863 PMC11769230

[85] Nelemans T, Tas A, Kikkert M, van Hemert MJ. Usutu virus NS4A suppresses the host interferon response by disrupting MAVS signaling. Virus Res. 2024;347:199431. doi: 10.1016/j.virusres.2024.199431 38969013 PMC11292556

[86] Li S, Cao L, Zhang Z, Kuang M, Chen L, Zhao Y, et al. Cytosolic and nuclear recognition of virus and viral evasion. Mol Biomed. 2021;2(1):30. doi: 10.1186/s43556-021-00046-z 35006471 PMC8607372

[87] Anwar S, Ul Islam K, Azmi MI, Iqbal J. cGAS-STING-mediated sensing pathways in DNA and RNA virus infections: crosstalk with other sensing pathways. Arch Virol. 2021;166(12):3255–68. doi: 10.1007/s00705-021-05211-x 34622360

[88] Nainu F, Shiratsuchi A, Nakanishi Y. Induction of apoptosis and subsequent phagocytosis of virus-infected cells as an antiviral mechanism. Front Immunol. 2017;8:1220. doi: 10.3389/fimmu.2017.01220 29033939 PMC5624992

[89] Moni MA, Lio’ P. Genetic Profiling and Comorbidities of Zika Infection. J Infect Dis. 2017;216(6):703–12. doi: 10.1093/infdis/jix327 28934431

[90] Pong LY, Parkkinen S, Dhanoa A, Gan HM, Wickremesinghe IAC, Syed Hassan S. MicroRNA profiling of mouse liver in response to DENV-1 infection by deep sequencing. PeerJ. 2019;7:e6697. doi: 10.7717/peerj.6697 31065454 PMC6482938

[91] Biyani S, Patil AS, Swami V. The Influence of FOXC1 Gene on Development, Organogenesis, and Functions. Clin Transl Metab. 2024;22(1). doi: 10.1007/s12018-024-09297-0

[92] B YY, B WL, B HY, B YZ, B SZ, B FX, et al. Research progress on the regulatory mechanisms of FOXC1 expression in cancers and its role in drug resistance. 2018. 10.1016/j.gene.2023.148079 38101711

[93] Lahon A, Arya RP, Banerjea AC. Dengue virus dysregulates master transcription factors and PI3K/AKT/mTOR signaling pathway in megakaryocytes. Front Cell Infect Microbiol. 2021;11:715208. doi: 10.3389/fcimb.2021.715208 34513730 PMC8427595

[94] Ngo KA, Kishimoto K, Davis-Turak J, Pimplaskar A, Cheng Z, Spreafico R, et al. Dissecting the Regulatory Strategies of NF-κB RelA Target Genes in the Inflammatory Response Reveals Differential Transactivation Logics. Cell Rep. 2020;30(8):2758–2775.e6. doi: 10.1016/j.celrep.2020.01.108 32101750 PMC7061728

[95] Tian B, Widen SG, Yang J, Wood TG, Kudlicki A, Zhao Y, et al. The NFκB subunit RELA is a master transcriptional regulator of the committed epithelial-mesenchymal transition in airway epithelial cells. J Biol Chem. 2018;293(42):16528–45. doi: 10.1074/jbc.RA118.003662 30166344 PMC6200927

[96] Bais SS, Ratra Y, Khan NA, Pandey R, Kushawaha PK, Tomar S, et al. Chandipura Virus Utilizes the Prosurvival Function of RelA NF-κB for Its Propagation. J Virol. 2019;93(14):e00081–19. doi: 10.1128/JVI.00081-19 31043529 PMC6600208

[97] Devignot S, Sapet C, Duong V, Bergon A, Rihet P, Ong S, et al. Genome-wide expression profiling deciphers host responses altered during dengue shock syndrome and reveals the role of innate immunity in severe dengue. PLoS One. 2010;5(7):e11671. doi: 10.1371/journal.pone.0011671 20652028 PMC2907396

[98] Datta D, Ghosh S. Analyzing the Molecular Signature Genes and Pathways of Dengue Fever, Dengue Hemorrhagic Fever and Dengue Shock Syndrome Caused by Dengue Virus in India. Mol Biotechnol. 2025. doi: 10.1007/s12033-025-01407-7 39987330

[99] Lee NH, Lee E, Kim YS, Kim WK, Lee YK, Kim SH. Differential expression of microRNAs in the saliva of patients with aggressive periodontitis: a pilot study of potential biomarkers for aggressive periodontitis. J Periodontal Implant Sci. 2020;50(5):281–90. doi: 10.5051/jpis.2000120006 33124206 PMC7606899

[100] Zhou B, Chu M, Xu S, Chen X, Liu Y, Wang Z, et al. Hsa-let-7c-5p augments enterovirus 71 replication through viral subversion of cell signaling in rhabdomyosarcoma cells. Cell Biosci. 2017;7:7. doi: 10.1186/s13578-017-0135-9 28101327 PMC5237547

[101] Hapugaswatta H, Amarasena P, Premaratna R, Seneviratne KN, Jayathilaka N. Differential expression of microRNA, miR-150 and enhancer of zeste homolog 2 (EZH2) in peripheral blood cells as early prognostic markers of severe forms of dengue. J Biomed Sci. 2020;27(1):25. doi: 10.1186/s12929-020-0620-z 31954402 PMC6969970

[102] Ye H, Kang L, Yan X, Li S, Huang Y, Mu R, et al. MiR-103a-3p Promotes Zika Virus Replication by Targeting OTU Deubiquitinase 4 to Activate p38 Mitogen-Activated Protein Kinase Signaling Pathway. Front Microbiol. 2022;13:862580. doi: 10.3389/fmicb.2022.862580 35317262 PMC8934420

[103] Yi D, Li Q, Pang L, Wang Y, Zhang Y, Duan Z, et al. Identification of a Broad-Spectrum Viral Inhibitor Targeting a Novel Allosteric Site in the RNA-Dependent RNA Polymerases of Dengue Virus and Norovirus. Front Microbiol. 2020;11:1440. doi: 10.3389/fmicb.2020.01440 32670253 PMC7330483

[104] Zhang Z-R, Zhang H-Q, Li X-D, Deng C-L, Wang Z, Li J-Q, et al. Generation and characterization of Japanese encephalitis virus expressing GFP reporter gene for high throughput drug screening. Antiviral Res. 2020;182:104884. doi: 10.1016/j.antiviral.2020.104884 32750466 PMC7395821

[105] Joe S, Salam AAA, Neogi U, N NB, Mudgal PP. Antiviral drug research for Japanese encephalitis: an updated review. Pharmacol Rep. 2022;74(2):273–96. doi: 10.1007/s43440-022-00355-2 35182390 PMC8964565

[106] Zhu Y, Chen S, Lurong Q, Qi Z. Recent Advances in Antivirals for Japanese Encephalitis Virus. Viruses. 2023;15(5):1033. doi: 10.3390/v15051033 37243122 PMC10222399

[107] Escudero-fl M, Torres-hoyos D, Miranda-brand Y, Boudreau RL, Carlos J, Vicente-manzanares M. Dengue virus infection alters inter-endothelial junctions and promotes endothelial–mesenchymal-transition-like changes in human microvascular endothelial cells. 2023. 10.3390/v15071437 PMC10386726 37515125

[108] Schor S, Einav S. Repurposing of kinase inhibitors as broad-spectrum antiviral drugs. DNA Cell Biol. 2018;37(2):63–9. doi: 10.1089/dna.2017.4033 29148875 PMC5804095

[109] Liang Y, de Wispelaere M, Carocci M, Liu Q, Wang J, Yang PL, et al. Structure-Activity Relationship Study of QL47: A Broad-Spectrum Antiviral Agent. ACS Med Chem Lett. 2017;8(3):344–9. doi: 10.1021/acsmedchemlett.7b00008 28337328 PMC5346993

[110] de Wispelaere M, Carocci M, Liang Y, Liu Q, Sun E, Vetter ML, et al. Discovery of host-targeted covalent inhibitors of dengue virus. Antiviral Res. 2017;139:171–9. doi: 10.1016/j.antiviral.2016.12.017 28034743 PMC5373925

[111] de Wispelaere M, Carocci M, Burri DJ, Neidermyer WJ Jr, Olson CM, Roggenbach I, et al. A broad-spectrum antiviral molecule, QL47, selectively inhibits eukaryotic translation. J Biol Chem. 2020;295(6):1694–703. doi: 10.1074/jbc.RA119.011132 31914414 PMC7008383

