# Supplementary material for: In-silico identification of host-key-genes associated with dengue-virus-infections highlighting their pathogenetic mechanisms and therapeutic agents
Source: PLoS One. 2025 Oct 7;20(10):e0333509. doi: 10.1371/journal.pone.0333509 (PMC12503274; doi:10.1371/journal.pone.0333509)
Supplement: S1 Table — (DOCX) [file pone.0333509.s002.docx]

**S1 Table.** List of upregulated and downregulated common hDEGs of DENVI.

| **Downregulated (82)** | **Upregulated (33)** |
| --- | --- |
| LAG3,IFI27,IFI27L1,CHST12,HIRIP3,ADA,MYBL2,CCR5,MZB1,CCNB2,LIG1,MYL6B,FARSA,FBXO5,MRPL15,PHF19,TNFRSF17,GTSF1,CBX5,PBK,MRPL22,PACSIN1,MCM10,FKBP11,COBLL1,GMDS,POLE2,TK1,CDC20,SPAG5,MIS18A,NABP2,VARS,ARPC5L,CLN6,CKAP5,MCM2,ORC1,POLA2,IDH2,DTL,MCM3,PDIA4,GSG2,MCM7,H2AFX,WDR34,UTP11,PXMP2,AURKB,NUDT1,HAVCR2,TUBG1,SRM,CTLA4,CD38,RRM1,CHD9,OTOF,ESPL1,ZBTB32,ISOC2,CEP128,MT1F,KIF20A,HJURP,MYDGF,TIMELESS,RAD51C,PRDX4,FANCG,SEPHS1,MIPEP,CNP,MCM5,LOC81691,CHAF1A,NUSAP1,NUP37,TYMS,CDK1,BIRC5. | PDK3,STX3,ZFP36L1,CD55,HSD17B11,IL1B,LCP2,CPQ,KDM4B,SLC40A1,DAZAP2,CREBRF,CD46,CD44,MTMR3,PTEN,LTB,SLC25A37,PKN2,PELI2,SH3BGRL2,ITPKB,MAP3K2,JADE1,FBXO9,GP5,IVNS1ABP,SULF2,MYEF2,PRPF4B,IGF1R,CCNT2,HEMGN. |
