# Supplementary material for: In-silico identification of host-key-genes associated with dengue-virus-infections highlighting their pathogenetic mechanisms and therapeutic agents
Source: PLoS One. 2025 Oct 7;20(10):e0333509. doi: 10.1371/journal.pone.0333509 (PMC12503274; doi:10.1371/journal.pone.0333509)
Supplement: S2 Table — (DOCX) [file pone.0333509.s003.docx]

**S2 Table.** List of key genes (KGs) from the PPI network based on different topological measures.

| **MCC** | **MNC** | **EPC** | **Degree** | **Closeness** | **Betweenness** | **Bottleneck** | **Stress** |
| --- | --- | --- | --- | --- | --- | --- | --- |
| CDC45 | **CDK1** | **CDK1** | **CDK1** | **CDK1** | RNASEH2A | **CDK1** | RNASEH2A |
| **CDK1** | MCM5 | **TYMS** | MCM5 | **AURKB** | **CDK1** | PAICS | **CDK1** |
| PBK | **AURKB** | **TK1** | **AURKB** | **PTEN** | IL1B | SPAG5 | IL1B |
| SPAG5 | **TYMS** | **BIRC5** | **TYMS** | **TYMS** | CD44 | **PTEN** | CD44 |
| **AURKB** | **TK1** | **KIF20A** | **TK1** | **TK1** | **PTEN** | **TYMS** | **PTEN** |
| **BIRC5** | **BIRC5** | MYBL2 | **BIRC5** | MCM3 | **TYMS** | H4C6 | **TYMS** |
| NUSAP1 | **KIF20A** | MCM7 | **KIF20A** | **BIRC5** | MCM3 | **BIRC5** | H4C6 |
| TPX2 | MCM7 | **CCNB2** | MCM7 | **KIF20A** | H4C6 | **KIF20A** | **BIRC5** |
| **CCNB2** | **CCNB2** | CDC25A | **CCNB2** | **CCNB2** | **KIF20A** | CDC25A | **KIF20A** |
| **CDC20** | **CDC20** | **CDC20** | **CDC20** | CDC25A | TPX2 | **CDC20** | CTLA4 |
| **Bold indicates the selected key genes (KGs), were,**  **CDK1:** selected by all 8 topological measures  **BIRC5**: selected by 7 measures  **TYMS**: selected by 7 topological measures  **KIF20A**: selected by 7 topological measures  **CCNB2**: selected by 5 topological measures  **CDC20**: selected by 5 topological measures  **AURKB**: selected by 4 topological measures  **TK1**: selected by 4 topological measures  **PTEN**: selected by 4 topological measures | | | | | | | |
