# Supplementary material for: In-silico identification of host-key-genes associated with dengue-virus-infections highlighting their pathogenetic mechanisms and therapeutic agents
Source: PLoS One. 2025 Oct 7;20(10):e0333509. doi: 10.1371/journal.pone.0333509 (PMC12503274; doi:10.1371/journal.pone.0333509)
Supplement: S3 Table — (DOCX) [file pone.0333509.s004.docx]

**S3 Table.** Gene Ontology and KEGG pathway analysis in KGs.

| **Molecular Functions (MF)** | | | | | | | | | | | |
| --- | --- | --- | --- | --- | --- | --- | --- | --- | --- | --- | --- |
| **Term** | **Count** | **%** | **P-Value** | **List Total** | **Pop Hits** | **Pop Total** | **Fold Enrichment** | **Bonferroni** | **Benjamini** | **FDR** | **Associated KGs** |
| GO:0005515 protein binding | 147 | 84 | 8.56122E-09 | 172 | 12754 | 19304 | 1.29356955 | 3.07347E-06 | 3.07348E-06 | 3.01355E-06 | CDK1, BIRC5, KIF20A, CCNB2, CDC20, AURKB, TK1, PTEN |
| GO:0005524 ATP binding | 33 | 18.85714286 | 4.77609E-06 | 172 | 1541 | 19304 | 2.403422725 | 0.001713153 | 0.000571539 | 0.000560395 | CDK1, KIF20A, AURKB, TK1 |
| GO:0042802 identical protein binding | 29 | 16.57142857 | 0.001378743 | 172 | 1737 | 19304 | 1.873773279 | 0.39061812 | 0.072084885 | 0.07067933 | BIRC5, TK1, PTEN |
| GO:0010997 anaphase-promoting complex binding | 3 | 1.714285714 | 0.002695974 | 172 | 9 | 19304 | 37.41085271 | 0.620599079 | 0.096785479 | 0.094898297 | CDC20, PTEN |
| GO:0004674 protein serine/threonine kinase activity | 10 | 5.714285714 | 0.007129002 | 172 | 382 | 19304 | 2.938025082 | 0.923347867 | 0.232664689 | 0.228128052 | CDK1, AURKB |
| GO:0008017 microtubule binding | 8 | 4.571428571 | 0.011886538 | 172 | 277 | 19304 | 3.24137352 | 0.986333823 | 0.355605587 | 0.348671773 | BIRC5, KIF20A |
| GO:0106310 protein serine kinase activity | 9 | 5.142857143 | 0.016526517 | 172 | 366 | 19304 | 2.759817003 | 0.997477722 | 0.456386136 | 0.447487242 | CDK1, AURKB |
| GO:0042803 protein homodimerization activity | 13 | 7.428571429 | 0.035093344 | 172 | 750 | 19304 | 1.945364341 | 0.999997307 | 0.839900689 | 0.823523795 | BIRC5, TYMS |
| GO:1990757 ubiquitin ligase activator activity | 2 | 1.142857143 | 0.085152775 | 172 | 10 | 19304 | 22.44651163 | 1 | 1 | 0.983240223 | CDC20, PTEN |
| GO:0016887 ATP hydrolysis activity | 12 | 6.857142857 | 0.001730229 | 172 | 434 | 19304 | 3.103204373 | 0.462964077 | 0.077644038 | 0.076130087 | KIF20A |
| GO:0001618 virus receptor activity | 4 | 2.285714286 | 0.034163358 | 172 | 80 | 19304 | 5.611627907 | 0.999996194 | 0.839900689 | 0.823523795 | CDK1 |
| GO:0004721 phosphoprotein phosphatase activity | 3 | 1.714285714 | 0.042435879 | 172 | 37 | 19304 | 9.099937146 | 0.999999827 | 0.952155045 | 0.933589348 | PTEN |
| GO:0046872 metal ion binding | 34 | 19.42857143 | 0.053096334 | 172 | 2812 | 19304 | 1.357008171 | 0.999999997 | 1 | 0.983240223 | BIRC5 |
| GO:0004725 protein tyrosine phosphatase activity | 4 | 2.285714286 | 0.057853886 | 172 | 99 | 19304 | 4.534648814 | 0.999999999 | 1 | 0.983240223 | PTEN |
| GO:0045296 cadherin binding | 7 | 4 | 0.077951167 | 172 | 335 | 19304 | 2.345157931 | 1 | 1 | 0.983240223 | CCNB2 |
| GO:0019901 protein kinase binding | 9 | 5.142857143 | 0.078678846 | 172 | 502 | 19304 | 2.012137497 | 1 | 1 | 0.983240223 | KIF20A |
| GO:0017116 single-stranded DNA helicase activity | 6 | 3.428571429 | 9.33176E-07 | 172 | 21 | 19304 | 32.06644518 | 0.000334954 | 0.000167505 | 0.000164239 |  |
| GO:0003688 DNA replication origin binding | 5 | 2.857142857 | 9.9565E-06 | 172 | 16 | 19304 | 35.07267442 | 0.003568021 | 0.000893596 | 0.000876172 |  |
| GO:0003697 single-stranded DNA binding | 8 | 4.571428571 | 6.28013E-05 | 172 | 112 | 19304 | 8.016611296 | 0.022294114 | 0.004509135 | 0.004421213 |  |
| GO:0003677 DNA binding | 22 | 12.57142857 | 0.001405555 | 172 | 1157 | 19304 | 2.134067657 | 0.396463732 | 0.072084885 | 0.07067933 |  |
| GO:0005381 iron ion transmembrane transporter activity | 3 | 1.714285714 | 0.002109128 | 172 | 8 | 19304 | 42.0872093 | 0.531386405 | 0.084130792 | 0.082490358 |  |
| GO:0003678 DNA helicase activity | 3 | 1.714285714 | 0.046678964 | 172 | 39 | 19304 | 8.633273703 | 0.999999965 | 0.985749886 | 0.966529136 |  |
| GO:0015093 ferrous iron transmembrane transporter activity | 2 | 1.142857143 | 0.051992968 | 172 | 6 | 19304 | 37.41085271 | 0.999999995 | 1 | 0.983240223 |  |
| GO:0005388 P-type calcium transporter activity | 2 | 1.142857143 | 0.076972537 | 172 | 9 | 19304 | 24.94056848 | 1 | 1 | 0.983240223 |  |
| GO:0060090 molecular adaptor activity | 5 | 2.857142857 | 0.087540039 | 172 | 189 | 19304 | 2.969115295 | 1 | 1 | 0.983240223 |  |
| **Cellular Component (CC)** | | | | | | | | | | | |
| **Term** | **Count** | **%** | **P-Value** | **List Total** | **Pop Hits** | **Pop Total** | **Fold Enrichment** | **Bonferroni** | **Benjamini** | **FDR** | **Associated KGs** |
| GO:0005829 cytosol | 84 | 48 | 4.29358E-09 | 175 | 5649 | 20887 | 1.774784918 | 1.21508E-06 | 1.21508E-06 | 1.15497E-06 | CDK1, BIRC5, TYMS, CCNB2, CDC20, AURKB, TK1, PTEN |
| GO:0005634 nucleus | 72 | 41.14285714 | 0.000975371 | 175 | 6175 | 20887 | 1.391661307 | 0.241312119 | 0.025093646 | 0.023852264 | CDK1, BIRC5, TYMS, KIF20A, CCNB2, AURKB, TK1, PTEN |
| GO:0005737 cytoplasm | 74 | 42.28571429 | 2.81331E-05 | 175 | 5754 | 20887 | 1.534969959 | 0.007930158 | 0.00113738 | 0.001081113 | CDK1, BIRC5, TYMS, KIF20A, CCNB2, TK1, PTEN |
| GO:0005654 nucleoplasm | 63 | 36 | 2.91859E-07 | 175 | 4030 | 20887 | 1.865836228 | 8.25927E-05 | 2.7532E-05 | 2.617E-05 | CDK1, BIRC5, KIF20A, CDC20, AURKB, PTEN |
| GO:0005876 spindle microtubule | 5 | 2.857142857 | 0.000445583 | 175 | 43 | 20887 | 13.87840532 | 0.118498081 | 0.014011111 | 0.013317982 | CDK1, BIRC5, KIF20A, AURKB |
| GO:0030496 midbody | 8 | 4.571428571 | 0.00115983 | 175 | 192 | 20887 | 4.973095238 | 0.279941205 | 0.02735266 | 0.025999525 | CDK1, BIRC5, KIF20A, AURKB |
| GO:0005819 spindle | 7 | 4 | 0.001806469 | 175 | 153 | 20887 | 5.460653595 | 0.400520199 | 0.031951918 | 0.030371257 | BIRC5, KIF20A, CDC20, AURKB |
| GO:0000776 kinetochore | 10 | 5.714285714 | 1.14019E-05 | 175 | 167 | 20887 | 7.146963216 | 0.003221562 | 0.000645349 | 0.000613423 | BIRC5, CDC20, AURKB |
| GO:0005739 mitochondrion | 26 | 14.85714286 | 0.000597516 | 175 | 1485 | 20887 | 2.089704666 | 0.155615752 | 0.016909708 | 0.016073185 | CDK1, TYMS, TK1 |
| GO:0005813 centrosome | 15 | 8.571428571 | 0.001515913 | 175 | 671 | 20887 | 2.668128593 | 0.349054177 | 0.028600224 | 0.027185372 | CDK1, CCNB2, CDC20 |
| GO:0015630 microtubule cytoskeleton | 6 | 3.428571429 | 0.027255249 | 175 | 202 | 20887 | 3.545176803 | 0.999598498 | 0.350601616 | 0.333257366 | BIRC5, CCNB2, AURKB |
| GO:0005759 mitochondrial matrix | 8 | 4.571428571 | 0.063073872 | 175 | 421 | 20887 | 2.26801493 | 0.99999999 | 0.615513996 | 0.58506454 | CDK1, TYMS, TK1 |
| GO:0072686 mitotic spindle | 9 | 5.142857143 | 3.45875E-05 | 175 | 148 | 20887 | 7.258030888 | 0.009740687 | 0.001223534 | 0.001163005 | CDK1, KIF20A |
| GO:0000307 cyclin-dependent protein kinase holoenzyme complex | 3 | 1.714285714 | 0.051964504 | 175 | 44 | 20887 | 8.137792208 | 0.999999724 | 0.574939378 | 0.546497147 | CDK1, CCNB2 |
| GO:0005874 microtubule | 7 | 4 | 0.055796901 | 175 | 326 | 20887 | 2.562822086 | 0.999999912 | 0.574939378 | 0.546497147 | BIRC5, KIF20A |
| GO:0032133 chromosome passenger complex | 2 | 1.142857143 | 0.056884461 | 175 | 7 | 20887 | 34.10122449 | 0.999999937 | 0.574939378 | 0.546497147 | BIRC5, AURKB |
| GO:0000781 chromosome, telomeric region | 10 | 5.714285714 | 1.5128E-05 | 175 | 173 | 20887 | 6.89909166 | 0.004272105 | 0.000713537 | 0.000678239 | CDK1 |
| GO:0000922 spindle pole | 7 | 4 | 0.001423133 | 175 | 146 | 20887 | 5.722465753 | 0.331710367 | 0.028600224 | 0.027185372 | CDC20 |
| GO:0042995 cell projection | 7 | 4 | 0.005798185 | 175 | 194 | 20887 | 4.306597938 | 0.807115159 | 0.096522729 | 0.091747753 | PTEN |
| GO:0035749 myelin sheath adaxonal region | 2 | 1.142857143 | 0.048959504 | 175 | 6 | 20887 | 39.7847619 | 0.999999323 | 0.574939378 | 0.546497147 | PTEN |
| GO:0000775 chromosome, centromeric region | 3 | 1.714285714 | 0.091832306 | 175 | 61 | 20887 | 5.869882904 | 1 | 0.74252979 | 0.705796868 | BIRC5 |
| GO:0071162 CMG complex | 6 | 3.428571429 | 1.6804E-08 | 175 | 11 | 20887 | 65.10233766 | 4.75551E-06 | 2.37776E-06 | 2.26013E-06 |  |
| GO:0042555 MCM complex | 5 | 2.857142857 | 1.46692E-06 | 175 | 11 | 20887 | 54.25194805 | 0.000415051 | 0.000103784 | 9.86501E-05 |  |
| GO:0005694 chromosome | 9 | 5.142857143 | 0.001342734 | 175 | 254 | 20887 | 4.229088864 | 0.316308922 | 0.028600224 | 0.027185372 |  |
| GO:0000123 histone acetyltransferase complex | 3 | 1.714285714 | 0.016886372 | 175 | 24 | 20887 | 14.91928571 | 0.991930406 | 0.265491297 | 0.252357452 |  |
| GO:0035371 microtubule plus-end | 3 | 1.714285714 | 0.022631048 | 175 | 28 | 20887 | 12.78795918 | 0.99846338 | 0.33166565 | 0.315258162 |  |
| GO:0031965 nuclear membrane | 7 | 4 | 0.023439269 | 175 | 264 | 20887 | 3.16469697 | 0.998784123 | 0.33166565 | 0.315258162 |  |
| GO:1990423 RZZ complex | 2 | 1.142857143 | 0.024785183 | 175 | 3 | 20887 | 79.56952381 | 0.999177033 | 0.334009844 | 0.317486389 |  |
| GO:0042470 melanosome | 4 | 2.285714286 | 0.056237857 | 175 | 104 | 20887 | 4.590549451 | 0.999999923 | 0.574939378 | 0.546497147 |  |
| GO:0005658 alpha DNA polymerase:primase complex | 2 | 1.142857143 | 0.056884461 | 175 | 7 | 20887 | 34.10122449 | 0.999999937 | 0.574939378 | 0.546497147 |  |
| GO:0016604 nuclear body | 7 | 4 | 0.071890661 | 175 | 348 | 20887 | 2.400804598 | 0.999999999 | 0.639099766 | 0.607483523 |  |
| GO:0005664 nuclear origin of replication recognition complex | 2 | 1.142857143 | 0.072537933 | 175 | 9 | 20887 | 26.5231746 | 0.999999999 | 0.639099766 | 0.607483523 |  |
| GO:0031095 platelet dense tubular network membrane | 2 | 1.142857143 | 0.072537933 | 175 | 9 | 20887 | 26.5231746 | 0.999999999 | 0.639099766 | 0.607483523 |  |
| GO:0070161 anchoring junction | 3 | 1.714285714 | 0.074524001 | 175 | 54 | 20887 | 6.630793651 | 1 | 0.639099766 | 0.607483523 |  |
| GO:0005762 mitochondrial large ribosomal subunit | 3 | 1.714285714 | 0.081803928 | 175 | 57 | 20887 | 6.281804511 | 1 | 0.680897405 | 0.647213434 |  |
| GO:0009986 cell surface | 10 | 5.714285714 | 0.099096836 | 175 | 657 | 20887 | 1.816655795 | 1 | 0.779011237 | 0.740473579 |  |
| **Biological Process (BP)** | | | | | | | | | | | |
| **Term** | **Count** | **%** | **P-Value** | **List Total** | **Pop Hits** | **Pop Total** | **Fold Enrichment** | **Bonferroni** | **Benjamini** | **FDR** | **Associated KGs** |
| GO:0051301 cell division | 22 | 12.57142857 | 1.6953E-11 | 170 | 385 | 19734 | 6.633277311 | 2.10387E-08 | 1.95842E-08 | 1.93633E-08 | CDK1, BIRC5, CCNB2, CDC20, AURKB |
| GO:0090307 mitotic spindle assembly | 6 | 3.428571429 | 5.51893E-05 | 170 | 48 | 19734 | 14.51029412 | 0.066198945 | 0.006226361 | 0.006156121 | BIRC5, KIF20A, CDC20, AURKB |
| GO:0006915 apoptotic process | 21 | 12 | 1.86484E-07 | 170 | 593 | 19734 | 4.110842178 | 0.000231399 | 3.8571E-05 | 3.81359E-05 | CDK1, BIRC5, PTEN |
| GO:0000281 mitotic cytokinesis | 6 | 3.428571429 | 0.000358152 | 170 | 71 | 19734 | 9.809776305 | 0.358884688 | 0.030502939 | 0.030158828 | BIRC5, KIF20A, AURKB |
| GO:0016310 phosphorylation | 16 | 9.142857143 | 0.00036869 | 170 | 633 | 19734 | 2.934151101 | 0.367217458 | 0.030502939 | 0.030158828 | CDK1, AURKB, TK1 |
| GO:0006468 protein phosphorylation | 10 | 5.714285714 | 0.005210054 | 170 | 376 | 19734 | 3.087296621 | 0.99847014 | 0.269403198 | 0.266364 | CDK1, BIRC5, AURKB |
| GO:0007094 mitotic spindle assembly checkpoint signaling | 4 | 2.285714286 | 0.002325388 | 170 | 31 | 19734 | 14.97836812 | 0.944377467 | 0.144290319 | 0.142662547 | BIRC5, CDC20 |
| GO:0007052 mitotic spindle organization | 5 | 2.857142857 | 0.002760604 | 170 | 68 | 19734 | 8.535467128 | 0.967633994 | 0.163138529 | 0.161298127 | KIF20A, AURKB |
| GO:0034501 protein localization to kinetochore | 3 | 1.714285714 | 0.003811484 | 170 | 11 | 19734 | 31.65882353 | 0.991253385 | 0.205654402 | 0.203334368 | CDK1, AURKB |
| GO:0051256 mitotic spindle midzone assembly | 3 | 1.714285714 | 0.003811484 | 170 | 11 | 19734 | 31.65882353 | 0.991253385 | 0.205654402 | 0.203334368 | BIRC5, AURKB |
| GO:0000278 mitotic cell cycle | 6 | 3.428571429 | 0.008992913 | 170 | 148 | 19734 | 4.706041335 | 0.999986471 | 0.429238648 | 0.424396311 | BIRC5, AURKB |
| GO:0007346 regulation of mitotic cell cycle | 4 | 2.285714286 | 0.020601609 | 170 | 68 | 19734 | 6.828373702 | 1 | 0.878131129 | 0.868224735 | BIRC5, CDC20 |
| GO:0071897 DNA biosynthetic process | 3 | 1.714285714 | 0.02381947 | 170 | 28 | 19734 | 12.43739496 | 1 | 0.878131129 | 0.868224735 | TYMS, TK1 |
| GO:0062033 positive regulation of mitotic sister chromatid segregation | 2 | 1.142857143 | 0.025473586 | 170 | 3 | 19734 | 77.38823529 | 1 | 0.878131129 | 0.868224735 | CDK1, AURKB |
| GO:0009636 response to toxic substance | 4 | 2.285714286 | 0.029407684 | 170 | 78 | 19734 | 5.952941176 | 1 | 0.935767576 | 0.925210972 | CDK1, TYMS |
| GO:0043066 negative regulation of apoptotic process | 10 | 5.714285714 | 0.030835817 | 170 | 507 | 19734 | 2.28959276 | 1 | 0.956681215 | 0.945888679 | CDK1, BIRC5 |
| GO:1901970 positive regulation of mitotic sister chromatid separation | 2 | 1.142857143 | 0.033820602 | 170 | 4 | 19734 | 58.04117647 | 1 | 0.976078301 | 0.965066943 | BIRC5, AURKB |
| GO:1903490 positive regulation of mitotic cytokinesis | 2 | 1.142857143 | 0.050302011 | 170 | 6 | 19734 | 38.69411765 | 1 | 1 | 0.989516129 | BIRC5, AURKB |
| GO:1902425 positive regulation of attachment of mitotic spindle microtubules to kinetochore | 2 | 1.142857143 | 0.066503911 | 170 | 8 | 19734 | 29.02058824 | 1 | 1 | 0.989516129 | BIRC5, AURKB |
| GO:0000086 G2/M transition of mitotic cell cycle | 3 | 1.714285714 | 0.070774147 | 170 | 51 | 19734 | 6.828373702 | 1 | 1 | 0.989516129 | CDK1, BIRC5 |
| GO:1904668 positive regulation of ubiquitin protein ligase activity | 2 | 1.142857143 | 0.082431013 | 170 | 10 | 19734 | 23.21647059 | 1 | 1 | 0.989516129 | CDC20, PTEN |
| GO:0016477 cell migration | 6 | 3.428571429 | 0.094368449 | 170 | 281 | 19734 | 2.478626753 | 1 | 1 | 0.989516129 | CDK1, PTEN |
| GO:0090267 positive regulation of mitotic cell cycle spindle assembly checkpoint | 2 | 1.142857143 | 0.09808795 | 170 | 12 | 19734 | 19.34705882 | 1 | 1 | 0.989516129 | BIRC5, AURKB |
| GO:0006260 DNA replication | 13 | 7.428571429 | 4.50989E-11 | 170 | 98 | 19734 | 15.39867947 | 5.59676E-08 | 1.95842E-08 | 1.93633E-08 | CDK1 |
| GO:0006281 DNA repair | 15 | 8.571428571 | 7.75972E-08 | 170 | 267 | 19734 | 6.521480502 | 9.62935E-05 | 1.92596E-05 | 1.90424E-05 | CDK1 |
| GO:0010971 positive regulation of G2/M transition of mitotic cell cycle | 6 | 3.428571429 | 3.63748E-06 | 170 | 28 | 19734 | 24.87478992 | 0.004503945 | 0.000501568 | 0.000495909 | CDK1 |
| GO:0006974 DNA damage response | 11 | 6.285714286 | 0.000322009 | 170 | 307 | 19734 | 4.159302548 | 0.329463701 | 0.030502939 | 0.030158828 | CDK1 |
| GO:0007051 spindle organization | 4 | 2.285714286 | 0.000540506 | 170 | 19 | 19734 | 24.43839009 | 0.488776754 | 0.041922973 | 0.04145003 | AURKB |
| GO:0034644 cellular response to UV | 5 | 2.857142857 | 0.001440061 | 170 | 57 | 19734 | 10.18266254 | 0.832773095 | 0.105124451 | 0.103938518 | AURKB |
| GO:0007059 chromosome segregation | 6 | 3.428571429 | 0.002017602 | 170 | 104 | 19734 | 6.697058824 | 0.918436511 | 0.131781277 | 0.130294623 | BIRC5 |
| GO:0000079 regulation of cyclin-dependent protein serine/threonine kinase activity | 4 | 2.285714286 | 0.00759131 | 170 | 47 | 19734 | 9.879349186 | 0.99992184 | 0.376832609 | 0.372581476 | CCNB2 |
| GO:0010977 negative regulation of neuron projection development | 4 | 2.285714286 | 0.023064979 | 170 | 71 | 19734 | 6.53985087 | 1 | 0.878131129 | 0.868224735 | PTEN |
| GO:0007057 spindle assembly involved in female meiosis I | 2 | 1.142857143 | 0.033820602 | 170 | 4 | 19734 | 58.04117647 | 1 | 0.976078301 | 0.965066943 | CCNB2 |
| GO:0046683 response to organophosphorus | 2 | 1.142857143 | 0.033820602 | 170 | 4 | 19734 | 58.04117647 | 1 | 0.976078301 | 0.965066943 | TYMS |
| GO:0032467 positive regulation of cytokinesis | 3 | 1.714285714 | 0.048085208 | 170 | 41 | 19734 | 8.493830703 | 1 | 1 | 0.989516129 | AURKB |
| GO:0046718 symbiont entry into host cell | 4 | 2.285714286 | 0.058690619 | 170 | 103 | 19734 | 4.508052541 | 1 | 1 | 0.989516129 | CDK1 |
| GO:0045842 positive regulation of mitotic metaphase/anaphase transition | 2 | 1.142857143 | 0.09808795 | 170 | 12 | 19734 | 19.34705882 | 1 | 1 | 0.989516129 | CDC20 |
| GO:0006270 DNA replication initiation | 9 | 5.142857143 | 4.7343E-11 | 170 | 27 | 19734 | 38.69411765 | 5.87527E-08 | 1.95842E-08 | 1.93633E-08 |  |
| GO:0000727 double-strand break repair via break-induced replication | 6 | 3.428571429 | 3.2753E-08 | 170 | 12 | 19734 | 58.04117647 | 4.06457E-05 | 1.01616E-05 | 1.0047E-05 |  |
| GO:0006268 DNA unwinding involved in DNA replication | 6 | 3.428571429 | 7.90582E-07 | 170 | 21 | 19734 | 33.16638655 | 0.000980631 | 0.000140159 | 0.000138578 |  |
| GO:0030174 regulation of DNA-templated DNA replication initiation | 5 | 2.857142857 | 3.4945E-06 | 170 | 13 | 19734 | 44.64705882 | 0.004327296 | 0.000501568 | 0.000495909 |  |
| GO:0000070 mitotic sister chromatid segregation | 6 | 3.428571429 | 1.14468E-05 | 170 | 35 | 19734 | 19.89983193 | 0.014105081 | 0.001420542 | 0.001404517 |  |
| GO:0051988 regulation of attachment of spindle microtubules to kinetochore | 4 | 2.285714286 | 9.67954E-05 | 170 | 11 | 19734 | 42.21176471 | 0.113193841 | 0.010010254 | 0.009897326 |  |
| GO:0006271 DNA strand elongation involved in DNA replication | 3 | 1.714285714 | 0.001973452 | 170 | 8 | 19734 | 43.53088235 | 0.913833483 | 0.131781277 | 0.130294623 |  |
| GO:0032147 activation of protein kinase activity | 3 | 1.714285714 | 0.019222621 | 170 | 25 | 19734 | 13.92988235 | 1 | 0.878131129 | 0.868224735 |  |
| GO:0006310 DNA recombination | 4 | 2.285714286 | 0.022226841 | 170 | 70 | 19734 | 6.633277311 | 1 | 0.878131129 | 0.868224735 |  |
| GO:0000165 MAPK cascade | 5 | 2.857142857 | 0.023204767 | 170 | 126 | 19734 | 4.606442577 | 1 | 0.878131129 | 0.868224735 |  |
| GO:0070487 monocyte aggregation | 2 | 1.142857143 | 0.025473586 | 170 | 3 | 19734 | 77.38823529 | 1 | 0.878131129 | 0.868224735 |  |
| GO:1990036 calcium ion import into sarcoplasmic reticulum | 2 | 1.142857143 | 0.025473586 | 170 | 3 | 19734 | 77.38823529 | 1 | 0.878131129 | 0.868224735 |  |
| GO:0032470 positive regulation of endoplasmic reticulum calcium ion concentration | 2 | 1.142857143 | 0.025473586 | 170 | 3 | 19734 | 77.38823529 | 1 | 0.878131129 | 0.868224735 |  |
| GO:0032703 negative regulation of interleukin-2 production | 3 | 1.714285714 | 0.027108411 | 170 | 30 | 19734 | 11.60823529 | 1 | 0.909230743 | 0.898973506 |  |
| GO:0032729 positive regulation of type II interferon production | 4 | 2.285714286 | 0.029407684 | 170 | 78 | 19734 | 5.952941176 | 1 | 0.935767576 | 0.925210972 |  |
| GO:0008283 cell population proliferation | 5 | 2.857142857 | 0.041770227 | 170 | 152 | 19734 | 3.818498452 | 1 | 1 | 0.989516129 |  |
| GO:0010829 negative regulation of glucose transmembrane transport | 2 | 1.142857143 | 0.042096543 | 170 | 5 | 19734 | 46.43294118 | 1 | 1 | 0.989516129 |  |
| GO:1902975 mitotic DNA replication initiation | 2 | 1.142857143 | 0.042096543 | 170 | 5 | 19734 | 46.43294118 | 1 | 1 | 0.989516129 |  |
| GO:0040001 establishment of mitotic spindle localization | 2 | 1.142857143 | 0.042096543 | 170 | 5 | 19734 | 46.43294118 | 1 | 1 | 0.989516129 |  |
| GO:0010212 response to ionizing radiation | 3 | 1.714285714 | 0.048085208 | 170 | 41 | 19734 | 8.493830703 | 1 | 1 | 0.989516129 |  |
| GO:0034976 response to endoplasmic reticulum stress | 4 | 2.285714286 | 0.04947732 | 170 | 96 | 19734 | 4.836764706 | 1 | 1 | 0.989516129 |  |
| GO:0032687 negative regulation of interferon-alpha production | 2 | 1.142857143 | 0.050302011 | 170 | 6 | 19734 | 38.69411765 | 1 | 1 | 0.989516129 |  |
| GO:0045143 homologous chromosome segregation | 2 | 1.142857143 | 0.050302011 | 170 | 6 | 19734 | 38.69411765 | 1 | 1 | 0.989516129 |  |
| GO:0010468 regulation of gene expression | 6 | 3.428571429 | 0.050354902 | 170 | 233 | 19734 | 2.98924514 | 1 | 1 | 0.989516129 |  |
| GO:0002260 lymphocyte homeostasis | 2 | 1.142857143 | 0.058437603 | 170 | 7 | 19734 | 33.16638655 | 1 | 1 | 0.989516129 |  |
| GO:0000212 meiotic spindle organization | 2 | 1.142857143 | 0.058437603 | 170 | 7 | 19734 | 33.16638655 | 1 | 1 | 0.989516129 |  |
| GO:0007049 cell cycle | 7 | 4 | 0.060560568 | 170 | 324 | 19734 | 2.50795207 | 1 | 1 | 0.989516129 |  |
| GO:0042149 cellular response to glucose starvation | 3 | 1.714285714 | 0.063650987 | 170 | 48 | 19734 | 7.255147059 | 1 | 1 | 0.989516129 |  |
| GO:0045590 negative regulation of regulatory T cell differentiation | 2 | 1.142857143 | 0.066503911 | 170 | 8 | 19734 | 29.02058824 | 1 | 1 | 0.989516129 |  |
| GO:0002314 germinal center B cell differentiation | 2 | 1.142857143 | 0.066503911 | 170 | 8 | 19734 | 29.02058824 | 1 | 1 | 0.989516129 |  |
| GO:0031648 protein destabilization | 3 | 1.714285714 | 0.073203914 | 170 | 52 | 19734 | 6.697058824 | 1 | 1 | 0.989516129 |  |
| GO:0007519 skeletal muscle tissue development | 3 | 1.714285714 | 0.075660304 | 170 | 53 | 19734 | 6.570699223 | 1 | 1 | 0.989516129 |  |
| GO:0034080 CENP-A containing chromatin assembly | 2 | 1.142857143 | 0.082431013 | 170 | 10 | 19734 | 23.21647059 | 1 | 1 | 0.989516129 |  |
| GO:0070723 response to cholesterol | 2 | 1.142857143 | 0.082431013 | 170 | 10 | 19734 | 23.21647059 | 1 | 1 | 0.989516129 |  |
| GO:2000563 positive regulation of CD4-positive, alpha-beta T cell proliferation | 2 | 1.142857143 | 0.082431013 | 170 | 10 | 19734 | 23.21647059 | 1 | 1 | 0.989516129 |  |
| GO:0045959 negative regulation of complement activation, classical pathway | 2 | 1.142857143 | 0.082431013 | 170 | 10 | 19734 | 23.21647059 | 1 | 1 | 0.989516129 |  |
| GO:0046626 regulation of insulin receptor signaling pathway | 2 | 1.142857143 | 0.082431013 | 170 | 10 | 19734 | 23.21647059 | 1 | 1 | 0.989516129 |  |
| GO:0046638 positive regulation of alpha-beta T cell differentiation | 2 | 1.142857143 | 0.082431013 | 170 | 10 | 19734 | 23.21647059 | 1 | 1 | 0.989516129 |  |
| GO:0050852 T cell receptor signaling pathway | 4 | 2.285714286 | 0.087357971 | 170 | 122 | 19734 | 3.805978785 | 1 | 1 | 0.989516129 |  |
| GO:0072091 regulation of stem cell proliferation | 2 | 1.142857143 | 0.090292966 | 170 | 11 | 19734 | 21.10588235 | 1 | 1 | 0.989516129 |  |
| GO:0034755 iron ion transmembrane transport | 2 | 1.142857143 | 0.090292966 | 170 | 11 | 19734 | 21.10588235 | 1 | 1 | 0.989516129 |  |
| GO:0032508 DNA duplex unwinding | 3 | 1.714285714 | 0.090923145 | 170 | 59 | 19734 | 5.902492522 | 1 | 1 | 0.989516129 |  |
| GO:0042110 T cell activation | 3 | 1.714285714 | 0.093548789 | 170 | 60 | 19734 | 5.804117647 | 1 | 1 | 0.989516129 |  |
| GO:0042102 positive regulation of T cell proliferation | 3 | 1.714285714 | 0.09619627 | 170 | 61 | 19734 | 5.708968177 | 1 | 1 | 0.989516129 |  |
| **KEGG Pathways** | | | | | | | | | | | |
| hsa04110: Cell cycle | 17 | 9.714285714 | 3.4701E-12 | 91 | 158 | 8840 | 10.45207957 | 7.42604E-10 | 7.42601E-10 | 7.35661E-10 | CDK1, CCNB2, CDC20, AURKB |
| hsa04114: Oocyte meiosis | 8 | 4.571428571 | 0.000514909 | 91 | 139 | 8840 | 5.590955807 | 0.10436198 | 0.036730188 | 0.036386916 | CDK1, CCNB2, CDC20 |
| hsa04218: Cellular senescence | 7 | 4 | 0.005207332 | 91 | 157 | 8840 | 4.331210191 | 0.672831723 | 0.278592269 | 0.275988603 | CDK1, CCNB2, PTEN |
| hsa05166: Human T-cell leukemia virus 1 infection | 8 | 4.571428571 | 0.007512949 | 91 | 223 | 8840 | 3.484945548 | 0.800878713 | 0.321554236 | 0.318549057 | CCNB2, CDC20, PTEN |
| hsa04115: p53 signaling pathway | 4 | 2.285714286 | 0.040754824 | 91 | 75 | 8840 | 5.180952381 | 0.999864188 | 0.969059139 | 0.960002511 | CDK1, CCNB2, PTEN |
| hsa04068: FoxO signaling pathway | 6 | 3.428571429 | 0.010993499 | 91 | 132 | 8840 | 4.415584416 | 0.906110435 | 0.39210146 | 0.38843696 | CCNB2, PTEN |
| hsa00240: Pyrimidine metabolism | 4 | 2.285714286 | 0.02105015 | 91 | 58 | 8840 | 6.699507389 | 0.989462562 | 0.563091517 | 0.55782898 | TYMS, TK1 |
| hsa01232: Nucleotide metabolism | 4 | 2.285714286 | 0.055494866 | 91 | 85 | 8840 | 4.571428571 | 0.99999506 | 1 | 0.995305164 | TYMS, TK1 |
| hsa05215: Prostate cancer | 4 | 2.285714286 | 0.077905862 | 91 | 98 | 8840 | 3.965014577 | 0.999999971 | 1 | 0.995305164 | PTEN |
| hsa03030: DNA replication | 10 | 5.714285714 | 5.87474E-11 | 91 | 36 | 8840 | 26.98412698 | 1.2572E-08 | 6.28597E-09 | 6.22723E-09 |  |
| hsa04640: Hematopoietic cell lineage | 5 | 2.857142857 | 0.018162986 | 91 | 99 | 8840 | 4.906204906 | 0.980210643 | 0.555268429 | 0.550079005 |  |
| hsa04380: Osteoclast differentiation | 5 | 2.857142857 | 0.056576417 | 91 | 142 | 8840 | 3.420523139 | 0.999996134 | 1 | 0.995305164 |  |
| hsa04520: Adherens junction | 4 | 2.285714286 | 0.068870493 | 91 | 93 | 8840 | 4.178187404 | 0.999999767 | 1 | 0.995305164 |  |
| hsa03410: Base excision repair | 3 | 1.714285714 | 0.073638956 | 91 | 44 | 8840 | 6.623376623 | 0.999999922 | 1 | 0.995305164 |  |
