# Supplementary material for: In-silico identification of host-key-genes associated with dengue-virus-infections highlighting their pathogenetic mechanisms and therapeutic agents
Source: PLoS One. 2025 Oct 7;20(10):e0333509. doi: 10.1371/journal.pone.0333509 (PMC12503274; doi:10.1371/journal.pone.0333509)
Supplement: S4 Table — (DOCX) [file pone.0333509.s005.docx]

**S4 Table.** Collect protein structures from the database for molecular docking.

| **Protein Name** | **Uniprot ID** | **PDB ID** |
| --- | --- | --- |
| TYMS |  | 6qyq |
| CDC20 |  | 4ggc |
| PTEN |  | 7jul |
| CCNB2 | H0YMP3 |  |
| PPARG |  | 8b8y |
| CDK1 | E5RIU6 |  |
| GATA2 | P23769 |  |
| RELA |  | 1vj7 |
| TK1 |  | 2orv |
| FOXC1 | Q12948 |  |
| BIRC5 |  | 2qfa |
| TP53 |  | 8swj |
| AURKB | J3KRF8 |  |
| KIF20A |  | 6yip |
