# Supplementary material for: In-silico identification of host-key-genes associated with dengue-virus-infections highlighting their pathogenetic mechanisms and therapeutic agents
Source: PLoS One. 2025 Oct 7;20(10):e0333509. doi: 10.1371/journal.pone.0333509 (PMC12503274; doi:10.1371/journal.pone.0333509)
Supplement: S5 Table — (DOCX) [file pone.0333509.s006.docx]

**S5 Table.** Collection of DENVI-related candidate drugs from published articles and online web tools (DGIdb).

| **No.** | **Proposed drugs from other literature for DENVI inhibition (49)** | **Suggested Drug from DGIdb for the corresponding protein (134)** |
| --- | --- | --- |
| 1 | ZANAMIVIR [1–3] | PENCICLOVIR |
| 2 | JNJ-64281802 [2–5] | ARFOLITIXORIN |
| 3 | FAVIPIRAVIR [3–7] | RALTITREXED |
| 4 | BALAPIRAVIR [1–6,8–10] | ZIDOVUDINE |
| 5 | EFAVIRENZ [3–5,11] | EPOETIN ALFA |
| 6 | TIPRANAVIR [3–5,11] | MYCOPHENOLATE MOFETIL |
| 7 | DASABUVIR [3–5,11] | VALDECOXIB |
| 8 | LOVASTATIN [1,2,4,6,8,9] | PEMETREXED MONOHYDRATE |
| 9 | PROCHLORPERAZINE [4,6,8] | ARTESUNATE |
| 10 | BROMOCRIPTINE [3,4,8,9] | AZACITIDINE |
| 11 | CHLOROQUINE [1,2,4–6,8–10,12] | FENOFIBRIC ACID |
| 12 | 4-HPR [4,9,10,13] | CAPIVASERTIB |
| 13 | AR-12 [14–16] | FLOXURIDINE |
| 14 | BORTEZOMIB [4,5,10,17–19] | MYCOPHENOLATE |
| 15 | RIBAVIRIN [4–8,14,18–22] | ROSIGLITAZONE |
| 16 | SB203580 [23] | TEGAFUR |
| 17 | CASTANOSPERMINE [3–5,24] | PEMETREXED DISODIUM |
| 18 | NN-DNJ [19] | OMACETAXINE MEPESUCCINATE |
| 19 | PREDNISOLONE [1,6,8] | CAPMATINIB |
| 20 | SCHISANDRIN A [8] | ROFECOXIB |
| 21 | KETOTIFEN [1,2] | CALCITONIN |
| 22 | CROMOLYN [2] | PLICAMYCIN |
| 23 | MONTELUKAST [2] | THIOGUANINE |
| 24 | PINOSTROBIN [25] | PIOGLITAZONE HYDROCHLORIDE |
| 25 | 4-HYDROXYPANDURATIN A [18,19,25] | CINNARIZINE |
| 26 | DOXYCYCLINE [2,3,8,10,25,26] | TEMSIROLIMUS |
| 27 | MECLOFENAMIC ACID [25] | EVEROLIMUS |
| 28 | ROLITETRACYCLINE [3,25] | VORICONAZOLE |
| 29 | 7D [3] | ENCORAFENIB |
| 30 | RUTIN [7] | TERIPARATIDE |
| 31 | CELGOSIVIR [1–6,9,10] | ALPELISIB |
| 32 | CELASTROL [8,9] | PHENTOLAMINE |
| 33 | LACTIMIDOMYCIN [3,8,9,22] | ROSIGLITAZONE MALEATE |
| 34 | N-(4-HYDROXYPHENYL) RETINAMIDE (4-HPR) [9] | TIRBANIBULIN |
| 35 | PF-429242 [4,8,9] | LONAFARNIB |
| 36 | QL47 [9,22,27] | CLOFIBRATE |
| 37 | YKL-04-085 [9,22] | ENZALUTAMIDE |
| 38 | SARACATINIB [9] | INTERLEUKIN-11 |
| 39 | DASATINIB [3–5,9,28] | ASPARAGINASE |
| 40 | COBALT PROTOPORPHYRIN [9] | HYDROXYCHLOROQUINE |
| 41 | ANDROGRAPHOLIDE [8,9,18] | ZANUBRUTINIB |
| 42 | SUNITINIB [4–6,8,29] | BEZAFIBRATE |
| 43 | ERLOTINIB [4–6,8,30] | BALSALAZIDE DISODIUM |
| 44 | PROCHLOROPERAZINE [4,5] | OLSALAZINE SODIUM |
| 45 | PF-06409577 [2,4] | BALSALAZIDE |
| 46 | METFORMIN [2,4,9] | SERTRALINE HYDROCHLORIDE |
| 47 | AICAR [4,10] | TROGLITAZONE |
| 48 | MELATONIN [31,32] | TREPROSTINIL |
| 49 | NICLOSAMIDE [3–6,8,18] | HESPERETIN |
| 50 |  | FOLIC ACID |
| 51 |  | TOPOTECAN HYDROCHLORIDE |
| 52 |  | SULFASALAZINE |
| 53 |  | MITOMYCIN |
| 54 |  | SULINDAC |
| 55 |  | IRINOTECAN HYDROCHLORIDE |
| 56 |  | PROPYLPARABEN |
| 57 |  | INDOPROFEN |
| 58 |  | RESERPINE |
| 59 |  | FENOFIBRATE MICRONIZED |
| 60 |  | NIVOLUMAB |
| 61 |  | INTERFERON BETA-1A |
| 62 |  | BELUMOSUDIL |
| 63 |  | BENDAMUSTINE |
| 64 |  | TRIFLURIDINE |
| 65 |  | OBINUTUZUMAB |
| 66 |  | IDELALISIB |
| 67 |  | APIGENIN |
| 68 |  | VEMURAFENIB |
| 69 |  | ABIRATERONE ACETATE |
| 70 |  | BINIMETINIB |
| 71 |  | ARSENIC TRIOXIDE |
| 72 |  | TRASTUZUMAB |
| 73 |  | PRASTERONE |
| 74 |  | DABRAFENIB |
| 75 |  | CAPECITABINE |
| 76 |  | SAPANISERTIB |
| 77 |  | FLUTAMIDE |
| 78 |  | LEUCOVORIN CALCIUM |
| 79 |  | BENZALKONIUM CHLORIDE |
| 80 |  | PIRTOBRUTINIB |
| 81 |  | GLUTARAL |
| 82 |  | AFATINIB |
| 83 |  | RIBOCICLIB |
| 84 |  | CLOTRIMAZOLE |
| 85 |  | AZATHIOPRINE |
| 86 |  | CETUXIMAB |
| 87 |  | INDOMETHACIN |
| 88 |  | ERDAFITINIB |
| 89 |  | PEMBROLIZUMAB |
| 90 |  | PHENOLPHTHALEIN |
| 91 |  | PANITUMUMAB |
| 92 |  | HYDROCORTISONE BUTYRATE |
| 93 |  | OSIMERTINIB |
| 94 |  | TALAZOPARIB |
| 95 |  | TEMOZOLOMIDE |
| 96 |  | RESVERATROL |
| 97 |  | VERAPAMIL |
| 98 |  | PREDNISONE |
| 99 |  | COPANLISIB |
| 100 |  | DAUNORUBICIN LIPOSOMAL |
| 101 |  | ALECTINIB |
| 102 |  | SELINEXOR |
| 103 |  | ZINC CHLORIDE |
| 104 |  | TRABECTEDIN |
| 105 |  | IPILIMUMAB |
| 106 |  | NERATINIB |
| 107 |  | FLUOROURACIL |
| 108 |  | RUCAPARIB |
| 109 |  | ROMIDEPSIN |
| 110 |  | FULVESTRANT |
| 111 |  | NIRAPARIB |
| 112 |  | SULCONAZOLE NITRATE |
| 113 |  | ALEMTUZUMAB |
| 114 |  | DOXORUBICIN LIPOSOME |
| 115 |  | FURAZOLIDONE |
| 116 |  | LETROZOLE |
| 117 |  | LAPATINIB |
| 118 |  | MESALAMINE |
| 119 |  | DEXAMETHASONE |
| 120 |  | ETOPOSIDE |
| 121 |  | COBIMETINIB |
| 122 |  | OXALIPLATIN |
| 123 |  | CAMPTOTHECIN |
| 124 |  | DACTINOMYCIN |
| 125 |  | LEVONORGESTREL |
| 126 |  | EPIRUBICIN |
| 127 |  | TAMOXIFEN |
| 128 |  | RITUXIMAB |
| 129 |  | RISPERIDONE |
| 130 |  | VENETOCLAX |
| 131 |  | SELUMETINIB |
| 132 |  | TRAMETINIB DIMETHYL SULFOXIDE |
| 133 |  | GEFITINIB |
| 134 |  | GLYBURIDE |

**Reference**

1. Sinha, S.; Singh, K.; Ravi Kumar, Y.S.; Roy, R.; Phadnis, S.; Meena, V.; Bhattacharyya, S.; Verma, B. Dengue Virus Pathogenesis and Host Molecular Machineries. *J. Biomed. Sci.* **2024**, *31*, 1–24, doi:10.1186/s12929-024-01030-9.

2. Palanichamy Kala, M.; St. John, A.L.; Rathore, A.P.S. Dengue: Update on Clinically Relevant Therapeutic Strategies and Vaccines. *Curr. Treat. Options Infect. Dis.* **2023**, *15*, 27–52, doi:10.1007/s40506-023-00263-w.

3. Chauhan, N.; Gaur, K.K.; Asuru, T.R.; Guchhait, P. Dengue Virus: Pathogenesis and Potential for Small Molecule Inhibitors. *Biosci. Rep.* **2024**, *44*, 1–18, doi:10.1042/BSR20240134.

4. Tripathi, A.; Chauhan, S.; Khasa, R. A Comprehensive Review of the Development and Therapeutic Use of Antivirals in Flavivirus Infection. *Viruses* **2025**, *17*, 1–39, doi:10.3390/v17010074.

5. Diani, E.; Lagni, A.; Lotti, V.; Tonon, E.; Cecchetto, R.; Gibellini, D. Vector-Transmitted Flaviviruses: An Antiviral Molecules Overview. *Microorganisms* **2023**, *11*, 1–38, doi:10.3390/microorganisms11102427.

6. Komarasamy, T.V.; Adnan, N.A.A.; James, W.; Balasubramaniam, V.R.M.T. Finding a Chink in Armor: Update, Limitations, and Challenges toward Successful Antivirals against Flaviviruses. *PLoS Negl. Trop. Dis.* **2022**, *16*, 1–30, doi:10.1371/journal.pntd.0010291.

7. Kumar, S.; Bajrai, L.H.; Faizo, A.A.; Khateb, A.M.; Alkhaldy, A.A.; Rana, R.; Azhar, E.I.; Dwivedi, V.D. Pharmacophore-Model-Based Drug Repurposing for the Identification of the Potential Inhibitors Targeting the Allosteric Site in Dengue Virus NS5 RNA-Dependent RNA Polymerase. *Viruses* **2022**, *14*, doi:10.3390/v14081827.

8. Dighe, S.N.; Ekwudu, O.; Dua, K.; Chellappan, D.K.; Katavic, P.L.; Collet, T.A. Recent Update on Anti-Dengue Drug Discovery. *Eur. J. Med. Chem.* **2019**, *176*, 431–455, doi:10.1016/j.ejmech.2019.05.010.

9. Tian, Y.S.; Zhou, Y.; Takagi, T.; Kameoka, M.; Kawashita, N. Dengue Virus and Its Inhibitors: A Brief Review. *Chem. Pharm. Bull.* **2018**, *66*, 191–206, doi:10.1248/cpb.c17-00794.

10. Murarik, M.R. Identification of Biomarkers for the Prediction of Dengue Disease Severity Using High- Throughput Proteomics. **2023**.

11. Stefanik, M.; Valdes, J.J.; Ezebuo, F.C.; Haviernik, J.; Uzochukwu, I.C.; Fojtikova, M.; Salat, J.; Eyer, L.; Ruzek, D. Fda-Approved Drugs Efavirenz, Tipranavir, and Dasabuvir Inhibit Replication of Multiple Flaviviruses in Vero Cells. *Microorganisms* **2020**, *8*, doi:10.3390/microorganisms8040599.

12. El-Shamy, N.T.; Alkaoud, A.M.; Hussein, R.K.; Ibrahim, M.A.; Alhamzani, A.G.; Abou-Krisha, M.M. DFT, ADMET and Molecular Docking Investigations for the Antimicrobial Activity of 6,6′-Diamino-1,1′,3,3′-Tetramethyl-5,5′-(4-Chlorobenzylidene)Bis[Pyrimidine-2,4(1H,3H)-Dione]. *Molecules* **2022**, *27*, 1–17, doi:10.3390/molecules27030620.

13. Devignot, S.; Sapet, C.; Duong, V.; Bergon, A.; Rihet, P.; Ong, S.; Lorn, P.T.; Chroeung, N.; Ngeav, S.; Tolou, H.J.; et al. Genome-Wide Expression Profiling Deciphers Host Responses Altered during Dengue Shock Syndrome and Reveals the Role of Innate Immunity in Severe Dengue. *PLoS One* **2010**, *5*, doi:10.1371/journal.pone.0011671.

14. Yang, C.F.; Gopula, B.; Liang, J.J.; Li, J.K.; Chen, S.Y.; Lee, Y.L.; Chen, C.S.; Lin, Y.L. Novel AR-12 Derivatives, P12-23 and P12-34, Inhibit Flavivirus Replication by Blocking Host de Novo Pyrimidine Biosynthesis. *Emerg. Microbes Infect.* **2018**, *7*, doi:10.1038/s41426-018-0191-1.

15. Hsin-Hsin Chen 1, Chien-Chin Chen 2, Yee-Shin Lin 3, Po-Chun Chang 1, Zi-Yi Lu 1, Chiou-Feng Lin 4, Chia-Ling Chen 5, C.-P.C. 6 AR-12 Suppresses Dengue Virus Replication by down-Regulation of PI3K/AKT and GRP78. **2017**, doi:10.1016/j.antiviral.2017.02.015.

16. Hassandarvish, P.; Oo, A.; Jokar, A.; Zukiwski, A.; Proniuk, S.; Bakar, S.A.; Zandi, K. Exploring the in Vitro Potential of Celecoxib Derivative AR-12 as an Effective Antiviral Compound against Four Dengue Virus Serotypes. *J. Antimicrob. Chemother.* **2017**, *72*, 2438–2442, doi:10.1093/jac/dkx191.

17. Gebhart, N.N.; Hardy, R.W.; Sokoloski, K.J. Comparative Analyses of Alphaviral RNA: Protein Complexes Reveals Conserved Host-Pathogen Interactions. *PLoS One* **2020**, *15*, 1–23, doi:10.1371/journal.pone.0238254.

18. Zhu, Y.; Chen, S.; Lurong, Q.; Qi, Z. Recent Advances in Antivirals for Japanese Encephalitis Virus. *Viruses* **2023**, *15*, 1–21, doi:10.3390/v15051033.

19. Joe, S.; Salam, A.A.A.; Neogi, U.; N, N.B.; Mudgal, P.P. Antiviral Drug Research for Japanese Encephalitis: An Updated Review. *Pharmacol. Reports* **2022**, *74*, 273–296, doi:10.1007/s43440-022-00355-2.

20. Rahman, M.A.; Chakma, U.; Kumer, A.; Rahman, M.R.; Matin, M.M. Uridine-Derived 4-Aminophenyl 1-Thioglucosides: DFT Optimized FMO, ADME, and Antiviral Activities Study. *Biointerface Res. Appl. Chem.* **2023**, *13*, 1–15, doi:10.33263/BRIAC131.052.

21. Ye, J.; Zhu, B.; Fu, Z.F.; Chen, H.; Cao, S. Immune Evasion Strategies of Flaviviruses. *Vaccine* **2013**, *31*, 461–471, doi:10.1016/j.vaccine.2012.11.015.

22. Press, P.; Ra, D.O.I.; Wispelaere, X.M. De; Carocci, M.; Burri, D.J.; Neidermyer, W.J.; Olson, C.M.; Roggenbach, I.; Liang, Y.; Wang, J.; et al. A Broad-Spectrum Antiviral Molecule , QL47 , Selectively Inhibits Eukaryotic Translation. **2020**, *295*, 1694–1703, doi:10.1074/jbc.RA119.011132.

23. Ye, H.; Kang, L.; Yan, X.; Li, S.; Huang, Y.; Mu, R.; Duan, X.; Chen, L. MiR-103a-3p Promotes Zika Virus Replication by Targeting OTU Deubiquitinase 4 to Activate P38 Mitogen-Activated Protein Kinase Signaling Pathway. *Front. Microbiol.* **2022**, *13*, doi:10.3389/fmicb.2022.862580.

24. Mahmud, S.; Afrose, S.; Biswas, S.; Nagata, A.; Paul, G.K.; Mita, M.A.; Hasan, M.R.; Shimu, M.S.S.; Zaman, S.; Uddin, M.S.; et al. Plant-Derived Compounds Effectively Inhibit the Main Protease of SARS-CoV-2: An in Silico Approach. *PLoS One* **2022**, *17*, 1–19, doi:10.1371/journal.pone.0273341.

25. Othman, R.; Othman, R.; Baharuddin, A.; Ramakrishnan, N.R.; Rahman, N.A.; Yusof, R.; Karsani, S.A. Molecular Docking Studies of Selected Medicinal Drugs as Dengue Virus-2 Protease Inhibitors. *Sains Malaysiana* **2017**, *46*, 1865–1875, doi:10.17576/jsm-2017-4610-25.

26. Tian, B.; Widen, S.G.; Yang, J.; Wood, T.G.; Kudlicki, A.; Zhao, Y.; Brasier, A.R. The NFκB Subunit RELA Is a Master Transcriptional Regulator of the Committed Epithelial-Mesenchymal Transition in Airway Epithelial Cells. *J. Biol. Chem.* **2018**, *293*, 16528–16545, doi:10.1074/jbc.RA118.003662.

27. Yanke Liang †,‡, Melissanne de Wispelaere §, Margot Carocci §, Qingsong Liu †,‡, Jinhua Wang †,‡, Priscilla L Yang §,*, N.S.G. Structure–Activity Relationship Study of QL47: A Broad-Spectrum Antiviral Agent. **2017**, doi:10.1021/acsmedchemlett.7b00008.

28. Escudero-fl, M.; Torres-hoyos, D.; Miranda-brand, Y.; Boudreau, R.L.; Carlos, J.; Vicente-manzanares, M. Dengue Virus Infection Alters Inter-Endothelial Junctions and Promotes Endothelial–Mesenchymal-Transition-like Changes in Human Microvascular Endothelial Cells. **2023**.

29. Daina, A.; Michielin, O.; Zoete, V. SwissADME: A Free Web Tool to Evaluate Pharmacokinetics, Drug-Likeness and Medicinal Chemistry Friendliness of Small Molecules. *Sci. Rep.* **2017**, *7*, 1–13, doi:10.1038/srep42717.

30. Kanehisa, M.; Furumichi, M.; Tanabe, M.; Sato, Y.; Morishima, K. KEGG: New Perspectives on Genomes, Pathways, Diseases and Drugs. *Nucleic Acids Res.* **2017**, *45*, D353–D361, doi:10.1093/nar/gkw1092.

31. Ghafouri-Fard, S.; Khoshbakht, T.; Hussen, B.M.; Abdullah, S.T.; Taheri, M.; Samadian, M. A Review on the Role of Mir-16-5p in the Carcinogenesis. *Cancer Cell Int.* **2022**, *22*, 1–11, doi:10.1186/s12935-022-02754-0.

32. Chen, S.Y.; Chen, Y.L.; Li, P.C.; Cheng, T.S.; Chu, Y.S.; Shen, Y.S.; Chen, H.T.; Tsai, W.N.; Huang, C.L.; Sieber, M.; et al. Engineered Extracellular Vesicles Carrying Let-7a-5p for Alleviating Inflammation in Acute Lung Injury. *J. Biomed. Sci.* **2024**, *31*, 1–18, doi:10.1186/s12929-024-01019-4.
