# Supplementary material for: In-silico identification of host-key-genes associated with dengue-virus-infections highlighting their pathogenetic mechanisms and therapeutic agents
Source: PLoS One. 2025 Oct 7;20(10):e0333509. doi: 10.1371/journal.pone.0333509 (PMC12503274; doi:10.1371/journal.pone.0333509)
Supplement: S6 Table — (DOCX) [file pone.0333509.s007.docx]

**S6 Table.** Binding affinity score within drugs and corresponding proteins and TFs.

| **Compound name** | **Compound ID (CID)** | **AURKB** | **BIRC5** | **CCNB2** | **CDC20** | **CDK1** | **KIF20A** | **PTEN** | **TK1** | **TYMS** | **FOXC1** | **GATA2** | **PPARG** | **RELA** | **TP53** | **Average** |
| --- | --- | --- | --- | --- | --- | --- | --- | --- | --- | --- | --- | --- | --- | --- | --- | --- |
| ENTRECTINIB | 25141092 | -7.1 | -8.3 | -9.6 | -9.9 | -9.6 | -7.4 | -9.7 | -9.5 | -10.5 | -8.6 | -9.6 | -9.8 | -7.9 | -6.9 | **-8.885714** |
| AR-12 | 10027278 | -8.1 | -8.7 | -8.8 | -10.6 | -10 | -7 | -7.7 | -7.7 | -10.7 | -8.3 | -9.5 | -9.4 | -8.4 | -7.6 | **-8.75** |
| LONAFARNIB | 148195 | -7.2 | -7.4 | -9.2 | -10.6 | -9.2 | -7.8 | -10.2 | -8.1 | -9.7 | -8.5 | -8.6 | -9.1 | -8.6 | -7.8 | **-8.714286** |
| IMATINIB | 5291 | -6.5 | -8.3 | -10 | -9.4 | -9.1 | -6.9 | -9.6 | -8.1 | -10.5 | -8.3 | -8.3 | -10 | -8.2 | -8 | **-8.657143** |
| BROMOCRIPTINE | 31101 | -7.5 | -7.7 | -9.6 | -10 | -7.8 | -6.8 | -9.7 | -8.2 | -10.5 | -8.2 | -8.4 | -9.1 | -9 | -7.5 | **-8.571429** |
| QL47 | 71748056 | -6.9 | -7.2 | -8.9 | -10 | -8.1 | -7.3 | -9.6 | -8.5 | -9.8 | -8.4 | -9.3 | -8.9 | -8.4 | -7.1 | **-8.457143** |
| SORAFENIB | 216239 | -6 | -8.5 | -8.6 | -9.6 | -9.3 | -6.4 | -9.9 | -8 | -9.5 | -8.2 | -8.7 | -9.2 | -8.2 | -7.8 | **-8.421429** |
| DASABUVIR | 56640146 | -8.2 | -8 | -8.4 | -9.3 | -8.4 | -6.8 | -9.8 | -8 | -9.1 | -8.2 | -8.4 | -8.7 | -8.1 | -7.6 | **-8.357143** |
| ZYTIGA | 9821849 | -6.8 | -8 | -9.5 | -8.6 | -9 | -6.7 | -9.6 | -7.4 | -9.9 | -7.9 | -7.3 | -9.1 | -8.5 | -7.5 | **-8.271429** |
| CELASTROL | 122724 | -7 | -7.6 | -9.6 | -9.6 | -7.9 | -6.6 | -9.6 | -7.6 | -10.4 | -7.7 | -8.5 | -7.7 | -8.7 | -7 | **-8.25** |
| TIPRANAVIR | 54682461 | -6.3 | -7.2 | -9.4 | -8.3 | -8.8 | -6.7 | -9.6 | -9.1 | -8.8 | -7 | -8.7 | -8.6 | -7.8 | -8.7 | **-8.214286** |
| DABRAFENIB | 44462760 | -6.6 | -7 | -8.8 | -9.5 | -9.4 | -6.8 | -8.3 | -9.3 | -9.3 | -7.1 | -8.4 | -9 | -7.9 | -7.1 | **-8.178571** |
| MOSNODENVIR | 122697854 | -6.1 | -7.4 | -8.5 | -8.8 | -8.4 | -6.5 | -8.9 | -8.3 | -9.3 | -7.2 | -8.9 | -9.3 | -8.3 | -8.3 | **-8.157143** |
| MONTELUKAST | 5281040 | -7 | -7.4 | -8.2 | -8.8 | -9.4 | -6.4 | -8.8 | -7.8 | -9.3 | -7.9 | -8.3 | -9.3 | -7.6 | -7.4 | **-8.114286** |
| ALPELISIB | 56649450 | -6.2 | -7.8 | -8.6 | -8.6 | -9.2 | -6.3 | -8.9 | -8.3 | -9.1 | -7.3 | -8.4 | -9.1 | -8.8 | -6.8 | **-8.1** |
| ARFOLITIXORIN | 135398645 | -6.2 | -8.1 | -8.7 | -9.5 | -8.7 | -6 | -8.4 | -7.9 | -10.4 | -7.5 | -8.3 | -9 | -7.7 | -6.9 | **-8.092857** |
| CHEMBL4064534 | 137637635 | -6.4 | -7.1 | -9 | -9.2 | -8.4 | -6.9 | -8.9 | -7.4 | -9.1 | -7.8 | -8.8 | -8.4 | -8.3 | -7.4 | **-8.078571** |
| TALAZOPARIB | 135565082 | -6.4 | -7.8 | -9 | -9.5 | -7.8 | -6.9 | -8.4 | -7.6 | -8.9 | -7.6 | -8.2 | -8.6 | -8.4 | -7.9 | **-8.071429** |
| TRAMETINIB | 11707110 | -7.1 | -8.2 | -8.4 | -9.6 | -7.8 | -6 | -8.3 | -7.8 | -9.7 | -8.9 | -7.3 | -9 | -7.4 | -7.3 | **-8.057143** |
| RUTIN | 5280805 | -5.8 | -7 | -8.4 | -10.1 | -8.6 | -5.5 | -8.2 | -8.3 | -9.9 | -7.2 | -8.1 | -8.3 | -8.6 | -6.6 | **-7.9** |
| LACTIMIDOMYCIN | 11669726 | -6.4 | -6.8 | -8.8 | -8.2 | -8.5 | -5.6 | -8.4 | -7.1 | -9.9 | -8.2 | -8.2 | -8.9 | -7.8 | -7.5 | **-7.878571** |
| SB203580 | 176155 | -6.3 | -6.6 | -8.9 | -8.5 | -8.9 | -6.1 | -9.3 | -7.6 | -9.4 | -7.4 | -7.7 | -8.1 | -7.5 | -6.7 | **-7.785714** |
| EPIRUBICIN | 41867 | -6.3 | -7.4 | -7.9 | -9.3 | -8.3 | -5.8 | -8.1 | -7.6 | -8.5 | -7.4 | -7.7 | -8.7 | -8.9 | -6.9 | **-7.771429** |
| CHEMBL3891221 | 71748255 | -6.8 | -7.9 | -8.3 | -8.6 | -8.2 | -6.1 | -8.3 | -6.9 | -9.5 | -7.8 | -7.9 | -7.5 | -7.4 | -6.9 | **-7.721429** |
| AZD6482 | 44137675 | -5.8 | -7.2 | -8 | -8.5 | -8.6 | -5.8 | -8 | -7.9 | -8.6 | -7.5 | -8.6 | -8 | -7.8 | -7.1 | **-7.671429** |
| FENRETINIDE | 5288209 | -6.5 | -7.3 | -8.4 | -7.4 | -7.7 | -6.5 | -9.3 | -7 | -9.4 | -7.9 | -7.1 | -7.9 | -6.9 | -7.6 | **-7.635714** |
| RALTITREXED | 135400182 | -5.2 | -7.1 | -8.4 | -8.7 | -8.2 | -5.7 | -8.2 | -8.5 | -8.7 | -7.2 | -7.6 | -7.8 | -8.1 | -7.2 | **-7.614286** |
| RUCAPARIB | 9931954 | -5.9 | -6.8 | -8.6 | -8.4 | -8.8 | -5.8 | -8.2 | -6.9 | -8.9 | -7.4 | -8.1 | -7.9 | -8.1 | -6.5 | **-7.592857** |
| E6201 | 10172827 | -6.3 | -6.8 | -8 | -8.8 | -7.8 | -5.8 | -8.1 | -7 | -9 | -7.4 | -8.1 | -8.1 | -8.3 | -6.8 | **-7.592857** |
| COBALTIPROTOPORPHYRIN | 4971 | -6.3 | -7.1 | -7.8 | -8.5 | -7.8 | -6.1 | -8.7 | -7.2 | -9 | -7.5 | -7 | -7.5 | -8.1 | -7.6 | **-7.585714** |
| RESERPINE | 5770 | -6.2 | -6.9 | -8.1 | -9.3 | -8.2 | -5.5 | -8.3 | -7.2 | -9.3 | -7.3 | -7.9 | -8.1 | -7.8 | -6.1 | **-7.585714** |
| DASATINIB | 3062316 | -5.6 | -7.2 | -8.3 | -9 | -8 | -5.8 | -8.6 | -7.8 | -9.6 | -7.5 | -7.4 | -7.4 | -7 | -6.9 | **-7.578571** |
| INFIGRATINIB | 53235510 | -5.8 | -7.2 | -8.9 | -8.9 | -7.7 | -6.2 | -8.3 | -6.9 | -9.1 | -7.3 | -7.5 | -7.6 | -7.8 | -6.9 | **-7.578571** |
| BAY 61-3606 | 10200390 | -6.1 | -7 | -7.6 | -9 | -8.1 | -5.5 | -7.8 | -8.1 | -8.3 | -7.5 | -8.3 | -8.5 | -7.2 | -7 | **-7.571429** |
| COBIMETINIB | 16222096 | -5.9 | -7.2 | -8.4 | -8.1 | -8 | -5.9 | -8.3 | -9.1 | -8.1 | -6.7 | -7.4 | -9 | -8.1 | -5.8 | **-7.571429** |
|  | 0_uff_E=457.54 | -5.7 | -8.5 | -8.1 | -7.6 | -8.7 | -5.7 | -7.7 | -7.8 | -7.6 | -7.6 | -8.4 | -7.5 | -8.3 | -6.7 | **-7.564286** |
|  | 5352062 | -6 | -7.3 | -7.8 | -9.8 | -7.4 | -5.5 | -8.4 | -7.4 | -8.7 | -7.2 | -6.6 | -9.2 | -7.8 | -6.8 | **-7.564286** |
|  | 0_uff_E=697.66 | -5.5 | -7.4 | -7.8 | -8.3 | -8.1 | -5.7 | -8.8 | -8.1 | -8.5 | -7.7 | -7.5 | -8.3 | -7.1 | -7 | **-7.557143** |
|  | 135398512 | -6.7 | -6.9 | -7.8 | -9 | -8 | -6 | -7.8 | -7.1 | -8.1 | -7.5 | -8.1 | -7.7 | -7.3 | -7.8 | **-7.557143** |
|  | 60700 | -6.4 | -7 | -7.9 | -8.6 | -8.4 | -5.9 | -7.9 | -7.8 | -9.1 | -7.8 | -7.5 | -7.9 | -7.2 | -6.4 | **-7.557143** |
|  | 10302451 | -6.1 | -7 | -8.3 | -9 | -7.9 | -6.1 | -8.3 | -7.4 | -8.8 | -7.6 | -6.9 | -8.4 | -7.1 | -6.8 | **-7.55** |
|  | 5994 | -6.2 | -7.9 | -8.4 | -8.2 | -8.4 | -6.2 | -8.3 | -7.1 | -8.7 | -7 | -6.7 | -8.1 | -7.7 | -6.8 | **-7.55** |
|  | 5865 | -6.3 | -7.3 | -7.6 | -9 | -7.8 | -6.4 | -7.8 | -7.4 | -8.8 | -7 | -7.9 | -7.4 | -8.2 | -6.7 | **-7.542857** |
|  | 23643976 | -6.3 | -6.8 | -7.7 | -8.6 | -8.3 | -5.9 | -7.6 | -7.9 | -8.5 | -7.2 | -7.6 | -7.5 | -7.9 | -7.7 | **-7.535714** |
|  | 57384863 | -5.4 | -6.8 | -7.3 | -9.3 | -8.5 | -5.9 | -7.7 | -7.7 | -8.7 | -7.2 | -7.5 | -8.4 | -7.8 | -7 | **-7.514286** |
|  | 135564570 | -5.7 | -7.3 | -8.6 | -8.2 | -8.7 | -6.1 | -8 | -7.3 | -8.4 | -7.5 | -7.5 | -7.8 | -7.3 | -6.8 | **-7.514286** |
|  | 9907093 | -5.4 | -7.2 | -7.5 | -8.5 | -8.8 | -5.7 | -7.9 | -7.3 | -8.6 | -7.6 | -7.4 | -8.4 | -7.6 | -7.2 | **-7.507143** |
|  | 0_uff_E=759.30 | -5.9 | -7.2 | -8.2 | -9.1 | -8.6 | -5.8 | -7.3 | -6.7 | -8.2 | -7.1 | -8.2 | -9 | -6.6 | -6.9 | **-7.485714** |
|  | 24788740 | -5.5 | -7.1 | -7.8 | -8.8 | -9.1 | -6 | -8.2 | -7.3 | -8.8 | -6.7 | -7 | -7.2 | -7.7 | -7.5 | **-7.478571** |
|  | 9915743 | -6.4 | -6.7 | -7.9 | -8.6 | -7.3 | -6.1 | -8.5 | -7.8 | -9 | -6.8 | -6.8 | -8.6 | -7.2 | -7 | **-7.478571** |
|  | 6918852 | -5.7 | -7.6 | -7.6 | -9 | -8.6 | -5.8 | -8.5 | -7.1 | -8.8 | -6.8 | -7 | -8.1 | -7.6 | -6.4 | **-7.471429** |
|  | 17751819 | -5.9 | -7.4 | -8 | -7.8 | -8.4 | -5.6 | -8.2 | -6.7 | -8.6 | -7.5 | -7.5 | -8.6 | -7.9 | -6.4 | **-7.464286** |
|  | 3827 | -6.1 | -7.3 | -7.9 | -8.3 | -7.5 | -6.5 | -7.7 | -7 | -8.5 | -6.9 | -7.3 | -7.7 | -7.8 | -7.7 | **-7.442857** |
|  | 57519748 | -5.7 | -6.9 | -7.7 | -8.9 | -7.9 | -6.3 | -8.5 | -7.2 | -8.2 | -7.4 | -7.6 | -8 | -7.1 | -6.5 | **-7.421429** |
|  | 16219401 | -6.1 | -7.9 | -7.9 | -8.1 | -8.5 | -5.8 | -8.1 | -7.4 | -8.2 | -7.1 | -7.7 | -7.1 | -7.4 | -6.4 | **-7.407143** |
|  | 10184653 | -6.1 | -6.4 | -7.7 | -8.2 | -8.1 | -6 | -7.9 | -7.9 | -8.5 | -7 | -7.1 | -8.8 | -6.9 | -7.1 | **-7.407143** |
|  | 58298318 | -5.9 | -8.6 | -7.2 | -9.2 | -7.8 | -5.5 | -8.2 | -7.1 | -8.5 | -7.1 | -7.6 | -7.1 | -7.4 | -6.5 | **-7.407143** |
|  | 135565635 | -5.8 | -7.7 | -8.1 | -8.2 | -8.4 | -5.6 | -7.7 | -7.5 | -8.7 | -7.7 | -6.9 | -7.2 | -7.1 | -7 | **-7.4** |
|  | 3081361 | -5.5 | -6.6 | -8.5 | -8.5 | -8.1 | -6.4 | -8.3 | -7 | -8.7 | -7.8 | -7.2 | -7.5 | -7.1 | -6.4 | **-7.4** |
|  | 46940575 | -6.4 | -6.3 | -7.9 | -8.8 | -8.1 | -5.6 | -8.4 | -6.7 | -7.9 | -7.5 | -8.2 | -8.1 | -7.3 | -6.3 | **-7.392857** |
|  | 25227436 | -5.8 | -6.5 | -7.7 | -8.7 | -8.5 | -5.6 | -7.7 | -7.3 | -9 | -6.8 | -7.4 | -7.6 | -7.8 | -7 | **-7.385714** |
|  | 992586 | -5.7 | -6.5 | -7.9 | -7.9 | -8.5 | -6.1 | -8.3 | -7.6 | -8.2 | -6.9 | -7.5 | -8 | -7.5 | -6.7 | **-7.378571** |
|  | 66414 | -6.1 | -7.7 | -7.7 | -7.9 | -8.4 | -5.8 | -7.6 | -7 | -8.1 | -7.3 | -8.3 | -7.5 | -7.7 | -6.1 | **-7.371429** |
|  | 5881 | -6 | -8.2 | -8.1 | -7.7 | -8.1 | -5.8 | -7.9 | -7.2 | -8.1 | -7.1 | -8.1 | -7.3 | -7.2 | -6.2 | **-7.357143** |
|  | 5743 | -6.1 | -7 | -8.2 | -8.2 | -8.1 | -5.8 | -7.9 | -7.8 | -9.2 | -6.2 | -6.6 | -7.3 | -8 | -6.5 | **-7.35** |
|  | 16007391 | -5.3 | -5.9 | -8.2 | -8.2 | -7.7 | -6.1 | -8.3 | -8.1 | -8.2 | -6.9 | -7.8 | -8.6 | -6.9 | -6.6 | **-7.342857** |
|  | 54682938 | -5.9 | -6.6 | -8.4 | -9.5 | -6.6 | -5.6 | -8.1 | -7.4 | -8.6 | -7 | -7.7 | -7.1 | -8.3 | -6 | **-7.342857** |
|  | 636530 | -6 | -7.6 | -8.1 | -7.8 | -6.7 | -5.8 | -7.7 | -7.8 | -8.7 | -7.2 | -7.4 | -7.1 | -7.9 | -6.9 | **-7.335714** |
|  | 51042438 | -5.8 | -6.6 | -7.8 | -8.2 | -8 | -5.8 | -7.4 | -8 | -8.2 | -6.8 | -7.6 | -7.9 | -7.2 | -7.2 | **-7.321429** |
|  | 46926350 | -6 | -7.1 | -7.7 | -8.4 | -8 | -6 | -7.5 | -7.5 | -8.1 | -6.6 | -7.6 | -7.9 | -7 | -7.1 | **-7.321429** |
|  | 50922675 | -5.5 | -6.7 | -7.2 | -8.7 | -8 | -4.7 | -7.8 | -7.8 | -8.7 | -7.2 | -8.4 | -7.1 | -7.4 | -7.1 | **-7.307143** |
|  | 5755 | -6.2 | -7 | -7.3 | -8.7 | -7.6 | -6.3 | -7.6 | -7.1 | -8.4 | -6.8 | -7.6 | -7.1 | -8 | -6.6 | **-7.307143** |
|  | 73201 | -5.8 | -6.8 | -7.7 | -7.8 | -7.8 | -5.5 | -7.6 | -7.4 | -8.5 | -7 | -7.7 | -7.7 | -7.3 | -7.1 | **-7.264286** |
|  | 16725726 | -5.9 | -6.7 | -7.5 | -8.4 | -8.2 | -5.6 | -7.5 | -8.1 | -8.2 | -6.9 | -7.3 | -7.6 | -7 | -6.5 | **-7.242857** |
|  | 97214 | -5.6 | -7 | -7.5 | -8.1 | -7.7 | -5.4 | -7.6 | -8.6 | -7.9 | -7.1 | -7.7 | -7.7 | -7.6 | -5.8 | **-7.235714** |
|  | 49784945 | -5 | -6.8 | -7.1 | -8.7 | -8.7 | -5.9 | -7.9 | -7.4 | -8.3 | -7.1 | -7.3 | -7.4 | -7.5 | -6.1 | **-7.228571** |
|  | 6420138 | -5.4 | -6.8 | -7.9 | -8.4 | -8.3 | -5 | -7.8 | -7.1 | -8.1 | -7.1 | -7.6 | -7.9 | -7.6 | -6.2 | **-7.228571** |
|  | 53232 | -5.9 | -6.7 | -7.8 | -7.9 | -8.8 | -5.5 | -8 | -6.8 | -8 | -7 | -7.1 | -8.5 | -6.5 | -6.6 | **-7.221429** |
|  | 24180719 | -5.4 | -7.1 | -7.8 | -7.6 | -8.3 | -5.8 | -8.3 | -7.3 | -7.9 | -6.9 | -8 | -7.5 | -7 | -6.1 | **-7.214286** |
|  | 45375953 | -5.5 | -7.7 | -7.6 | -8.5 | -8 | -5.3 | -7.7 | -7.2 | -7.8 | -6.9 | -8.1 | -7.6 | -6.7 | -6.4 | **-7.214286** |
|  | 71496458 | -6.2 | -6.7 | -7.1 | -8.1 | -7.3 | -5.5 | -7.5 | -7.9 | -8.4 | -7.3 | -7 | -7.9 | -7.2 | -6.8 | **-7.207143** |
|  | 948954 | -5.4 | -7.5 | -7.1 | -7.4 | -7.9 | -5.7 | -7.5 | -7.1 | -7.7 | -6.7 | -7.2 | -7.9 | -7.3 | -8.5 | **-7.207143** |
|  | 1_uff_E=228.13 | -6 | -7.3 | -7.8 | -7.3 | -7.6 | -5.9 | -8.4 | -6.7 | -8.5 | -6.9 | -7.3 | -7.1 | -7.3 | -6.8 | **-7.207143** |
|  | 1548887 | -5.8 | -6.5 | -8 | -8.5 | -7.1 | -5.9 | -8 | -6.9 | -8.3 | -7.3 | -7.6 | -7.3 | -6.9 | -6.7 | **-7.2** |
|  | 46214930 | -5.5 | -6 | -7.7 | -8 | -8.3 | -5.6 | -8.2 | -8 | -8.1 | -6.3 | -7.4 | -7.8 | -6.8 | -7 | **-7.192857** |
|  | 67462786 | -5.8 | -7.2 | -7.3 | -8.1 | -7.8 | -5.1 | -7.9 | -7.1 | -8.2 | -6.8 | -7.3 | -8.1 | -7.5 | -6.4 | **-7.185714** |
|  | 119607 | -6.1 | -7 | -7.6 | -7.6 | -8.5 | -5.6 | -8.4 | -7.1 | -8.2 | -6.6 | -8.1 | -6.7 | -6.3 | -6.7 | **-7.178571** |
|  | 54671203 | -5.9 | -6.6 | -7.4 | -8.6 | -6.8 | -5.1 | -7.7 | -6.8 | -9.7 | -6.5 | -7.1 | -7.5 | -8.1 | -6.5 | **-7.164286** |
|  | 59190723 | -6.2 | -6.9 | -7.6 | -8 | -7.1 | -5.7 | -7.8 | -8.2 | -7.9 | -6.6 | -7 | -7.6 | -6.9 | -6.7 | **-7.157143** |
|  | 5090 | -6 | -6.9 | -7.5 | -7.4 | -8.3 | -5.7 | -7.7 | -7.9 | -8 | -6.4 | -7.5 | -7.3 | -6.5 | -7 | **-7.15** |
|  | 4713 | -5.7 | -6.7 | -7.5 | -7.8 | -8 | -5.8 | -7.7 | -7 | -7.8 | -6.9 | -7.3 | -7.6 | -7.6 | -6.7 | **-7.15** |
|  | 2882 | -5.1 | -6.1 | -7 | -9 | -8.8 | -4.4 | -6.9 | -6.9 | -8.2 | -8 | -8.1 | -8.5 | -7.8 | -5.3 | **-7.15** |
|  | 5329102 | -5.4 | -6.4 | -7.8 | -8 | -7.9 | -5.8 | -8.2 | -7.7 | -7.8 | -6.9 | -7.3 | -7.2 | -7.1 | -6.4 | **-7.135714** |
|  | 11717001 | -5.9 | -7.1 | -7.8 | -7.8 | -8.3 | -5.4 | -7.6 | -6.7 | -8.2 | -7 | -7.5 | -7.3 | -7 | -6.2 | **-7.128571** |
|  | 46191454 | -5.8 | -6.2 | -7.5 | -8.1 | -7.5 | -5.6 | -8.1 | -7.3 | -8 | -6.9 | -7.9 | -7.6 | -7 | -6.3 | **-7.128571** |
|  | 3902 | -5.3 | -7.1 | -8.3 | -7.6 | -7.9 | -5.8 | -8.1 | -6.4 | -8.5 | -6.9 | -6.9 | -6.8 | -7.5 | -6.2 | **-7.092857** |
|  | 46912230 | -5.5 | -6.4 | -7.3 | -8.9 | -7.9 | -5.7 | -7.6 | -7.5 | -8.1 | -7 | -6.8 | -7.7 | -6.7 | -6.2 | **-7.092857** |
|  | 11716665 | -5.4 | -7.6 | -7.4 | -7.8 | -7.8 | -5.2 | -7.3 | -6.9 | -8.2 | -6.6 | -7.9 | -7.7 | -6.7 | -6.7 | **-7.085714** |
|  | 5280961 | -5.5 | -6.7 | -8.4 | -7.9 | -7.5 | -5.2 | -8 | -7.1 | -8.3 | -6.8 | -7.4 | -6.8 | -7.6 | -6 | **-7.085714** |
|  | 16747683 | -5.4 | -7.2 | -7 | -7.9 | -8.3 | -5.2 | -7 | -7.1 | -8 | -6.7 | -7.8 | -7.4 | -7 | -7 | **-7.071429** |
|  | 5311382 | -5.6 | -6.1 | -8.2 | -8.1 | -8.1 | -5.8 | -7.9 | -6.8 | -8.1 | -7.1 | -7 | -6.7 | -6.9 | -6.5 | **-7.064286** |
|  | 9811611 | -5 | -6.4 | -8.2 | -7.8 | -7.8 | -5.6 | -7.8 | -7.6 | -7.8 | -6.3 | -6.8 | -8.3 | -7 | -6.5 | **-7.064286** |
|  | 4477 | -5.3 | -7.3 | -7.4 | -7.9 | -7.6 | -5.4 | -8.1 | -7.2 | -7.5 | -6.9 | -7.2 | -7.4 | -7.3 | -6.3 | **-7.057143** |
|  | 16654980 | -5.2 | -7.1 | -7.4 | -8.7 | -7.1 | -5.5 | -7.5 | -7.6 | -8.1 | -7.2 | -7 | -6.9 | -7.3 | -6.1 | **-7.05** |
|  | 9826528 | -5.4 | -7.5 | -7.2 | -7.8 | -7.7 | -5.7 | -7.4 | -7.8 | -8.4 | -6.9 | -7.3 | -7.1 | -6.8 | -5.7 | **-7.05** |
|  | 444795 | -6.1 | -5.9 | -8 | -8 | -7.4 | -6.1 | -7.5 | -6.1 | -8.4 | -6.8 | -6.5 | -8.1 | -7 | -6.7 | **-7.042857** |
|  | 5318517 | -5.5 | -6.4 | -7.8 | -8 | -7.7 | -5.7 | -7.5 | -6.9 | -8.1 | -6.4 | -7.8 | -7.3 | -7.2 | -5.9 | **-7.014286** |
|  | 3339 | -5.9 | -6.6 | -7.6 | -7.2 | -7.9 | -5.5 | -8.1 | -6.4 | -8 | -6.9 | -6.7 | -8.1 | -7.2 | -6 | **-7.007143** |
|  | 3715 | -5.9 | -6.2 | -7 | -8.2 | -7.7 | -5.3 | -7.7 | -6.8 | -8.5 | -6.7 | -7.4 | -7.3 | -7 | -6.4 | **-7.007143** |
|  | 123631 | -4.8 | -5.7 | -7.6 | -8.2 | -7.5 | -5.6 | -8.1 | -6.6 | -8 | -6.4 | -7.1 | -8.1 | -7.5 | -6.9 | **-7.007143** |
|  | 16123056 | -5.7 | -6.4 | -7 | -8 | -8.2 | -5.7 | -7.2 | -7.2 | -8 | -7.1 | -7.1 | -7.4 | -7 | -6 | **-7** |
|  | 11338033 | -5.4 | -6.8 | -7.3 | -8.1 | -7.4 | -5.4 | -8.1 | -6.7 | -7.9 | -6.6 | -7.3 | -7.4 | -7 | -6.1 | **-6.964286** |
|  | 1_uff_E=476.15 | -5.2 | -7.1 | -7.3 | -7.6 | -8 | -5.8 | -7 | -6.8 | -7.2 | -6.6 | -7 | -8 | -7.1 | -6.8 | **-6.964286** |
|  | 104741 | -5.6 | -5.3 | -8.2 | -7.7 | -7.7 | -5.3 | -7.8 | -6.7 | -8.2 | -6.6 | -6.5 | -8.3 | -6 | -7.6 | **-6.964286** |
|  | 2812 | -5.7 | -8 | -7.7 | -7 | -6.8 | -4.9 | -7.7 | -6.5 | -9 | -6.5 | -6.9 | -7.5 | -6.6 | -6.3 | **-6.935714** |
|  | 135400184 | -5 | -7.7 | -7.1 | -7.8 | -7.4 | -5.4 | -7.3 | -7.2 | -7.7 | -6.5 | -7.3 | -7.1 | -7 | -6.2 | **-6.907143** |
|  | 1_uff_E=479.69 | -5.1 | -7.1 | -7.2 | -7.6 | -7.9 | -5.7 | -7 | -6.8 | -7.5 | -6.6 | -6.9 | -7.4 | -6.7 | -7.1 | **-6.9** |
|  | 10288191 | -5.4 | -6.2 | -7.3 | -8.2 | -7.8 | -5.5 | -7.2 | -6.7 | -7.8 | -7 | -7.1 | -7.5 | -6.4 | -6.5 | **-6.9** |
|  | 10126189 | -5.6 | -6.1 | -6.6 | -8.3 | -8.5 | -5.2 | -7.3 | -6.8 | -7.9 | -6.5 | -7 | -7.5 | -6.9 | -6.2 | **-6.885714** |
|  | 10127622 | -5.2 | -6.3 | -7.3 | -8.2 | -7.7 | -5.3 | -7.2 | -6.7 | -7.6 | -6.9 | -7.1 | -7.7 | -6.7 | -6.4 | **-6.878571** |
|  | 45380979 | -5.4 | -5.9 | -6.9 | -8.2 | -7.8 | -5.1 | -7.4 | -6.8 | -8.2 | -6.7 | -6.9 | -6.9 | -7 | -7 | **-6.871429** |
|  | 2733526 | -6.4 | -6.7 | -7.7 | -7.2 | -7.6 | -5.2 | -7.5 | -6 | -7.9 | -6.1 | -6.6 | -8.4 | -6.3 | -6.6 | **-6.871429** |
|  | 176870 | -5.7 | -6.1 | -7.2 | -7.2 | -7.4 | -5.7 | -7.2 | -7.5 | -7.4 | -6.7 | -6.6 | -7.9 | -7.1 | -6.2 | **-6.85** |
|  | 26133 | -5.1 | -6.4 | -7.4 | -7.7 | -6.6 | -5.6 | -7.7 | -7.1 | -8.3 | -6.3 | -6.8 | -7 | -5.9 | -6.5 | **-6.742857** |
|  | 445154 | -5.5 | -7.3 | -7.1 | -6.8 | -6.7 | -5.3 | -7.3 | -7.8 | -7.5 | -6.6 | -7 | -7.2 | -6.5 | -5.8 | **-6.742857** |
|  | 285033 | -5.1 | -6.3 | -6.9 | -8.2 | -7.4 | -4.6 | -7.6 | -7.8 | -8 | -5.8 | -6.6 | -7.1 | -7.2 | -5.7 | **-6.735714** |
|  | 64139 | -5.8 | -6.9 | -7 | -6.7 | -7.2 | -5.4 | -7.1 | -7.6 | -7.3 | -6.5 | -6.6 | -6.7 | -6.5 | -6.9 | **-6.728571** |
|  | 3547 | -5.2 | -6.1 | -7.1 | -7.1 | -7.7 | -5.3 | -6.8 | -7.5 | -7.7 | -6.4 | -7.9 | -6.3 | -7.4 | -5.4 | **-6.707143** |
|  | 11960529 | -5.2 | -7.1 | -7.1 | -7.4 | -7.2 | -5.2 | -6.8 | -6.8 | -7.4 | -6.7 | -7.3 | -7 | -7 | -5.7 | **-6.707143** |
|  | 9700 | -4.7 | -6.6 | -6.9 | -7.3 | -7.1 | -4.8 | -6.8 | -10 | -7.8 | -6.4 | -6.7 | -6.1 | -7.3 | -5.4 | **-6.707143** |
|  | 5775 | -5.3 | -6.6 | -7.4 | -7.1 | -7.6 | -5.1 | -6.9 | -6.3 | -8.2 | -6.4 | -6.3 | -7.2 | -6.7 | -6.4 | **-6.678571** |
|  | 60953 | -4.8 | -6.7 | -6.8 | -7.3 | -7.3 | -5.1 | -7.3 | -7.6 | -7.2 | -6.7 | -7.2 | -6.7 | -6.8 | -5.9 | **-6.671429** |
|  | 5379 | -5.4 | -6.1 | -6.9 | -8.6 | -7.5 | -4.9 | -7.1 | -6.3 | -7.3 | -6.5 | -6.7 | -6.8 | -7.5 | -5.7 | **-6.664286** |
|  | 448014 | -4.7 | -6.9 | -6.8 | -7.6 | -7.2 | -5.2 | -7.1 | -6.7 | -7.5 | -6.1 | -6.8 | -7.2 | -6.6 | -5.8 | **-6.585714** |
|  | 4917 | -5 | -6.3 | -7.5 | -7.3 | -6.9 | -5.7 | -7.4 | -6.5 | -7.2 | -6.2 | -7.3 | -6.8 | -6.3 | -5.7 | **-6.578571** |
|  | 5746 | -4.9 | -7 | -6.6 | -7.6 | -7.7 | -4.7 | -6.7 | -6.6 | -7.8 | -6.5 | -7.2 | -6.5 | -6.6 | -5.6 | **-6.571429** |
|  | 4037 | -5.1 | -5.8 | -7 | -6.7 | -7.2 | -5.2 | -7.4 | -7 | -7.1 | -6.4 | -6.4 | -7 | -6.9 | -6.6 | **-6.557143** |
|  | 129849014 | -5.1 | -6.6 | -7 | -6.9 | -7.8 | -5.2 | -6.7 | -6 | -7 | -6.8 | -6.7 | -6.9 | -6.9 | -6 | **-6.542857** |
|  | 446541 | -4.4 | -6.5 | -6.9 | -7.2 | -7.5 | -5.5 | -7.1 | -6.8 | -7.4 | -6.4 | -6.7 | -7 | -5.8 | -5.6 | **-6.485714** |
|  | 3397 | -5 | -6.2 | -6.8 | -6.9 | -6.9 | -5.1 | -7.2 | -7.1 | -7.1 | -6 | -7.2 | -7.2 | -6.3 | -5.7 | **-6.478571** |
|  | 9849735 | -5.5 | -5.7 | -6.8 | -8.2 | -7.3 | -5.2 | -6.6 | -6 | -8 | -5.8 | -6.3 | -6.5 | -7.2 | -5.6 | **-6.478571** |
|  | 9813758 | -5.6 | -6.9 | -6.3 | -7.2 | -7.5 | -4.9 | -6.7 | -6.4 | -7.2 | -6.2 | -6.9 | -6.7 | -6 | -5.4 | **-6.421429** |
|  | 6918736 | -4.3 | -6 | -6.9 | -7.7 | -7.1 | -5 | -6.9 | -6.7 | -7.3 | -5.8 | -7.1 | -6.9 | -6.7 | -5.2 | **-6.4** |
|  | 23661637 | -5.9 | -5.8 | -6.6 | -7 | -7.3 | -4.7 | -7 | -6.8 | -7.7 | -5.8 | -7.3 | -6.7 | -5.9 | -5 | **-6.392857** |
|  | 11691726 | -4.9 | -5.6 | -6.7 | -7.4 | -7.1 | -4.5 | -7.1 | -7.4 | -7.7 | -5.1 | -6.2 | -6.7 | -6.7 | -6.1 | **-6.371429** |
|  | 65063 | -4.8 | -6.7 | -6.5 | -7 | -7.1 | -4.5 | -6.7 | -6.7 | -7.3 | -6.3 | -7 | -6.1 | -7.1 | -5.4 | **-6.371429** |
|  | 60855 | -4.9 | -5.5 | -6.4 | -7.9 | -7 | -4.5 | -6.2 | -6.8 | -7.5 | -5.6 | -6.8 | -6.3 | -6.9 | -5.4 | **-6.264286** |
|  | 11715767 | -4.8 | -6.8 | -6.6 | -6.9 | -6.8 | -4.9 | -6.6 | -6 | -7 | -6.1 | -6.9 | -6.4 | -6 | -5.4 | **-6.228571** |
|  | 0_uff_E=331.38 | -4.6 | -5.5 | -6.3 | -6.6 | -6.8 | -4.8 | -6.4 | -7.4 | -7.2 | -5.8 | -6.2 | -7.3 | -5.9 | -5.7 | **-6.178571** |
|  | 18343 | -4.7 | -6 | -6.9 | -6.7 | -6.6 | -4.3 | -6.3 | -6.8 | -7.2 | -6 | -7.2 | -5.7 | -6.3 | -5.2 | **-6.135714** |
|  | 37542 | -4.7 | -6.4 | -5.8 | -7.2 | -6.5 | -4.8 | -6.2 | -6.6 | -6.8 | -5.9 | -6.8 | -5.7 | -6.6 | -5.4 | **-6.1** |
|  | 5311 | -4.5 | -6.1 | -6.4 | -6 | -6.5 | -4.3 | -6.6 | -7.7 | -6.7 | -6.1 | -6.1 | -6.7 | -6.1 | -5.6 | **-6.1** |
|  | 5790 | -4.2 | -5.6 | -6.3 | -6.5 | -6.6 | -4.3 | -6.2 | -8.6 | -6.7 | -5.7 | -7.1 | -5.9 | -5.9 | -5.4 | **-6.071429** |
|  | 35370 | -4.5 | -5.6 | -6.3 | -6.6 | -6.7 | -4.5 | -6.5 | -6.4 | -6.6 | -6 | -6.8 | -6.2 | -6.7 | -5.3 | **-6.05** |
|  | 2520 | -4.3 | -5.4 | -6.2 | -6.4 | -7.2 | -5.1 | -7.2 | -6.6 | -6.1 | -6.2 | -5.6 | -6.3 | -6.6 | -5.5 | **-6.05** |
|  | 2265 | -4.3 | -5.8 | -6.5 | -7 | -6.4 | -4 | -6 | -7.4 | -6.8 | -5.5 | -6.3 | -5.7 | -7.1 | -5.4 | **-6.014286** |
|  | 896 | -4.4 | -6.6 | -5.9 | -6.4 | -6.5 | -4.5 | -6.5 | -5.8 | -6.7 | -6 | -6.4 | -6.8 | -6.3 | -5.4 | **-6.014286** |
|  | 155256 | -5.1 | -5.4 | -6.3 | -7.1 | -5.9 | -5.3 | -6.5 | -6.6 | -7.1 | -5.6 | -5.9 | -6.4 | -5.8 | -5.1 | **-6.007143** |
|  | 60750 | -4.5 | -5.7 | -6.2 | -6.4 | -6.9 | -4.3 | -6.2 | -7.2 | -6.5 | -5.4 | -6.8 | -6.1 | -6.4 | -5 | **-5.971429** |
|  | 17513 | -4.4 | -6.2 | -6 | -7.2 | -6.5 | -4.3 | -6 | -6.4 | -6.7 | -5.8 | -6.7 | -5.5 | -6.3 | -4.9 | **-5.921429** |
|  | 5394 | -4.5 | -6.5 | -6.1 | -7.2 | -6 | -4.2 | -5.7 | -6.6 | -6.4 | -5.5 | -6.5 | -5.4 | -6.6 | -5.5 | **-5.907143** |
|  | 3652 | -4.5 | -5.4 | -6.8 | -6.4 | -6.8 | -4.6 | -6.4 | -6.1 | -6.6 | -5.7 | -5.5 | -6 | -6.3 | -5.2 | **-5.878571** |
|  | 9444 | -4.3 | -5.6 | -6.2 | -7 | -6.2 | -4.1 | -5.9 | -6.1 | -7 | -5.6 | -6.7 | -5.8 | -6.3 | -5.3 | **-5.864286** |
|  | 60734 | -4.5 | -5.6 | -6.2 | -6.5 | -6.7 | -4.2 | -6 | -6.3 | -6.8 | -5.6 | -6.4 | -6.2 | -5.9 | -5.1 | **-5.857143** |
|  | 5386 | -4.1 | -5.3 | -6.4 | -6.3 | -6.4 | -4.2 | -6.1 | -6.7 | -6.2 | -5.4 | -6.6 | -5.6 | -5.9 | -5.1 | **-5.735714** |
|  | 24801581 | -3.9 | -5.1 | -5.9 | -6.3 | -6.2 | -4.6 | -6.7 | -5.8 | -6 | -5.5 | -6.1 | -6.3 | -5.9 | -4.9 | **-5.657143** |
|  | 6253 | -4.5 | -5.7 | -5.9 | -6.5 | -5.9 | -4 | -6.1 | -6.1 | -6.6 | -5.1 | -6.5 | -5.1 | -6 | -5.1 | **-5.65** |
|  | 53248678 | -4.7 | -5.3 | -6 | -6.4 | -6.3 | -4 | -5.9 | -6.2 | -6.2 | -5.4 | -5.8 | -6 | -5.9 | -4.9 | **-5.642857** |
|  | 13711 | -4 | -5.2 | -5.8 | -6.3 | -6.2 | -4.1 | -5.7 | -5.8 | -6.5 | -5.3 | -6.9 | -5.6 | -6.3 | -4.9 | **-5.614286** |
|  | 135398748 | -3.8 | -5.4 | -5.5 | -6.9 | -6.1 | -4.1 | -6.1 | -6.7 | -6.1 | -5.5 | -6.1 | -5.1 | -6.2 | -4.9 | **-5.607143** |
|  | 501640 | -4.3 | -4.9 | -5.6 | -6.1 | -6.2 | -4.5 | -6 | -5.5 | -6.7 | -5.4 | -5.7 | -5.7 | -5.9 | -5 | **-5.535714** |
|  | 5353431 | -3.7 | -5.5 | -6.2 | -5.5 | -6 | -4.3 | -5.8 | -6 | -7 | -5.1 | -5.6 | -5.5 | -5.2 | -6.1 | **-5.535714** |
|  | 2796 | -4.2 | -5.8 | -5.6 | -5.7 | -5.9 | -4.2 | -5.9 | -5.7 | -6.2 | -5 | -5.3 | -6.2 | -6 | -5.3 | **-5.5** |
|  | 2719 | -3.8 | -5.1 | -5.8 | -6.2 | -6.4 | -4.4 | -6 | -5 | -6.3 | -5.7 | -5.4 | -5 | -5.8 | -5.1 | **-5.428571** |
|  | 54445 | -4 | -5.6 | -5.7 | -6 | -5.5 | -4 | -5.5 | -5.9 | -5.9 | -4.8 | -5.7 | -5 | -5.7 | -4.9 | **-5.3** |
|  | 4696 | -3.7 | -5.3 | -5.6 | -5.6 | -5.3 | -4 | -5.7 | -5.9 | -5.1 | -4.9 | -6.2 | -5.7 | -5.7 | -5.4 | **-5.292857** |
|  | 43157 | -3.7 | -5.2 | -5.4 | -5.6 | -5.8 | -4.2 | -5.7 | -6.8 | -5.5 | -4.8 | -5.5 | -5.4 | -5.5 | -5 | **-5.292857** |
|  | 492405 | -3.6 | -5.5 | -5.1 | -5.6 | -6.4 | -3.7 | -5.1 | -6.7 | -5.3 | -4.5 | -5.5 | -4.9 | -5.3 | -5.4 | **-5.185714** |
|  | 557205 | -3.8 | -5.3 | -5.4 | -5.7 | -5.2 | -4.1 | -5.4 | -5.9 | -5.7 | -4.6 | -4.6 | -5.9 | -5 | -4.8 | **-5.1** |
|  | 3385 | -3.6 | -4.6 | -5 | -5.1 | -5.3 | -3.6 | -5.2 | -5.9 | -4.7 | -4.1 | -5.1 | -4.8 | -5 | -4.5 | **-4.75** |
|  | 2568 | -3.6 | -4.9 | -5.2 | -4.7 | -4.8 | -3.9 | -5.6 | -5.1 | -4.7 | -4.4 | -5 | -4.6 | -5.2 | -4.4 | **-4.721429** |
|  | 4091 | -3.5 | -4.5 | -4.6 | -5.1 | -4.6 | -3.3 | -4.9 | -4.7 | -5.1 | -4.1 | -4.7 | -4.5 | -4.7 | -3.9 | **-4.442857** |
|  | 3117 | -2.8 | -4.1 | -4.5 | -4.2 | -4.9 | -3.1 | -4.2 | -4.2 | -4.4 | -3.7 | -3.9 | -4.1 | -4 | -3.5 | **-3.971429** |
