# Supplementary material for: In-silico identification of host-key-genes associated with dengue-virus-infections highlighting their pathogenetic mechanisms and therapeutic agents
Source: PLoS One. 2025 Oct 7;20(10):e0333509. doi: 10.1371/journal.pone.0333509 (PMC12503274; doi:10.1371/journal.pone.0333509)
Supplement: S7 Table — (DOCX) [file pone.0333509.s008.docx]

**S7 Table.** Drug-likeness profile of top 30 drugs.

| **Molecule properties** | | | | | | | | **Lipinski Rule** | |
| --- | --- | --- | --- | --- | --- | --- | --- | --- | --- |
| **Compounds** | **Molecular weight (g/mol)** | **Log P_o/w_ (MLOGP)** | **Water solubility-**  **Log S (ESOL)** | **H-bond Acceptor (HBA)** | **H-bond donor (HBD)** | **TPSA (Å^2^)** | **No of rotatable bond** | **Follows** | **Violation** |
| **ENTRECTINIB** | 560.649 | 4.00 | -3.161 | 6 | 3 | 237.218 | 7 | 3 | 1 |
| **AR-12** | 460.45 | 3.89 | -6.14 | 6 | 2 | 72.94 | 6 | 4 | 0 |
| **LONAFARNIB** | 638.82 | 4.62 | -6.71 | 3 | 1 | 79.53 | 5 | 2 | 2 |
| **IMATINIB** | 493.60 | 2.15 | -5.07 | 6 | 2 | 86.28 | 8 | 4 | 0 |
| **BROMOCRIPTINE** | 654.59 | 1.61 | -6.67 | 6 | 3 | 118.21 | 6 | 3 | 1 |
| **QL47** | 447.49 | 3.12 | -4.95 | 4 | 0 | 73.02 | 4 | 4 | 0 |
| **SORAFENIB** | 464.82 | 2.91 | -5.11 | 7 | 3 | 92.35 | 9 | 4 | 0 |
| **DASABUVIR** | 493.57 | 2.74 | -5.65 | 5 | 2 | 118.64 | 6 | 4 | 0 |
| **ZYTIGA** | 391.55 | 4.68 | -5.50 | 3 | 0 | 39.19 | 3 | 3 | 1 |
| **CELASTROL** | 450.61 | 4.42 | -6.31 | 4 | 2 | 74.60 | 1 | 3 | 0 |
| **TIPRANAVIR** | 602.66 | 3.74 | -7.49 | 9 | 2 | 113.97 | 12 | 3 | 1 |
| **DABRAFENIB** | 519.56 | 2.94 | -6.16 | 8 | 2 | 147.48 | 6 | 3 | 1 |
| **MOSNODENVIR** | 582.98 | 2.38 | -7.05 | 9 | 2 | 115.10 | 10 | 3 | 1 |
| **MONTELUKAST** | 586.18 | 5.70 | -7.94 | 4 | 2 | 95.72 | 12 | 2 | 2 |
| **ALPELISIB** | 441.47 | 1.59 | -4.42 | 7 | 2 | 129.45 | 7 | 4 | 0 |
| **ARFOLITIXORIN** | 457.44 | -0.48 | -2.11 | 7 | 6 | 193.98 | 8 | 3 | 1 |
| **CHEMBL4064534** | 491.58 | 3.44 | -5.51 | 4 | 1 | 72.16 | 7 | 4 | 0 |
| **TALAZOPARIB** | 380.35 | 3.16 | -4.04 | 6 | 2 | 88.49 | 2 | 4 | 0 |
| **TRAMETINIB** | 615.39 | 4.21 | -5.86 | 5 | 2 | 107.13 | 6 | 2 | 2 |
| **RUTIN** | 610.52 | -3.89 | -3.30 | 16 | 10 | 269.43 | 6 | 1 | 3 |
| **LACTIMIDOMYCIN** | 457.56 | 2.96 | -4.389 | 6 | 2 | 109.77 | 7 | 4 | 0 |
| **SB203580** | 377.43 | 2.79 | -4.56 | 4 | 1 | 77.85 | 4 | 4 | 0 |
| **EPIRUBICIN** | 543.52 | -2.10 | -3.91 | 12 | 6 | 206.07 | 5 | 1 | 3 |
| **CHEMBL3891221** | 341.79 | 3.12 | -4.52 | 3 | 3 | 73.32 | 3 | 4 | 0 |
| **AZD6482** | 408.45 | 0.60 | -3.67 | 5 | 2 | 96.17 | 5 | 4 | 0 |
| **FENRETINIDE** | 391.55 | 6.8623 | -6.239 | 2 | 2 | 174.782 | 7 | 3 | 1 |
| **RALTITREXED** | 458.49 | 1.05 | -3.26 | 7 | 4 | 180.93 | 10 | 4 | 1 |
| **RUCAPARIB** | 323.36 | 2.60 | -3.68 | 3 | 3 | 56.92 | 3 | 4 | 0 |
| **E6201** | 389.44 | 1.05 | -4.34 | 6 | 4 | 116.09 | 2 | 4 | 0 |
| **COBALTIPROTO-**  **PORPHYRIN** | 619.58 | 2.15 | -6.24 | 8 | 2 | 125.10 | 8 | 3 | 1 |
