# Supplementary material for: In-silico identification of host-key-genes associated with dengue-virus-infections highlighting their pathogenetic mechanisms and therapeutic agents
Source: PLoS One. 2025 Oct 7;20(10):e0333509. doi: 10.1371/journal.pone.0333509 (PMC12503274; doi:10.1371/journal.pone.0333509)
Supplement: S8 Table — (DOCX) [file pone.0333509.s009.docx]

**S8 Table.** Collection of DENVI-related highlighted genes from publications by others.

| **No.** | **Independent receptors published by other** |
| --- | --- |
| 1 | CD38 [1] |
| 2 | ADH6 [2] |
| 3 | CXCR3 [3] |
| 4 | CCNB2 [4] |
| 5 | IRF7 [5] |
| 6 | HSPA5 [6] |
| 7 | OAS1 [7,8] |
| 8 | PTGS2 [6] |
| 9 | STAT1 [4,7,8] |
| 10 | SYK [9] |
| 11 | ATF3[6] |
| 12 | MX1 [7,8] |
| 13 | CDKN1C[1] |
| 14 | IFI27 [3,7,8,10] |
| 15 | SOCS3 [11] |
| 16 | ANXA9 [2] |
| 17 | NR0B2 [2] |
| 18 | IFI44L [7,8] |
| 19 | STAT2 [3,12] |
| 20 | C1orf12 [2,13] |
| 21 | CDT1 [14,15] |
| 22 | IL6 [6] |
| 23 | IFI35 [7,8] |
| 24 | ISG15 [7,8] |
| 25 | IFI6 [7,8] |
| 26 | USP18 [7,8,16] |
| 27 | KCTD14 [10,14] |
| 28 | NFKB1 [13] |
| 29 | CAV1 [6] |
| 30 | IL12A [3] |
| 31 | CDH1 [2] |
| 32 | TNFSF13B [3] |
| 33 | TNFRSF17 [3] |
| 34 | CDCA3 [14,15] |
| 35 | XBP1 [6] |

**Reference**

1. Xiong, N.; Sun, Q. Identification of Stage-Related and Severity-Related Biomarkers and Exploration of Immune Landscape for Dengue by Comprehensive Analyses. *Virol. J.* **2022**, *19*, 1–17, doi:10.1186/s12985-022-01853-8.

2. Zeba, A.; Rajalingam, A.; Sekar, K.; Ganjiwale, A. Machine Learning-Based Gene Expression Biomarkers to Distinguish Zika and Dengue Virus Infections: Implications for Diagnosis. *VirusDisease* **2024**, *35*, 446–461, doi:10.1007/s13337-024-00885-8.

3. Josyula, J.V.N.; Talari, P.; Pillai, A.K.B.; Mutheneni, S.R. Analysis of Gene Expression Profile for Identification of Novel Gene Signatures during Dengue Infection. *Infect. Med.* **2023**, *2*, 19–30, doi:10.1016/j.imj.2023.02.002.

4. Paul, J.K.; Azmal, M.; Alam, T.; Talukder, O.F.; Ghosh, A. Comprehensive Analysis of Intervention and Control Studies for the Computational Identification of Dengue Biomarker Genes. *PLoS Negl. Trop. Dis.* **2025**, *19*, e0012914, doi:10.1371/journal.pntd.0012914.

5. Mosharaf, M.P.; Reza, M.S.; Kibria, M.K.; Ahmed, F.F.; Kabir, M.H.; Hasan, S.; Mollah, M.N.H. Computational Identification of Host Genomic Biomarkers Highlighting Their Functions, Pathways and Regulators That Influence SARS-CoV-2 Infections and Drug Repurposing. *Sci. Rep.* **2022**, *12*, 1–22, doi:10.1038/s41598-022-08073-8.

6. Li, J.; Yan, X.; Li, B.; Huang, L.; Wang, X.; He, B.; Xie, H.; Wu, Q.; Chen, L. Identification and Validation of Ferroptosis-Related Genes in Patients Infected with Dengue Virus: Implication in the Pathogenesis of DENV. *Virus Genes* **2023**, *59*, 377–390, doi:10.1007/s11262-023-01985-1.

7. Xie, L.M.; Yin, X.; Bi, J.; Luo, H.M.; Cao, X.J.; Ma, Y.W.; Liu, Y.L.; Su, J.W.; Lin, G.L.; Guo, X.G. Identification of Potential Biomarkers in Dengue via Integrated Bioinformatic Analysis. *PLoS Negl. Trop. Dis.* **2021**, *15*, 1–11, doi:10.1371/journal.pntd.0009633.

8. Xie, L.M.; Yin, X.; Bi, J.; Luo, H.M.; Cao, X.J.; Ma, Y.W.; Liu, Y.L.; Su, J.W.; Lin, G.L.; Guo, X.G. Identification of Potential Biomarkers in Dengue via Integrated Bioinformatic Analysis. *PLoS Negl. Trop. Dis.* **2021**, *15*, 1–15, doi:10.1371/journal.pntd.0009633.

9. Lin, R.-J.; Yu, H.-P.; Chang, B.-L.; Tang, W.-C.; Liao, C.-L.; Lin, Y.-L. Distinct Antiviral Roles for Human 2′,5′-Oligoadenylate Synthetase Family Members against Dengue Virus Infection. *J. Immunol.* **2009**, *183*, 8035–8043, doi:10.4049/jimmunol.0902728.

10. Suppiah, J.; Md Sani, S.S.; Hassan, S.S.; Nadzar, N.I.F.; Ibrahim, N. ‘Izzah; Thayan, R.; Mohd Zain, R. Unraveling Potential Gene Biomarkers for Dengue Infection through RNA Sequencing. *Virus Genes* **2024**, *61*, 26–37, doi:10.1007/s11262-024-02114-2.

11. Trobaugh, D.W.; Klimstra, W.B. MicroRNA Regulation of RNA Virus Replication and Pathogenesis. *Trends Mol. Med.* **2017**, *23*, 80–93, doi:10.1016/j.molmed.2016.11.003.

12. Hsu, Y.L.; Shi, S.F.; Wu, W.L.; Ho, L.J.; Lai, J.H. Protective Roles of Interferon-Induced Protein with Tetratricopeptide Repeats 3 (IFIT3) in Dengue Virus Infection of Human Lung Epithelial Cells. *PLoS One* **2013**, *8*, 1–13, doi:10.1371/journal.pone.0079518.

13. Murarik, M.R. Identification of Biomarkers for the Prediction of Dengue Disease Severity Using High- Throughput Proteomics. **2023**.

14. Winter, C.; Camarão, A.A.R.; Steffen, I.; Jung, K. Network Meta-Analysis of Transcriptome Expression Changes in Different Manifestations of Dengue Virus Infection. *BMC Genomics* **2022**, *23*, 1–15, doi:10.1186/s12864-022-08390-2.

15. Winter, C.; Camarão, A.A.R.; Steffen, I.; Jung, K. Network Meta-Analysis of Transcriptome Expression Changes in Different Manifestations of Dengue Virus Infection. *BMC Genomics* **2022**, *23*, 1–15, doi:10.1186/s12864-022-08390-2.

16. Alwabli, A.S. Deciphering the Molecular Landscape of Dengue Infection: Insights from Gene Expression Profiling and Protein Interactions. *Microbe (Netherlands)* **2024**, *5*, doi:10.1016/j.microb.2024.100195.
