# Supplementary material for: In-silico identification of host-key-genes associated with dengue-virus-infections highlighting their pathogenetic mechanisms and therapeutic agents
Source: PLoS One. 2025 Oct 7;20(10):e0333509. doi: 10.1371/journal.pone.0333509 (PMC12503274; doi:10.1371/journal.pone.0333509)
Supplement: S9 Table — (DOCX) [file pone.0333509.s010.docx]

**S9 Table.** Binding affinity score within suggested drugs and suggested protein (published by others, causing DENVI).

| **Gene** | **ENTRECTINIB** | **IMATINIB** | **QL47** | **Average** |
| --- | --- | --- | --- | --- |
| CD38 | -11.1 | -9.3 | -10.2 | -10.2 |
| ADH6 | -11.2 | -9.4 | -9.1 | -9.9 |
| CXCR3 | -10.1 | -8.8 | -10.1 | -9.66 |
| CYBB | -10.7 | -9 | -9.3 | -9.66 |
| IRF7 | -9.7 | -9.9 | -8.8 | -9.46 |
| HSPA5 | -9.5 | -9.2 | -9.4 | -9.36 |
| OAS1 | -9.9 | -8.9 | -8.6 | -9.36 |
| PTGS2 | -8.6 | -10.4 | -8.6 | -9.2 |
| STAT1 | -9.7 | -8.1 | -8.9 | -9.13 |
| SYK | -8.7 | -10 | -9.4 | -8.93 |
| ATF3 | -8.5 | -8.4 | -9.3 | -8.9 |
| MX1 | -10.3 | -8.7 | -7.8 | -8.8 |
| CDKN1C | -9.1 | -8.6 | -8.6 | -8.76 |
| IFI27 | -9.2 | -9.5 | -7.7 | -8.73 |
| SOCS3 | -9.1 | -8.4 | -7.7 | -8.53 |
| ANXA9 | -8.9 | -8.3 | -8.4 | -8.5 |
| NR0B2 | -9.8 | -8.1 | -7.6 | -8.4 |
| IFI44L | -8 | -8.8 | -8.3 | -8.4 |
| STAT2 | -8.5 | -8 | -8.1 | -8.36 |
| C1orf12 | -9 | -8 | -7.7 | -8.33 |
| CDT1 | -9.3 | -8.1 | -7.6 | -8.26 |
| IL6 | -9.5 | -8.4 | -7.3 | -8.23 |
| IFI35 | -9.2 | -8.1 | -7.5 | -8.2 |
| ISG15 | -8.7 | -7.9 | -7.7 | -8.16 |
| IFI6 | -8.9 | -7.7 | -7.9 | -8.1 |
| USP18 | -8 | -7.6 | -7.9 | -7.83 |
| KCTD14 | -8 | -7.7 | -7.6 | -7.76 |
| NFKB1 | -9 | -7.5 | -6.8 | -7.76 |
| CAV1 | -7.6 | -7.6 | -7.5 | -7.7 |
| IL12A | -7.8 | -7.9 | -7.4 | -7.56 |
| CDH1 | -7.6 | -7.6 | -7.4 | -7.53 |
| TNFSF13B | -7.5 | -7.8 | -7.1 | -7.46 |
| TNFRSF17 | -7.4 | -6.8 | -7 | -7.06 |
| CDCA3 | -6.2 | -6.7 | -6.9 | -6.6 |
| XBP1 | -6.7 | -6.4 | -6.3 | -6.46 |
