# Supplementary material for: In-silico identification of host-key-genes associated with dengue-virus-infections highlighting their pathogenetic mechanisms and therapeutic agents
Source: PLoS One. 2025 Oct 7;20(10):e0333509. doi: 10.1371/journal.pone.0333509 (PMC12503274; doi:10.1371/journal.pone.0333509)
Supplement: S10 Table — (DOCX) [file pone.0333509.s011.docx]

**S10 Table.** Scores of Molecular Dynamics (MD) Simulations.

|  | **RMSD** | | | | **RMSF** | | | | **MM-PBSA** | | | |
| --- | --- | --- | --- | --- | --- | --- | --- | --- | --- | --- | --- | --- |
|  | **Time (ns)** | **TYMS vs Imatinib** | **TYMS vs Entrectinib** | **CDC20 vs QL47** | **Residue** | **TYMS vs Imatinib** | **TYMS vs Entrectinib** | **CDC20 vs QL47** | **Time (ns)** | **TYMS vs Imatinib** | **TYMS vs Entrectinib** | **CDC20 vs QL47** |
| **Minimum** |  | **0.448** | **0.469** | **0.438** |  | **0.57** | **0.51** | **0.59** |  | **-53067.7** | **-53242.29** | **-47173.2** |
| **Maximum** |  | **3.729** | **3.112** | **1.49** |  | **6.89** | **9.97** | **3.25** |  | **-42314.9** | **-38025.09** | **-35241.1** |
| **Average** |  | **2.4175736** | **2.5514439** | **1.219786** |  | **1.3519** | **1.3882** | **1.1823** |  | **-43302.5** | **-39343.02** | **-35920.3** |
|  | 0 | 0.448 | 0.469 | 0.438 | 1 | 6.89 | 9.97 | 3.25 | 1 | -53067.7 | -53242.29 | -47173.2 |
|  | 0.25 | 1.09 | 1.24 | 0.967 | 2 | 5.01 | 6.81 | 1.31 | 2 | -42314.9 | -39575.55 | -36223.5 |
|  | 0.5 | 1.222 | 1.457 | 1.086 | 3 | 4.4 | 6.66 | 2.14 | 3 | -42673.6 | -39006.39 | -35863.2 |
|  | 0.75 | 1.982 | 1.475 | 1.168 | 4 | 2.78 | 4.39 | 1.57 | 4 | -42799.1 | -38419.12 | -35713.3 |
|  | 1 | 1.706 | 1.948 | 1.155 | 5 | 1.58 | 1.98 | 1.58 | 5 | -42658.7 | -38896.04 | -35606.7 |
|  | 1.25 | 2.226 | 1.92 | 1.133 | 6 | 0.78 | 0.76 | 1.38 | 6 | -42662.2 | -38557.22 | -35806 |
|  | 1.5 | 1.749 | 2.145 | 1.216 | 7 | 0.69 | 0.62 | 1.42 | 7 | -42451.2 | -38025.09 | -35728.8 |
|  | 1.75 | 1.908 | 1.933 | 1.211 | 8 | 0.57 | 0.51 | 0.88 | 8 | -43211.7 | -38038.23 | -35354.5 |
|  | 2 | 1.91 | 2.036 | 1.155 | 9 | 0.98 | 0.88 | 1 | 9 | -43059.2 | -38811.05 | -35606.1 |
|  | 2.25 | 1.547 | 2.206 | 1.251 | 10 | 1.27 | 0.91 | 1.65 | 10 | -43124.8 | -38996.54 | -35263.5 |
|  | 2.5 | 2.206 | 2.351 | 1.291 | 11 | 0.66 | 0.62 | 1.38 | 11 | -43636.4 | -38486.28 | -35284.6 |
|  | 2.75 | 2.074 | 1.984 | 1.197 | 12 | 0.85 | 0.8 | 1.19 | 12 | -42915.7 | -38820.72 | -35500.9 |
|  | 3 | 1.604 | 1.467 | 1.219 | 13 | 0.67 | 0.58 | 0.99 | 13 | -42387.3 | -39027.14 | -35241.1 |
|  | 3.25 | 2.314 | 1.858 | 1.151 | 14 | 0.78 | 0.71 | 0.79 | 14 | -43424.7 | -38949.83 | -35600.2 |
|  | 3.5 | 1.807 | 1.998 | 1.103 | 15 | 0.89 | 0.87 | 0.75 | 15 | -43518.2 | -39100.93 | -35610 |
|  | 3.75 | 1.919 | 2.067 | 1.179 | 16 | 1.4 | 1.3 | 1 | 16 | -42969.6 | -38761.23 | -36410.5 |
|  | 4 | 1.77 | 1.931 | 1.034 | 17 | 1.11 | 0.97 | 0.8 | 17 | -43095.5 | -39169.88 | -35600.2 |
|  | 4.25 | 1.597 | 2.228 | 1.018 | 18 | 0.92 | 0.83 | 1.09 | 18 | -43361.1 | -38992.3 | -36038.9 |
|  | 4.5 | 1.721 | 2.074 | 1.15 | 19 | 1.17 | 1.05 | 1.06 | 19 | -43118.7 | -38265.71 | -36230.9 |
|  | 4.75 | 1.55 | 2.105 | 1.189 | 20 | 2.18 | 2 | 0.76 | 20 | -43671.6 | -38599.59 | -35802.6 |
|  | 5 | 1.685 | 2.436 | 1.222 | 21 | 1.3 | 1.1 | 0.87 | 21 | -43271.4 | -38769.15 | -35742.8 |
|  | 5.25 | 1.642 | 2.306 | 1.095 | 22 | 1.03 | 0.82 | 1.11 | 22 | -42593.8 | -39198.75 | -35775.2 |
|  | 5.5 | 1.713 | 2.377 | 1.231 | 23 | 1.72 | 1.57 | 0.81 | 23 | -43619.8 | -39589.93 | -35658.1 |
|  | 5.75 | 2.142 | 2.767 | 1.173 | 24 | 2.27 | 2.03 | 0.64 | 24 | -43513.1 | -39509.44 | -35871.5 |
|  | 6 | 2.259 | 2.782 | 1.114 | 25 | 2.07 | 2.02 | 0.8 | 25 | -43737.6 | -39798.47 | -36024.8 |
|  | 6.25 | 2.124 | 2.618 | 1.258 | 26 | 2.18 | 2.36 | 1.21 | 26 | -43431.2 | -39352.65 | -35469.4 |
|  | 6.5 | 2.448 | 3.023 | 1.297 | 27 | 2.41 | 3.64 | 0.7 | 27 | -43249.8 | -39032.88 | -35819.8 |
|  | 6.75 | 2.167 | 2.662 | 1.28 | 28 | 2.78 | 2.95 | 0.69 | 28 | -43424.5 | -39701.75 | -35456.9 |
|  | 7 | 1.7 | 2.304 | 1.122 | 29 | 1.58 | 1.44 | 0.86 | 29 | -42720.2 | -39388.15 | -35642 |
|  | 7.25 | 1.901 | 2.179 | 1.46 | 30 | 1.28 | 1.09 | 0.98 | 30 | -42845.4 | -39222.83 | -35584.2 |
|  | 7.5 | 1.714 | 2.174 | 1.262 | 31 | 1.39 | 1.39 | 0.86 | 31 | -42985.6 | -39415.61 | -35690.5 |
|  | 7.75 | 1.871 | 2.31 | 1.156 | 32 | 1.1 | 0.98 | 1.18 | 32 | -42597.1 | -39507.77 | -35318.9 |
|  | 8 | 1.99 | 1.945 | 1.186 | 33 | 1.52 | 1.39 | 1 | 33 | -42949.8 | -39228.95 | -36035.4 |
|  | 8.25 | 1.854 | 2.176 | 1.179 | 34 | 1.59 | 1.61 | 1.15 | 34 | -42666.2 | -39958.84 | -35405 |
|  | 8.5 | 1.934 | 2.159 | 1.171 | 35 | 1.01 | 0.89 | 0.8 | 35 | -43237.7 | -39619.19 | -35819.1 |
|  | 8.75 | 1.949 | 2.382 | 1.198 | 36 | 1.36 | 1.23 | 1.09 | 36 | -42670.6 | -39587.15 | -35644.3 |
|  | 9 | 2.037 | 1.851 | 1.153 | 37 | 1.5 | 1.53 | 0.76 | 37 | -43382.9 | -39512.09 | -35777.8 |
|  | 9.25 | 1.951 | 1.924 | 1.213 | 38 | 0.87 | 0.78 | 0.83 | 38 | -43136.1 | -39866.11 | -36245.9 |
|  | 9.5 | 2.026 | 2.022 | 1.456 | 39 | 1.96 | 1.3 | 0.73 | 39 | -44082.4 | -39168.98 | -35959.6 |
|  | 9.75 | 1.936 | 1.75 | 1.369 | 40 | 0.83 | 0.66 | 0.96 | 40 | -43011.6 | -39125.02 | -36023 |
|  | 10 | 1.781 | 1.84 | 1.043 | 41 | 0.81 | 0.65 | 0.87 | 41 | -43366.5 | -39639.63 | -35939.5 |
|  | 10.25 | 1.943 | 1.865 | 1.071 | 42 | 0.98 | 0.81 | 0.94 | 42 | -43470.8 | -39360.77 | -36132.8 |
|  | 10.5 | 1.776 | 1.917 | 1.165 | 43 | 0.8 | 0.74 | 0.78 | 43 | -43750.6 | -39810.69 | -35978 |
|  | 10.75 | 1.884 | 1.853 | 1.2 | 44 | 1.24 | 1.13 | 1.04 | 44 | -43482.2 | -39260.44 | -35594.7 |
|  | 11 | 1.829 | 2.014 | 1.106 | 45 | 1.24 | 1.17 | 0.88 | 45 | -43400.3 | -39675.8 | -35621.5 |
|  | 11.25 | 1.643 | 1.949 | 1.178 | 46 | 0.72 | 0.63 | 1.31 | 46 | -43591.8 | -39050.68 | -36462.7 |
|  | 11.5 | 1.61 | 2.074 | 1.061 | 47 | 0.73 | 0.7 | 1.69 | 47 | -43652.2 | -39729.02 | -36103.7 |
|  | 11.75 | 1.844 | 2.057 | 1.107 | 48 | 1.16 | 0.9 | 1.78 | 48 | -43160.5 | -39726.82 | -35872.4 |
|  | 12 | 1.704 | 2.219 | 1.17 | 49 | 1.12 | 0.85 | 1.86 | 49 | -43491.3 | -39785.68 | -35740.1 |
|  | 12.25 | 1.686 | 2.457 | 1.262 | 50 | 0.7 | 0.69 | 1.29 | 50 | -43269.7 | -39631.68 | -35936.3 |
|  | 12.5 | 1.773 | 2.504 | 1.155 | 51 | 0.71 | 0.67 | 1.92 | 51 | -43159.6 | -39478.38 | -35733.7 |
|  | 12.75 | 1.798 | 2.359 | 1.143 | 52 | 1.29 | 1.32 | 1.89 | 52 | -43008.3 | -39642.81 | -36319.2 |
|  | 13 | 1.749 | 2.383 | 1.167 | 53 | 1.46 | 1.79 | 1.99 | 53 | -43446.2 | -40237.19 | -36244.6 |
|  | 13.25 | 1.574 | 2.156 | 1.131 | 54 | 0.96 | 0.97 | 1.2 | 54 | -43322.6 | -39956.56 | -36071.2 |
|  | 13.5 | 1.742 | 2.143 | 1.174 | 55 | 1.14 | 1.17 | 0.95 | 55 | -42738.6 | -39308.43 | -36131 |
|  | 13.75 | 1.704 | 2.275 | 1.144 | 56 | 1.48 | 1.05 | 1.12 | 56 | -42939.8 | -38985.27 | -35987.6 |
|  | 14 | 1.666 | 2.224 | 1.172 | 57 | 1.99 | 1.53 | 1.33 | 57 | -43827.1 | -38969.03 | -36065 |
|  | 14.25 | 1.618 | 2.292 | 1.208 | 58 | 0.63 | 0.65 | 1.05 | 58 | -43441.4 | -39221.86 | -36056.7 |
|  | 14.5 | 1.812 | 2.546 | 1.085 | 59 | 0.83 | 0.77 | 2.01 | 59 | -43586.5 | -39214.14 | -36602 |
|  | 14.75 | 1.523 | 2.511 | 1.186 | 60 | 0.99 | 0.81 | 1.84 | 60 | -43551.4 | -39372.2 | -35872.1 |
|  | 15 | 1.717 | 2.46 | 1.207 | 61 | 0.67 | 0.93 | 1.4 | 61 | -43534.7 | -39530.71 | -36202.3 |
|  | 15.25 | 1.712 | 2.142 | 1.1 | 62 | 0.66 | 0.6 | 0.99 | 62 | -43908.5 | -39589.73 | -36113.8 |
|  | 15.5 | 1.615 | 1.868 | 1.079 | 63 | 0.8 | 0.76 | 0.87 | 63 | -43223.6 | -39149.18 | -35898.1 |
|  | 15.75 | 1.663 | 2.189 | 1.076 | 64 | 0.81 | 0.78 | 0.6 | 64 | -43768.5 | -39175.66 | -36076.2 |
|  | 16 | 1.663 | 2.182 | 1.176 | 65 | 0.59 | 0.58 | 0.91 | 65 | -42618.8 | -39255.47 | -35947.6 |
|  | 16.25 | 1.987 | 2.186 | 1.11 | 66 | 1.13 | 1.42 | 0.6 | 66 | -43118.7 | -40077.98 | -35838 |
|  | 16.5 | 1.676 | 2.292 | 1.08 | 67 | 0.98 | 0.96 | 0.91 | 67 | -43489.7 | -39735.41 | -36178 |
|  | 16.75 | 1.698 | 2.234 | 1.054 | 68 | 1.4 | 1.68 | 1.19 | 68 | -42811.1 | -39460.12 | -36079.2 |
|  | 17 | 1.754 | 2.141 | 1.118 | 69 | 0.59 | 0.62 | 0.81 | 69 | -43786.1 | -39611.77 | -36057.7 |
|  | 17.25 | 1.766 | 2.583 | 1.081 | 70 | 0.7 | 0.82 | 0.67 | 70 | -43286.3 | -39325.02 | -36371.2 |
|  | 17.5 | 1.728 | 2.106 | 1.112 | 71 | 0.72 | 0.74 | 1.08 | 71 | -43068 | -39332.87 | -36071.1 |
|  | 17.75 | 1.659 | 2.232 | 1.142 | 72 | 0.75 | 0.77 | 2.21 | 72 | -44018.3 | -39231.32 | -36042 |
|  | 18 | 1.797 | 2.206 | 1.163 | 73 | 0.83 | 0.91 | 1.71 | 73 | -43876.7 | -39081.35 | -36253.6 |
|  | 18.25 | 2.052 | 2.502 | 1.245 | 74 | 1.5 | 2 | 0.89 | 74 | -43687.9 | -38668.16 | -35799.5 |
|  | 18.5 | 2.063 | 2.468 | 1.138 | 75 | 1.32 | 1.51 | 1.5 | 75 | -43057.6 | -38267.6 | -35645.2 |
|  | 18.75 | 1.889 | 2.257 | 1.13 | 76 | 0.92 | 0.85 | 1.22 | 76 | -42877.3 | -38780.08 | -35743.6 |
|  | 19 | 2.324 | 2.529 | 1.185 | 77 | 1.03 | 1.17 | 0.98 | 77 | -43386.7 | -39341.39 | -35579 |
|  | 19.25 | 2.342 | 2.803 | 1.3 | 78 | 1.29 | 1.6 | 0.78 | 78 | -43211.2 | -38751.25 | -35671.3 |
|  | 19.5 | 2.103 | 2.798 | 1.227 | 79 | 1.53 | 1.61 | 1.04 | 79 | -43037 | -39290.85 | -35657.3 |
|  | 19.75 | 1.843 | 2.476 | 1.187 | 80 | 1.2 | 1.08 | 0.59 | 80 | -43364.9 | -39194.51 | -35340.8 |
|  | 20 | 1.721 | 2.654 | 1.404 | 81 | 1.24 | 0.99 | 0.81 | 81 | -42701.6 | -39380.01 | -36170.4 |
|  | 20.25 | 1.695 | 2.528 | 1.297 | 82 | 2.25 | 1.84 | 0.65 | 82 | -43245.2 | -38786.48 | -35930.9 |
|  | 20.5 | 1.802 | 2.557 | 1.175 | 83 | 1.76 | 1.05 | 1.01 | 83 | -43408.4 | -39112.83 | -35327.3 |
|  | 20.75 | 1.412 | 2.379 | 1.266 | 84 | 0.96 | 0.8 | 1.12 | 84 | -43261.8 | -39345.96 | -35305 |
|  | 21 | 1.756 | 2.223 | 1.163 | 85 | 1.18 | 1.29 | 0.88 | 85 | -42710 | -39481.5 | -35475.4 |
|  | 21.25 | 2.009 | 2.518 | 1.39 | 86 | 1.25 | 1.25 | 0.96 | 86 | -43356.3 | -39222.77 | -35298 |
|  | 21.5 | 2.003 | 2.58 | 1.327 | 87 | 1.04 | 1.01 | 0.73 | 87 | -42807.5 | -38733.9 | -35507.8 |
|  | 21.75 | 1.864 | 2.416 | 1.228 | 88 | 0.92 | 0.92 | 1.46 | 88 | -42380.3 | -39232.19 | -35429.3 |
|  | 22 | 1.921 | 2.542 | 1.275 | 89 | 1.19 | 1.17 | 0.86 | 89 | -42701.2 | -39226.9 | -35451.8 |
|  | 22.25 | 1.927 | 2.624 | 1.223 | 90 | 1.15 | 1.03 | 1.14 | 90 | -43203.2 | -39410.19 | -35483.8 |
|  | 22.5 | 1.675 | 2.575 | 1.287 | 91 | 1.55 | 1.49 | 1.64 | 91 | -43228.9 | -38771.59 | -35761.5 |
|  | 22.75 | 1.76 | 2.506 | 1.176 | 92 | 1.42 | 1.11 | 2.18 | 92 | -43041.1 | -38662.1 | -35460.2 |
|  | 23 | 1.771 | 2.553 | 1.185 | 93 | 1.16 | 1.18 | 1.84 | 93 | -43299.5 | -38904.41 | -35304.2 |
|  | 23.25 | 1.766 | 2.55 | 1.242 | 94 | 1.3 | 1.19 | 1.74 | 94 | -43346.6 | -39276.43 | -35359.3 |
|  | 23.5 | 1.973 | 2.597 | 1.161 | 95 | 1.55 | 1.46 | 1.92 | 95 | -43610.4 | -38980.27 | -35316.3 |
|  | 23.75 | 2.415 | 2.765 | 1.19 | 96 | 1.62 | 1.4 | 1.12 | 96 | -43491.4 | -38679.59 | -36073.2 |
|  | 24 | 2.19 | 2.934 | 1.129 | 97 | 1.34 | 1.19 | 1.58 | 97 | -43200.4 | -39109 | -35951.9 |
|  | 24.25 | 1.792 | 2.989 | 1.31 | 98 | 1.34 | 1.02 | 1.86 | 98 | -43253.5 | -38849.57 | -36058.8 |
|  | 24.5 | 1.831 | 2.594 | 1.141 | 99 | 1.56 | 1.48 | 0.91 | 99 | -43283.7 | -38768.47 | -35895.6 |
|  | 24.75 | 1.748 | 2.781 | 1.183 | 100 | 1.68 | 1.56 | 1.11 | 100 | -42679.7 | -39165.91 | -35820.6 |
|  | 25 | 1.557 | 2.731 | 1.264 |  |  |  |  |  |  |  |  |
|  | 25.25 | 1.63 | 2.79 | 1.197 |  |  |  |  |  |  |  |  |
|  | 25.5 | 1.598 | 2.81 | 1.349 |  |  |  |  |  |  |  |  |
|  | 25.75 | 1.738 | 2.634 | 1.356 |  |  |  |  |  |  |  |  |
|  | 26 | 1.948 | 2.717 | 1.268 |  |  |  |  |  |  |  |  |
|  | 26.25 | 2.137 | 2.684 | 1.344 |  |  |  |  |  |  |  |  |
|  | 26.5 | 1.784 | 2.807 | 1.213 |  |  |  |  |  |  |  |  |
|  | 26.75 | 1.877 | 2.565 | 1.266 |  |  |  |  |  |  |  |  |
|  | 27 | 1.819 | 2.492 | 1.214 |  |  |  |  |  |  |  |  |
|  | 27.25 | 1.834 | 2.723 | 1.242 |  |  |  |  |  |  |  |  |
|  | 27.5 | 1.946 | 2.664 | 1.343 |  |  |  |  |  |  |  |  |
|  | 27.75 | 1.63 | 2.581 | 1.043 |  |  |  |  |  |  |  |  |
|  | 28 | 1.897 | 2.479 | 1.319 |  |  |  |  |  |  |  |  |
|  | 28.25 | 1.748 | 2.472 | 1.209 |  |  |  |  |  |  |  |  |
|  | 28.5 | 1.579 | 2.516 | 1.126 |  |  |  |  |  |  |  |  |
|  | 28.75 | 1.485 | 2.551 | 1.192 |  |  |  |  |  |  |  |  |
|  | 29 | 1.783 | 2.448 | 1.25 |  |  |  |  |  |  |  |  |
|  | 29.25 | 1.858 | 2.748 | 1.314 |  |  |  |  |  |  |  |  |
|  | 29.5 | 1.773 | 2.579 | 1.285 |  |  |  |  |  |  |  |  |
|  | 29.75 | 1.82 | 2.57 | 1.322 |  |  |  |  |  |  |  |  |
|  | 30 | 1.694 | 2.727 | 1.258 |  |  |  |  |  |  |  |  |
|  | 30.25 | 1.518 | 2.674 | 1.277 |  |  |  |  |  |  |  |  |
|  | 30.5 | 1.639 | 2.762 | 1.177 |  |  |  |  |  |  |  |  |
|  | 30.75 | 1.555 | 2.761 | 1.177 |  |  |  |  |  |  |  |  |
|  | 31 | 1.59 | 2.722 | 1.363 |  |  |  |  |  |  |  |  |
|  | 31.25 | 1.73 | 2.768 | 1.219 |  |  |  |  |  |  |  |  |
|  | 31.5 | 1.791 | 2.386 | 1.256 |  |  |  |  |  |  |  |  |
|  | 31.75 | 1.562 | 2.548 | 1.203 |  |  |  |  |  |  |  |  |
|  | 32 | 1.543 | 2.533 | 1.18 |  |  |  |  |  |  |  |  |
|  | 32.25 | 1.371 | 2.377 | 1.245 |  |  |  |  |  |  |  |  |
|  | 32.5 | 1.434 | 2.441 | 1.209 |  |  |  |  |  |  |  |  |
|  | 32.75 | 1.413 | 2.421 | 1.286 |  |  |  |  |  |  |  |  |
|  | 33 | 1.649 | 2.886 | 1.186 |  |  |  |  |  |  |  |  |
|  | 33.25 | 1.578 | 2.684 | 1.25 |  |  |  |  |  |  |  |  |
|  | 33.5 | 1.67 | 2.532 | 1.201 |  |  |  |  |  |  |  |  |
|  | 33.75 | 1.437 | 2.8 | 1.144 |  |  |  |  |  |  |  |  |
|  | 34 | 1.464 | 2.583 | 1.35 |  |  |  |  |  |  |  |  |
|  | 34.25 | 1.585 | 2.474 | 1.42 |  |  |  |  |  |  |  |  |
|  | 34.5 | 1.671 | 2.576 | 1.359 |  |  |  |  |  |  |  |  |
|  | 34.75 | 1.592 | 2.529 | 1.369 |  |  |  |  |  |  |  |  |
|  | 35 | 1.869 | 2.629 | 1.075 |  |  |  |  |  |  |  |  |
|  | 35.25 | 1.743 | 2.647 | 1.162 |  |  |  |  |  |  |  |  |
|  | 35.5 | 1.928 | 2.841 | 1.282 |  |  |  |  |  |  |  |  |
|  | 35.75 | 1.882 | 2.785 | 1.239 |  |  |  |  |  |  |  |  |
|  | 36 | 1.796 | 2.499 | 1.276 |  |  |  |  |  |  |  |  |
|  | 36.25 | 1.561 | 2.512 | 1.254 |  |  |  |  |  |  |  |  |
|  | 36.5 | 1.878 | 2.546 | 1.167 |  |  |  |  |  |  |  |  |
|  | 36.75 | 2.007 | 2.267 | 1.146 |  |  |  |  |  |  |  |  |
|  | 37 | 1.515 | 2.555 | 1.297 |  |  |  |  |  |  |  |  |
|  | 37.25 | 1.801 | 2.434 | 1.16 |  |  |  |  |  |  |  |  |
|  | 37.5 | 1.755 | 2.438 | 1.051 |  |  |  |  |  |  |  |  |
|  | 37.75 | 1.778 | 2.526 | 1.175 |  |  |  |  |  |  |  |  |
|  | 38 | 1.911 | 2.301 | 1.052 |  |  |  |  |  |  |  |  |
|  | 38.25 | 1.826 | 2.874 | 1.207 |  |  |  |  |  |  |  |  |
|  | 38.5 | 1.58 | 2.536 | 1.134 |  |  |  |  |  |  |  |  |
|  | 38.75 | 1.698 | 2.469 | 1.077 |  |  |  |  |  |  |  |  |
|  | 39 | 1.5 | 2.536 | 1.207 |  |  |  |  |  |  |  |  |
|  | 39.25 | 1.807 | 2.421 | 1.076 |  |  |  |  |  |  |  |  |
|  | 39.5 | 1.976 | 2.342 | 1.229 |  |  |  |  |  |  |  |  |
|  | 39.75 | 1.768 | 2.333 | 1.172 |  |  |  |  |  |  |  |  |
|  | 40 | 1.794 | 2.386 | 1.183 |  |  |  |  |  |  |  |  |
|  | 40.25 | 2.254 | 2.704 | 1.222 |  |  |  |  |  |  |  |  |
|  | 40.5 | 2.022 | 2.605 | 1.129 |  |  |  |  |  |  |  |  |
|  | 40.75 | 1.765 | 2.576 | 1.149 |  |  |  |  |  |  |  |  |
|  | 41 | 2.028 | 2.554 | 1.148 |  |  |  |  |  |  |  |  |
|  | 41.25 | 2.133 | 2.645 | 1.073 |  |  |  |  |  |  |  |  |
|  | 41.5 | 2.042 | 2.459 | 1.049 |  |  |  |  |  |  |  |  |
|  | 41.75 | 1.849 | 2.423 | 1.078 |  |  |  |  |  |  |  |  |
|  | 42 | 1.772 | 2.463 | 1.15 |  |  |  |  |  |  |  |  |
|  | 42.25 | 1.95 | 2.653 | 1.095 |  |  |  |  |  |  |  |  |
|  | 42.5 | 1.762 | 2.494 | 1.118 |  |  |  |  |  |  |  |  |
|  | 42.75 | 1.886 | 2.505 | 1.085 |  |  |  |  |  |  |  |  |
|  | 43 | 1.845 | 2.243 | 1.126 |  |  |  |  |  |  |  |  |
|  | 43.25 | 1.917 | 2.217 | 1.124 |  |  |  |  |  |  |  |  |
|  | 43.5 | 1.758 | 2.437 | 1.202 |  |  |  |  |  |  |  |  |
|  | 43.75 | 1.99 | 2.198 | 1.202 |  |  |  |  |  |  |  |  |
|  | 44 | 2.166 | 2.114 | 1.021 |  |  |  |  |  |  |  |  |
|  | 44.25 | 1.697 | 2.254 | 1.117 |  |  |  |  |  |  |  |  |
|  | 44.5 | 1.922 | 2.235 | 1.027 |  |  |  |  |  |  |  |  |
|  | 44.75 | 2.094 | 2.298 | 1.325 |  |  |  |  |  |  |  |  |
|  | 45 | 2.097 | 2.31 | 1.118 |  |  |  |  |  |  |  |  |
|  | 45.25 | 2.139 | 2.379 | 1.374 |  |  |  |  |  |  |  |  |
|  | 45.5 | 1.866 | 2.65 | 1.288 |  |  |  |  |  |  |  |  |
|  | 45.75 | 2.302 | 2.437 | 1.337 |  |  |  |  |  |  |  |  |
|  | 46 | 2.424 | 2.442 | 1.314 |  |  |  |  |  |  |  |  |
|  | 46.25 | 2.272 | 2.383 | 1.342 |  |  |  |  |  |  |  |  |
|  | 46.5 | 2.18 | 2.57 | 1.362 |  |  |  |  |  |  |  |  |
|  | 46.75 | 2.094 | 2.471 | 1.217 |  |  |  |  |  |  |  |  |
|  | 47 | 1.995 | 2.577 | 1.144 |  |  |  |  |  |  |  |  |
|  | 47.25 | 1.819 | 2.685 | 1.137 |  |  |  |  |  |  |  |  |
|  | 47.5 | 1.972 | 2.579 | 1.139 |  |  |  |  |  |  |  |  |
|  | 47.75 | 1.823 | 2.678 | 1.133 |  |  |  |  |  |  |  |  |
|  | 48 | 1.978 | 2.742 | 1.247 |  |  |  |  |  |  |  |  |
|  | 48.25 | 2.095 | 2.566 | 1.153 |  |  |  |  |  |  |  |  |
|  | 48.5 | 2.08 | 2.628 | 1.187 |  |  |  |  |  |  |  |  |
|  | 48.75 | 1.852 | 2.727 | 1.226 |  |  |  |  |  |  |  |  |
|  | 49 | 2.136 | 2.813 | 1.159 |  |  |  |  |  |  |  |  |
|  | 49.25 | 2.411 | 2.788 | 1.209 |  |  |  |  |  |  |  |  |
|  | 49.5 | 2.546 | 2.719 | 1.312 |  |  |  |  |  |  |  |  |
|  | 49.75 | 2.412 | 2.713 | 1.265 |  |  |  |  |  |  |  |  |
|  | 50 | 2.507 | 2.696 | 1.35 |  |  |  |  |  |  |  |  |
|  | 50.25 | 1.917 | 2.885 | 1.332 |  |  |  |  |  |  |  |  |
|  | 50.5 | 1.944 | 2.662 | 1.445 |  |  |  |  |  |  |  |  |
|  | 50.75 | 2.022 | 2.7 | 1.424 |  |  |  |  |  |  |  |  |
|  | 51 | 2.008 | 2.78 | 1.442 |  |  |  |  |  |  |  |  |
|  | 51.25 | 2.256 | 2.914 | 1.349 |  |  |  |  |  |  |  |  |
|  | 51.5 | 1.939 | 2.92 | 1.49 |  |  |  |  |  |  |  |  |
|  | 51.75 | 1.948 | 2.588 | 1.439 |  |  |  |  |  |  |  |  |
|  | 52 | 1.914 | 2.698 | 1.477 |  |  |  |  |  |  |  |  |
|  | 52.25 | 2.06 | 2.604 | 1.36 |  |  |  |  |  |  |  |  |
|  | 52.5 | 2.291 | 2.806 | 1.21 |  |  |  |  |  |  |  |  |
|  | 52.75 | 2.096 | 2.716 | 1.255 |  |  |  |  |  |  |  |  |
|  | 53 | 1.87 | 2.716 | 1.27 |  |  |  |  |  |  |  |  |
|  | 53.25 | 2.079 | 2.666 | 1.188 |  |  |  |  |  |  |  |  |
|  | 53.5 | 2.137 | 2.73 | 1.144 |  |  |  |  |  |  |  |  |
|  | 53.75 | 2.044 | 2.628 | 1.127 |  |  |  |  |  |  |  |  |
|  | 54 | 1.898 | 2.521 | 1.169 |  |  |  |  |  |  |  |  |
|  | 54.25 | 1.828 | 2.921 | 1.178 |  |  |  |  |  |  |  |  |
|  | 54.5 | 1.981 | 2.734 | 1.217 |  |  |  |  |  |  |  |  |
|  | 54.75 | 2.28 | 2.797 | 1.151 |  |  |  |  |  |  |  |  |
|  | 55 | 2.202 | 2.513 | 1.246 |  |  |  |  |  |  |  |  |
|  | 55.25 | 2.033 | 2.967 | 1.227 |  |  |  |  |  |  |  |  |
|  | 55.5 | 2.063 | 2.55 | 1.142 |  |  |  |  |  |  |  |  |
|  | 55.75 | 2.029 | 2.675 | 1.104 |  |  |  |  |  |  |  |  |
|  | 56 | 2.3 | 2.773 | 1.076 |  |  |  |  |  |  |  |  |
|  | 56.25 | 2.193 | 2.932 | 1.218 |  |  |  |  |  |  |  |  |
|  | 56.5 | 2.256 | 3.112 | 1.096 |  |  |  |  |  |  |  |  |
|  | 56.75 | 2.932 | 2.874 | 1.205 |  |  |  |  |  |  |  |  |
|  | 57 | 2.48 | 3.092 | 1.061 |  |  |  |  |  |  |  |  |
|  | 57.25 | 2.488 | 2.924 | 1.108 |  |  |  |  |  |  |  |  |
|  | 57.5 | 2.311 | 2.924 | 1.151 |  |  |  |  |  |  |  |  |
|  | 57.75 | 2.544 | 2.263 | 1.149 |  |  |  |  |  |  |  |  |
|  | 58 | 2.46 | 2.409 | 1.208 |  |  |  |  |  |  |  |  |
|  | 58.25 | 2.809 | 2.45 | 1.198 |  |  |  |  |  |  |  |  |
|  | 58.5 | 2.906 | 2.729 | 1.049 |  |  |  |  |  |  |  |  |
|  | 58.75 | 2.659 | 2.52 | 1.063 |  |  |  |  |  |  |  |  |
|  | 59 | 2.676 | 2.461 | 1.109 |  |  |  |  |  |  |  |  |
|  | 59.25 | 3.201 | 2.664 | 1.225 |  |  |  |  |  |  |  |  |
|  | 59.5 | 2.311 | 2.637 | 1.118 |  |  |  |  |  |  |  |  |
|  | 59.75 | 2.311 | 2.675 | 1.175 |  |  |  |  |  |  |  |  |
|  | 60 | 2.201 | 2.717 | 1.182 |  |  |  |  |  |  |  |  |
|  | 60.25 | 2.253 | 2.495 | 1.292 |  |  |  |  |  |  |  |  |
|  | 60.5 | 2.662 | 2.617 | 1.322 |  |  |  |  |  |  |  |  |
|  | 60.75 | 2.591 | 2.621 | 1.246 |  |  |  |  |  |  |  |  |
|  | 61 | 2.408 | 2.548 | 1.206 |  |  |  |  |  |  |  |  |
|  | 61.25 | 2.646 | 2.678 | 1.178 |  |  |  |  |  |  |  |  |
|  | 61.5 | 2.924 | 2.668 | 1.173 |  |  |  |  |  |  |  |  |
|  | 61.75 | 3.027 | 2.463 | 1.206 |  |  |  |  |  |  |  |  |
|  | 62 | 3.154 | 2.61 | 1.216 |  |  |  |  |  |  |  |  |
|  | 62.25 | 2.979 | 2.742 | 1.191 |  |  |  |  |  |  |  |  |
|  | 62.5 | 2.94 | 2.721 | 1.241 |  |  |  |  |  |  |  |  |
|  | 62.75 | 2.583 | 2.6 | 1.269 |  |  |  |  |  |  |  |  |
|  | 63 | 2.683 | 2.706 | 1.283 |  |  |  |  |  |  |  |  |
|  | 63.25 | 3.141 | 2.52 | 1.228 |  |  |  |  |  |  |  |  |
|  | 63.5 | 3.146 | 2.625 | 1.218 |  |  |  |  |  |  |  |  |
|  | 63.75 | 3.078 | 2.757 | 1.108 |  |  |  |  |  |  |  |  |
|  | 64 | 2.919 | 2.616 | 1.246 |  |  |  |  |  |  |  |  |
|  | 64.25 | 2.723 | 2.638 | 1.215 |  |  |  |  |  |  |  |  |
|  | 64.5 | 2.597 | 2.536 | 1.27 |  |  |  |  |  |  |  |  |
|  | 64.75 | 2.727 | 2.731 | 1.463 |  |  |  |  |  |  |  |  |
|  | 65 | 2.658 | 2.552 | 1.343 |  |  |  |  |  |  |  |  |
|  | 65.25 | 2.452 | 2.82 | 1.26 |  |  |  |  |  |  |  |  |
|  | 65.5 | 2.535 | 2.799 | 1.28 |  |  |  |  |  |  |  |  |
|  | 65.75 | 2.325 | 2.763 | 1.373 |  |  |  |  |  |  |  |  |
|  | 66 | 2.709 | 2.811 | 1.287 |  |  |  |  |  |  |  |  |
|  | 66.25 | 2.903 | 2.531 | 1.314 |  |  |  |  |  |  |  |  |
|  | 66.5 | 2.847 | 2.678 | 1.409 |  |  |  |  |  |  |  |  |
|  | 66.75 | 2.831 | 2.711 | 1.185 |  |  |  |  |  |  |  |  |
|  | 67 | 2.978 | 2.827 | 1.35 |  |  |  |  |  |  |  |  |
|  | 67.25 | 3.096 | 2.954 | 1.236 |  |  |  |  |  |  |  |  |
|  | 67.5 | 3.318 | 3.037 | 1.343 |  |  |  |  |  |  |  |  |
|  | 67.75 | 3.017 | 2.537 | 1.176 |  |  |  |  |  |  |  |  |
|  | 68 | 3.096 | 2.832 | 1.189 |  |  |  |  |  |  |  |  |
|  | 68.25 | 3.017 | 2.795 | 1.121 |  |  |  |  |  |  |  |  |
|  | 68.5 | 3.061 | 2.771 | 1.139 |  |  |  |  |  |  |  |  |
|  | 68.75 | 3.096 | 2.9 | 1.092 |  |  |  |  |  |  |  |  |
|  | 69 | 3.096 | 2.979 | 1.022 |  |  |  |  |  |  |  |  |
|  | 69.25 | 3.061 | 2.667 | 1.136 |  |  |  |  |  |  |  |  |
|  | 69.5 | 3.061 | 2.77 | 1.113 |  |  |  |  |  |  |  |  |
|  | 69.75 | 3.017 | 2.587 | 1.1 |  |  |  |  |  |  |  |  |
|  | 70 | 3.096 | 2.623 | 0.971 |  |  |  |  |  |  |  |  |
|  | 70.25 | 3.061 | 3.028 | 1.129 |  |  |  |  |  |  |  |  |
|  | 70.5 | 3.096 | 2.954 | 1.217 |  |  |  |  |  |  |  |  |
|  | 70.75 | 3.061 | 2.921 | 1.336 |  |  |  |  |  |  |  |  |
|  | 71 | 3.017 | 2.846 | 1.167 |  |  |  |  |  |  |  |  |
|  | 71.25 | 2.917 | 2.853 | 1.244 |  |  |  |  |  |  |  |  |
|  | 71.5 | 2.588 | 2.921 | 1.112 |  |  |  |  |  |  |  |  |
|  | 71.75 | 2.652 | 2.73 | 1.073 |  |  |  |  |  |  |  |  |
|  | 72 | 3.28 | 2.41 | 1.185 |  |  |  |  |  |  |  |  |
|  | 72.25 | 3.321 | 2.644 | 1.258 |  |  |  |  |  |  |  |  |
|  | 72.5 | 3.061 | 2.645 | 1.307 |  |  |  |  |  |  |  |  |
|  | 72.75 | 3.478 | 2.744 | 1.313 |  |  |  |  |  |  |  |  |
|  | 73 | 3.061 | 2.552 | 1.3 |  |  |  |  |  |  |  |  |
|  | 73.25 | 3.421 | 2.812 | 1.236 |  |  |  |  |  |  |  |  |
|  | 73.5 | 3.427 | 2.62 | 1.485 |  |  |  |  |  |  |  |  |
|  | 73.75 | 3.061 | 2.897 | 1.294 |  |  |  |  |  |  |  |  |
|  | 74 | 3.077 | 2.519 | 1.367 |  |  |  |  |  |  |  |  |
|  | 74.25 | 3.48 | 2.646 | 1.405 |  |  |  |  |  |  |  |  |
|  | 74.5 | 3.437 | 2.798 | 1.23 |  |  |  |  |  |  |  |  |
|  | 74.75 | 3.321 | 2.72 | 1.424 |  |  |  |  |  |  |  |  |
|  | 75 | 3.077 | 2.549 | 1.286 |  |  |  |  |  |  |  |  |
|  | 75.25 | 3.061 | 2.527 | 1.31 |  |  |  |  |  |  |  |  |
|  | 75.5 | 3.077 | 2.698 | 1.349 |  |  |  |  |  |  |  |  |
|  | 75.75 | 3.171 | 2.632 | 1.294 |  |  |  |  |  |  |  |  |
|  | 76 | 2.971 | 2.835 | 1.444 |  |  |  |  |  |  |  |  |
|  | 76.25 | 3.147 | 2.925 | 1.437 |  |  |  |  |  |  |  |  |
|  | 76.5 | 2.816 | 2.575 | 1.25 |  |  |  |  |  |  |  |  |
|  | 76.75 | 2.859 | 2.584 | 1.293 |  |  |  |  |  |  |  |  |
|  | 77 | 2.872 | 2.763 | 1.27 |  |  |  |  |  |  |  |  |
|  | 77.25 | 2.89 | 2.521 | 1.382 |  |  |  |  |  |  |  |  |
|  | 77.5 | 2.924 | 2.578 | 1.258 |  |  |  |  |  |  |  |  |
|  | 77.75 | 2.822 | 2.507 | 1.385 |  |  |  |  |  |  |  |  |
|  | 78 | 3.078 | 2.816 | 1.316 |  |  |  |  |  |  |  |  |
|  | 78.25 | 2.847 | 2.653 | 1.257 |  |  |  |  |  |  |  |  |
|  | 78.5 | 2.826 | 2.547 | 1.378 |  |  |  |  |  |  |  |  |
|  | 78.75 | 3.25 | 2.476 | 1.346 |  |  |  |  |  |  |  |  |
|  | 79 | 3.219 | 2.429 | 1.272 |  |  |  |  |  |  |  |  |
|  | 79.25 | 3.247 | 2.73 | 1.187 |  |  |  |  |  |  |  |  |
|  | 79.5 | 3.339 | 2.445 | 1.222 |  |  |  |  |  |  |  |  |
|  | 79.75 | 3.266 | 2.869 | 1.38 |  |  |  |  |  |  |  |  |
|  | 80 | 3.42 | 2.656 | 1.336 |  |  |  |  |  |  |  |  |
|  | 80.25 | 3.436 | 2.769 | 1.328 |  |  |  |  |  |  |  |  |
|  | 80.5 | 3.404 | 2.498 | 1.286 |  |  |  |  |  |  |  |  |
|  | 80.75 | 3.257 | 2.423 | 1.296 |  |  |  |  |  |  |  |  |
|  | 81 | 3.281 | 2.742 | 1.273 |  |  |  |  |  |  |  |  |
|  | 81.25 | 3.291 | 2.972 | 1.226 |  |  |  |  |  |  |  |  |
|  | 81.5 | 3.245 | 2.837 | 1.176 |  |  |  |  |  |  |  |  |
|  | 81.75 | 3.351 | 2.656 | 1.208 |  |  |  |  |  |  |  |  |
|  | 82 | 3.25 | 2.462 | 1.383 |  |  |  |  |  |  |  |  |
|  | 82.25 | 3.219 | 2.518 | 1.328 |  |  |  |  |  |  |  |  |
|  | 82.5 | 3.247 | 2.536 | 1.321 |  |  |  |  |  |  |  |  |
|  | 82.75 | 3.339 | 2.583 | 1.263 |  |  |  |  |  |  |  |  |
|  | 83 | 3.266 | 2.594 | 1.248 |  |  |  |  |  |  |  |  |
|  | 83.25 | 3.42 | 2.545 | 1.324 |  |  |  |  |  |  |  |  |
|  | 83.5 | 3.329 | 2.413 | 1.291 |  |  |  |  |  |  |  |  |
|  | 83.75 | 3.255 | 2.972 | 1.138 |  |  |  |  |  |  |  |  |
|  | 84 | 3.245 | 3.062 | 1.324 |  |  |  |  |  |  |  |  |
|  | 84.25 | 3.4 | 2.844 | 1.309 |  |  |  |  |  |  |  |  |
|  | 84.5 | 3.372 | 2.722 | 1.336 |  |  |  |  |  |  |  |  |
|  | 84.75 | 3.355 | 2.407 | 1.139 |  |  |  |  |  |  |  |  |
|  | 85 | 3.456 | 2.594 | 1.16 |  |  |  |  |  |  |  |  |
|  | 85.25 | 3.343 | 2.631 | 1.211 |  |  |  |  |  |  |  |  |
|  | 85.5 | 3.401 | 2.325 | 1.15 |  |  |  |  |  |  |  |  |
|  | 85.75 | 3.277 | 2.617 | 1.209 |  |  |  |  |  |  |  |  |
|  | 86 | 3.321 | 2.661 | 1.146 |  |  |  |  |  |  |  |  |
|  | 86.25 | 3.204 | 2.5 | 1.204 |  |  |  |  |  |  |  |  |
|  | 86.5 | 3.393 | 2.634 | 1.323 |  |  |  |  |  |  |  |  |
|  | 86.75 | 3.548 | 2.762 | 1.183 |  |  |  |  |  |  |  |  |
|  | 87 | 3.331 | 2.666 | 1.238 |  |  |  |  |  |  |  |  |
|  | 87.25 | 3.472 | 2.469 | 1.287 |  |  |  |  |  |  |  |  |
|  | 87.5 | 3.275 | 2.676 | 1.225 |  |  |  |  |  |  |  |  |
|  | 87.75 | 3.489 | 2.663 | 1.241 |  |  |  |  |  |  |  |  |
|  | 88 | 3.318 | 2.848 | 1.186 |  |  |  |  |  |  |  |  |
|  | 88.25 | 3.41 | 2.556 | 1.321 |  |  |  |  |  |  |  |  |
|  | 88.5 | 3.558 | 2.46 | 1.282 |  |  |  |  |  |  |  |  |
|  | 88.75 | 3.363 | 2.795 | 1.261 |  |  |  |  |  |  |  |  |
|  | 89 | 3.415 | 2.761 | 1.308 |  |  |  |  |  |  |  |  |
|  | 89.25 | 3.382 | 2.716 | 1.23 |  |  |  |  |  |  |  |  |
|  | 89.5 | 3.439 | 2.543 | 1.33 |  |  |  |  |  |  |  |  |
|  | 89.75 | 3.346 | 2.869 | 1.088 |  |  |  |  |  |  |  |  |
|  | 90 | 3.5 | 2.564 | 1.232 |  |  |  |  |  |  |  |  |
|  | 90.25 | 3.432 | 2.612 | 1.25 |  |  |  |  |  |  |  |  |
|  | 90.5 | 3.459 | 2.662 | 1.233 |  |  |  |  |  |  |  |  |
|  | 90.75 | 3.419 | 2.811 | 1.293 |  |  |  |  |  |  |  |  |
|  | 91 | 3.539 | 2.597 | 1.202 |  |  |  |  |  |  |  |  |
|  | 91.25 | 3.489 | 2.656 | 1.228 |  |  |  |  |  |  |  |  |
|  | 91.5 | 3.51 | 2.641 | 1.265 |  |  |  |  |  |  |  |  |
|  | 91.75 | 3.369 | 2.585 | 1.285 |  |  |  |  |  |  |  |  |
|  | 92 | 3.263 | 2.567 | 1.24 |  |  |  |  |  |  |  |  |
|  | 92.25 | 3.28 | 2.712 | 1.269 |  |  |  |  |  |  |  |  |
|  | 92.5 | 3.289 | 2.797 | 1.188 |  |  |  |  |  |  |  |  |
|  | 92.75 | 3.34 | 2.565 | 1.269 |  |  |  |  |  |  |  |  |
|  | 93 | 3.666 | 2.706 | 1.282 |  |  |  |  |  |  |  |  |
|  | 93.25 | 3.368 | 2.756 | 1.19 |  |  |  |  |  |  |  |  |
|  | 93.5 | 3.475 | 2.727 | 1.199 |  |  |  |  |  |  |  |  |
|  | 93.75 | 3.317 | 2.889 | 1.194 |  |  |  |  |  |  |  |  |
|  | 94 | 3.614 | 2.848 | 1.304 |  |  |  |  |  |  |  |  |
|  | 94.25 | 3.5 | 2.862 | 1.269 |  |  |  |  |  |  |  |  |
|  | 94.5 | 3.211 | 2.778 | 1.257 |  |  |  |  |  |  |  |  |
|  | 94.75 | 3.457 | 2.795 | 1.224 |  |  |  |  |  |  |  |  |
|  | 95 | 3.402 | 2.889 | 1.285 |  |  |  |  |  |  |  |  |
|  | 95.25 | 3.491 | 2.814 | 1.253 |  |  |  |  |  |  |  |  |
|  | 95.5 | 3.612 | 2.705 | 1.095 |  |  |  |  |  |  |  |  |
|  | 95.75 | 3.516 | 2.723 | 1.152 |  |  |  |  |  |  |  |  |
|  | 96 | 3.428 | 2.782 | 1.15 |  |  |  |  |  |  |  |  |
|  | 96.25 | 3.502 | 2.597 | 1.246 |  |  |  |  |  |  |  |  |
|  | 96.5 | 3.334 | 2.698 | 1.3 |  |  |  |  |  |  |  |  |
|  | 96.75 | 3.352 | 2.699 | 1.258 |  |  |  |  |  |  |  |  |
|  | 97 | 3.53 | 2.478 | 1.319 |  |  |  |  |  |  |  |  |
|  | 97.25 | 3.48 | 2.713 | 1.34 |  |  |  |  |  |  |  |  |
|  | 97.5 | 3.516 | 2.718 | 1.159 |  |  |  |  |  |  |  |  |
|  | 97.75 | 3.635 | 2.858 | 1.207 |  |  |  |  |  |  |  |  |
|  | 98 | 3.633 | 2.802 | 1.241 |  |  |  |  |  |  |  |  |
|  | 98.25 | 3.729 | 2.631 | 1.193 |  |  |  |  |  |  |  |  |
|  | 98.5 | 3.582 | 2.866 | 1.207 |  |  |  |  |  |  |  |  |
|  | 98.75 | 3.643 | 2.638 | 1.289 |  |  |  |  |  |  |  |  |
|  | 99 | 3.39 | 2.873 | 1.325 |  |  |  |  |  |  |  |  |
|  | 99.25 | 3.472 | 3.077 | 1.249 |  |  |  |  |  |  |  |  |
|  | 99.5 | 3.529 | 2.838 | 1.404 |  |  |  |  |  |  |  |  |
|  | 99.75 | 3.412 | 2.936 | 1.214 |  |  |  |  |  |  |  |  |
|  | 100 | 3.449 | 2.657 | 1.259 |  |  |  |  |  |  |  |  |
